# A Review on Advances in the Use of Raw and Modified Agricultural Lignocellulosic Residues in Mono- and Multicomponent Continuous Adsorption of Inorganic Pollutants for Upscaling Technologies

**DOI:** 10.3390/polym17070953

**Published:** 2025-03-31

**Authors:** Ricardo Silva Coelho, Liliane Catone Soares, Oscar Fernando Herrera Adarme, Luisa Cardoso Maia, Camila Stéfanne Dias Costa, Eric Guibal, Leandro Vinícius Alves Gurgel

**Affiliations:** 1 Group of Physical Organic Chemistry, Department of Chemistry, Institute of Exact and Biological Sciences, Federal University of Ouro Preto, Campus Universitário Morro do Cruzeiro, Rua Quatro, 786, Bauxita, Ouro Preto 35402-136, MG, Brazil; ricardo.coelho@aluno.ufop.edu.br (R.S.C.); liliane.catone@ufop.edu.br (L.C.S.); luisa.maia@ufop.edu.br (L.C.M.); camilasdcosta@gmail.com (C.S.D.C.); 2 Environmental Engineering Graduate Program (ProAmb), School of Mines, Federal University of Ouro Preto, Campus Universitário Morro do Cruzeiro, Rua Nove, s/n, Bauxita, Ouro Preto 35402-163, MG, Brazil; 3 Faculdade de Engenharia Agrícola, Universidade Estadual de Campinas (Unicamp), Av. Cândido Rondon, 501, Campinas 13083-875, SP, Brazil; oscarf@unicamp.br; 4 Polymers Composites and Hybrids (PCH), IMT Mines Ales, 30100 Ales, France; eric.guibal@mines-ales.fr

**Keywords:** bioadsorbent, competitive adsorption, fixed-bed column, modeling, overshooting, water treatment

## Abstract

Using raw and modified lignocellulosic residues as bioadsorbents in continuous adsorption is challenging but it marks significant progress in water treatment and the transition to a bio-based circular economy. This study reviews the application of bioadsorbents in fixed-bed columns for treating water contaminated with inorganic species, offering guidance for future research. It evaluates chemical modifications to enhance adsorptive properties, explores adsorption mechanisms, and analyzes bioadsorbent performance under competitive adsorption conditions. Analysis of adsorption data included evaluation of adsorption capacity in mono- and multicomponent solutions, regeneration, reuse, bed efficiency, and disposal of spent bioadsorbents. This enabled assessing their scalability to sufficiently high levels of maturity for commercialization. In multicomponent solutions, selectivity was influenced by the characteristics of the bioadsorbents and by competitive adsorption among inorganic species. This affected adsorption performance, increasing the complexity of breakthrough curve modeling and controlling the biomaterial selectivity. Models for mono- and multicomponent systems are presented, including mass transfer equations and alternatives including “bell-type” equations for overshooting phenomena and innovative approaches using artificial neural networks and machine learning. The criteria discussed will assist in improving studies conducted from cradle (synthesis of new biomaterials) to grave (end use or disposal), contributing to accurate decision making for transferring the developed technology to an industrial scale and evaluating the technical and economic feasibility of bioadsorbents.

## 1. Introduction

Lignocellulosic materials are structural organic compounds found in plant biomass tissues, available as agro-industrial byproducts or residues, which can be used to produce bioenergy and bio-based chemicals. They are predominantly composed of cellulose (a homopolymer of β-D-anhydroglucose units (AGU) linked by β(1→4) glycosidic bonds) and hemicelluloses (heteropolymers of sugars with six and five carbons, such as β-D-xylose, α-L-arabinose, β-D-galactose, β-D-mannose, and uronic acids). Both cellulose and hemicelluloses form a matrix interspersed with lignin, a macromolecule composed of phenolic units, such as S unit (syringyl), G unit (guaiacyl), and H unit (*p*-hydroxyphenyl), linked by aryl-alkyl ether bonds, such as α-O-4, β-O-4, and 4-O-5, among others, and carbon-carbon bonds, such as β-β, β-5, and 5-5, among others. These structurally organized compounds provide strength, flexibility, and protection (physical, chemical, and biological) to the lignocellulosic biomass [1].

The presence of reactive hydroxyl groups, especially in cellulose at carbons C-2, C-3, and C-6 of AGU, enables a variety of chemical modifications in lignocellulosic materials, allowing them to perform as efficient bioadsorbents for the removal of contaminants from water [2].

Focusing on adsorption, raw and chemically modified lignocellulosic materials (examples are agricultural waste, vegetable peelings, grass, softwood, and hardwood) have been widely investigated and applied for the removal of inorganic contaminants from water. This approach can be highlighted as an attractive way to achieve excellent performance, without the disadvantages of many current inorganic-based commercial adsorbents [3]. The use of lignocellulosic biomass-based adsorbents can contribute to achieving a new profile of society, based on the concepts of green chemistry and circular bioeconomy [4]. Lignocellulosic biomass is abundant, easily accessible, inexpensive, renewable, and biodegradable. The biodegradability and structural composition of bioadsorbents enable their utilization for production of value-added products at the end of their service life. These products include biofuels, fertilizers, soil amendments, biochars, catalysts, nanomaterials, and structural molecules for synthesis. They can also be used for the cogeneration of energy, with controlled emissions, or for incorporation into the soil, as traps for carbon dioxide [5].

Adsorption is mainly carried out in batch and continuous modes [6,7]. Batch adsorption involves operation in a closed regime, with no input or output of matter during the process. It is generally first performed at the laboratory scale, to optimize the operating conditions, and then on a larger scale in an experimental or pre-pilot treatment plant [6,7]. Continuous adsorption uses an open regime, with input and output of matter during operation [8]. Continuous adsorption using a column is also first performed at the laboratory scale, to optimize the process, and then on a larger scale, employing configurations close to those found in industries. Column adsorption allows the treatment of large volumes of water or industrial effluent, at a lower cost than batch adsorption, making it more suitable for industrial application [8].

Mathematical modeling is very important for designing, simulating, modeling, and monitoring the industrial-scale operation of an adsorption column. Monocomponent adsorption in a fixed-bed column can be modeled using analytical equations [9]. However, multicomponent adsorption, which is typically the case in water treatment, involves the use of nonlinear kinetic and isotherm equations. Therefore, it is not possible to obtain a rigorous solution using analytical methods, requiring the use of numerical methods [10]. Common simplified models applied for continuous adsorption in fixed-bed columns include the Bohart–Adams [11], Thomas [12], Yoon–Nelson [13], Wolborska [14], and Bed Depth Service Time (BDST) equations (Supplementary Materials). These simplified models have as their main characteristics the assumption that there is local equilibrium at any cross section along the bed and normally neglect resistance to intraparticle diffusion and axial dispersion. However, due to the propagation of incorrect information in the literature, or lack of mathematical and computational skills, many authors mistakenly compare these continuous adsorption models, despite their simplified forms being mathematically identical [15].

The complexity of the column adsorption operation with water containing more than one component is mainly due to competitive adsorption [16]. Adsorbates with higher affinity will tend to occupy the adsorption sites, causing the desorption of previously adsorbed adsorbates with lower affinity for the adsorption sites [16]. In addition, multicomponent column adsorption models use equilibrium relationships (isotherms) with coupled partial differential equations (fluid phase mass balance and adsorption rate (mass transfer resistances)) (Supplementary Materials), as a function of time and space, to describe the dynamics of the continuous adsorption process [10]. Considering the degree of complexity in solving such systems of equations, it is common and desirable to use more simplified models that can reduce the programming effort and computational time [17].

In addition to modeling, further advances in water treatment technologies require understanding of the behavior of lignocellulosic adsorbents in fixed-bed columns. There are few reported studies concerning fixed-bed column adsorption experiments using raw and modified lignocellulosic materials, while even fewer articles provide detailed information about the behaviors of these materials in column adsorption experiments. There is a scarcity of modeling studies for raw and modified lignocellulosic materials used in fixed-bed column adsorption. In-depth studies are needed to understand adsorption behavior in water containing multiple contaminants, including under relevant environmental conditions.

For optimization of column operating conditions, it is essential to evaluate not only the adsorption capacity of saturated materials, but also other aspects including resistance to mass transfer (including length of the mass transfer zone), the effective use of the bed, and the efficiency of adsorption in a single column or in a series of columns. Therefore, it is necessary to work with industrially relevant operating parameters and configurations to advance the technology readiness level (TRL) and enable the transfer of the developed technology to the market. Not least, it is also necessary to evaluate the reuse and final or other destination for depleted bioadsorbents, considering the technical and economic feasibility of industrial bioadsorbent reuse, together with the concepts of a circular bioeconomy.

This review presents the main studies available in the literature concerning the use of raw and modified lignocellulosic adsorbents for the removal of inorganic contaminants from water using fixed-bed columns. This review also discusses the following issues:

1. The main bottlenecks that prevent major advances in the technology readiness level (TRL > 5, at mini-pilot or pilot scales in a relevant environment) and upscaling of the technologies.
2. The influence of chemical modifications on the physicochemical properties and behaviors of bioadsorbents in fixed-bed columns.
3. The problem of using simplified model equations for modeling monocomponent adsorption in fixed-bed columns.
4. The complexity of competitive adsorption and column behavior in multicomponent adsorption, with possible solutions arising in the near future due to the introduction of artificial neural networks and machine learning methods to simulate adsorption operation, including efficiency of separation.
5. The recovery and destination of depleted bioadsorbents after the treatment of water containing inorganic contaminants, with possible second uses in industry, strengthening the concepts of a circular bioeconomy.

## 2. Methodology: A Bibliometric Approach

A search was performed on the “Web of Science Core Collection” database (Clarivate Analytics®, Boston, MA, USA), using the following search equation with Boolean operators: all = ((column or continuous or pack*) and (adsorb* and capacit*) and (stalk or shell or seed or husk or straw or waste* or bran or leaves or chaff or hauls or peel or bagasse or root or sawdust or fiber or residue or agricul*)). This search covered a period of 13 years, from 2012 to 2025, and aimed to identify the most recent trends related to the use of lignocellulosic materials as bioadsorbents. A first, more general, bibliometric analysis, not specific only to lignocellulosic materials, was carried out, resulting in a total of 3199 documents (Supplementary Materials). This first analysis concerned worldwide studies of adsorption using non-conventional adsorbents and sustainable adsorbents. The retrieved documents were analyzed and manually selected, according to title and abstract, for a second and more specific bibliometric analysis. Use of the multicriteria decision-making methodology called Methodi Ordinatio provided a systematic review that allowed the selection and proper classification of the compiled bibliography, considering the Journal Citation Reports (JCR) associated with the journal where the article was published, number of citations (NC), and year of publication (YP). This optimized screening was enabled by the InOrdinatio equation generated by three variables (JCR, NC, and YP), which indicated the scientific relevance of each study [18]. The InOrdinatio index is calculated using Equation (1), where α is a relevance fitting parameter, with a suggested value of 10 [18].

*InOrdinatio* = *JCR* + *NC* + α (current year-year of publication) (1)

Excel® 2016 software (Microsoft, Redmond, WA, USA) was used to analyze the summarized portfolio, which was later enriched with other articles that had not been classified in the first bibliometric analysis (Supplementary Materials), but were considered relevant in a second evaluation.

## 3. Technologies for Removal of Inorganic Pollutants from Water

Trace-level inorganic pollutants include cations and anions of various chemical species. Treating water contaminated with these compounds presents a significant environmental and industrial challenge, requiring effective economically, environmentally, and technically feasible solutions. Several techniques have been evaluated to remove inorganic pollutants from water, each one with its advantages and limitations, depending on the characteristics of the effluent to be treated. Table 1 summarizes the methods most employed for removing toxic inorganic species from contaminated water and industrial effluents, indicating their main advantages and disadvantages.

Methods such as chemical precipitation and coagulation/flocculation are the most employed; however, they often involve high operational costs (mainly with construction of tanks and their maintenance), significant consumption of chemicals (mainly with CaO and flocculants), and generation of secondary waste (toxic sludge) that needs to be disposed of safely. For low pollutant concentrations (<1 mg L^−1^), advanced techniques such as electrolytic recovery, membrane filtration, and reverse osmosis offer high efficiency but also require substantial investments in infrastructure and maintenance, along with strict operational control [19].

Although ion exchange resins based on non-renewable polymeric materials are also used as efficient adsorbents to remove trace-level inorganic pollutants, more sustainable alternatives have been developed to comply with the need for a fast transition to a bio-based circular economy, due to the increase in global warning. Among the possible alternative solutions, bioadsorption using raw and chemically modified agricultural lignocellulosic residues has emerged as a promising option, providing an efficient, economical, renewable, and environmentally friendly solution [20].

Bioadsorbents can be applied simply and efficiently, enabling the selective removal of inorganic pollutants, without requiring high capital investments or excessive use of chemicals. Recently, bioadsorption was tested in Ouro Preto, Brazil, in household filtration systems to treat water contaminated with arsenic [21]. The filters tested were low-cost, simple, easy to install, regenerable, and reusable, not requiring energy for operation, unlike reverse osmosis [22].

**Table 1:** Comparison of methods employed for the removal of inorganic pollutants from water.

Method	Material	Advantages	Disadvantages	Reference
Chemical precipitation	Precipitants (lime, alkalis, sulfides)	Low concentrations of toxic inorganic species in the treated effluent Treatment of high volumes of water Simple to implement and operate	High demand for chemicals Strict control of pH and temperature for correct operation Generation of toxic sludge to be disposed of safely Low efficiency for removal of trace-level inorganic species	[2,19,20,22]
Ion exchange	Ion exchange resins	Treatment of high volumes of water/effluent Allows medium filtration rates Simple to implement and operate	Requires chemicals for regeneration and reuse High cost for acquisition of resins Exhausted resins need to be disposed of safely	[2,19,20,22]
Membrane filtration	Membranes	Enables reuse of water/effluent Produces high-quality treated water Strict limits of pollutant concentrations in effluents can be met	Membrane fouling High investment in equipment High costs of membranes, maintenance, and operation	[2,19,20,22]
Coagulation/flocculation	CaO, Al_2_(SO_4_)_3_, aluminum polychloride, FeSO_4_, FeCl_3_	Treatment of high volumes of water/effluent Supports high flow rates Simple to implement and operate	High demand for chemicals Strict control of pH and temperature for correct operation Generation of toxic sludge to be disposed of safely Low efficiency for removal of trace-level inorganic species	[2,19,20,22]
Electrolytic recovery	Electrodes and electrolytes	Recovery of pure metal High selectivity for target compounds High metal removal rates	High demand for chemicals (electrolytes) High operating cost (electrodes) Reduced efficiency for very diluted pollutants High cost for large-scale implementation	[19,20]
Reverse osmosis	Membranes	Medium to high removal rates for toxic inorganic species Meets strict concentration limits for target pollutants	High investment in equipment High costs of membranes, maintenance, and operation Membrane fouling	[19,20]
Adsorption	Adsorbents	Highly efficient for low and high concentrations of inorganic pollutants Low cost for acquisition of adsorbents and operation Meets strict concentration limits for pollutants Simple to implement and operate	Requires chemicals for regeneration and reuse Exhausted adsorbents need to be disposed of safely	[19,20,22]

## 4. Adsorption

### 4.1. Adsorption Phenomenon

Adsorption is a phenomenon in which adsorbate species move into the interfacial layer between two bulk phases. One of these phases is necessarily a solid adsorbent surface, while the other can be a liquid or gaseous phase [23]. Once adsorbed, the chemical species tend to preferentially concentrate at the interface, rather than remaining in one of the two phases of the system. Hence, adsorption can be considered a mass transfer phenomenon, where the efficiency of adsorbents in adsorbing and concentrating certain substances on their surfaces allows the removal of chemical species from a liquid or gaseous phase [24].

**Figure 1:**
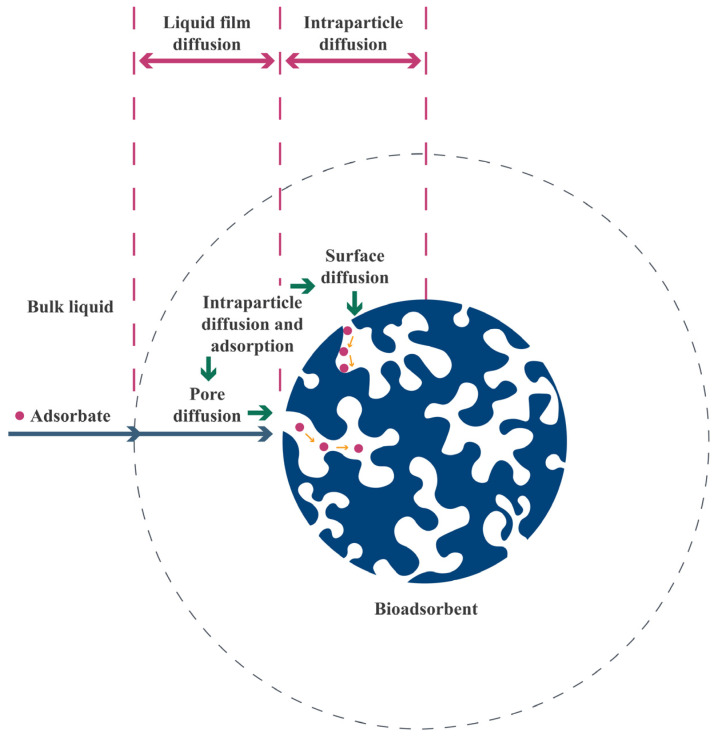
Main steps in solute mass transfer in the adsorption phenomenon. Adapted from Weber and Smith [25].

Adsorption mass transfer includes four migration steps (Figure 1). In the first step, the adsorbate species are transported from the fluid to the vicinity of the adsorbent particles, where there is a boundary layer consisting of a film of solvent molecules surrounding the adsorbent particles. In this step, mass transfer occurs by both convection and diffusion [25]. In the second step, there is transfer of the adsorbate species from the boundary layer to the adsorbent pore entrance, denoted external diffusion, which mainly involves diffusional mass transfer driven by the concentration gradient [25]. In the third step, transport of the adsorbate species within the pores, denoted internal diffusion or intraparticle diffusion, involves the diffusion of adsorbate species through the liquid contained within the pores [25]. There can also be surface diffusion of the adsorbate species along the surface of the adsorbent, as well as into the pores [26]. In the final step, there is the adsorption of adsorbate species onto available sites on the surfaces of the adsorbent particles [25].

### 4.2. Adsorption Mechanisms

**Figure 2:**
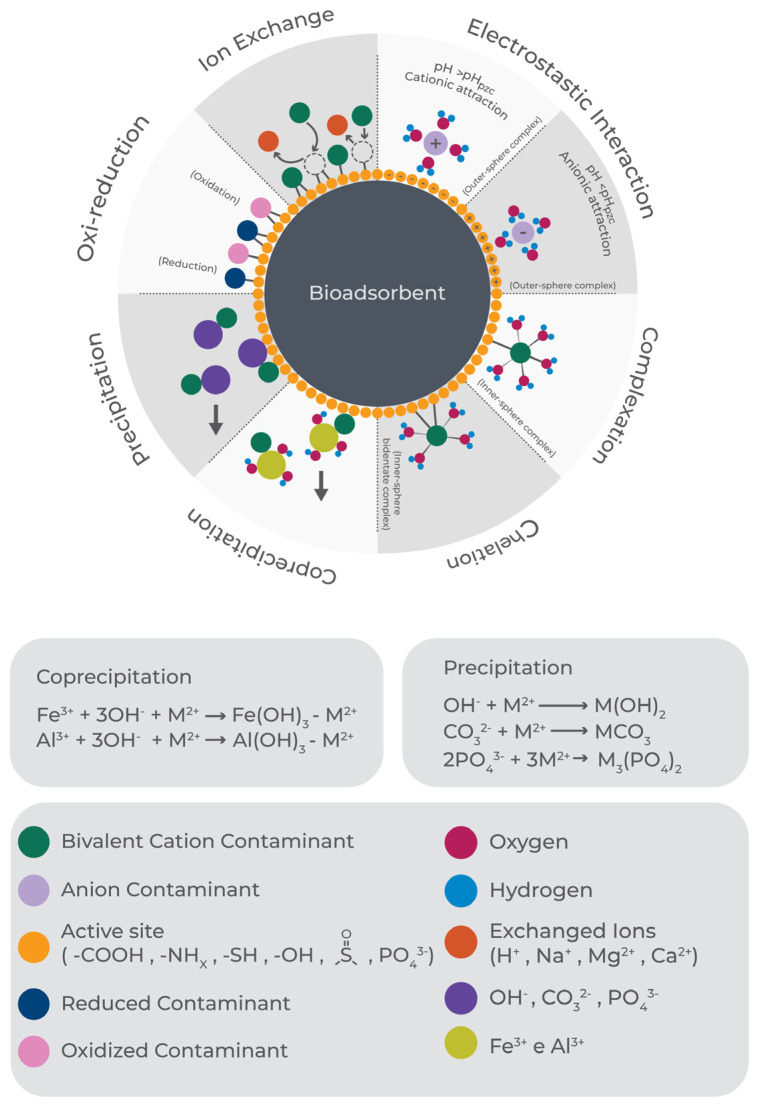
Most common types of interactions involved in the adsorption of inorganic contaminants on lignocellulosic-based adsorbents.

Lignocellulosic adsorbents have complex chemical characteristics, implying a diversity of pathways for the removal of contaminants from water [27]. Therefore, in-depth understanding of adsorption mechanisms is essential for increasing removal efficiency and selectivity towards target pollutants, while also assisting in the definition of a suitable regeneration method [28]. Depending on the type of lignocellulosic biomass and/or the type of chemical modification employed, there will be different proportions of functional groups capable of attracting ionic species to the surface of the solid, including amide, amine, carbonyl, hydroxyl, sulfonate, carboxylic, phenolic, phosphate, thioether, and phosphodiester groups, which play important and varying roles in the adsorption of target pollutants [29]. Physical adsorption, ion exchange, oxidation-reduction, surface complexation, chelation, and precipitation are among the most common types of interactions involved in the adsorption of inorganic contaminants on bioadsorbents (Figure 2) [29,30,31].

The exchange of ions from the adsorbent surface by ions from the aqueous solution is the most common adsorption mechanism found for adsorbents from renewable sources. It is usually reversible and occurs by the phenomenon of electrostatic attraction [31]. Mg(II), Ca(II), and Na(I) are among the most common ions released from the surface of lignocellulosic biomass during ion exchange, so their detection in solution is often used to identify the occurrence of this adsorption mechanism. Adsorption also enables the treatment of water and effluents by the oxidation or reduction of ions to less toxic forms, such as the reduction of Cr(VI) to Cr(III), or Cu(II) to Cu^0^ [27].

The complexation mechanism involves chemical interaction between ions in solution and ligands on the surface of the adsorbent, forming outer- or inner-sphere complexes (Figure 2), depending on the metal ion bonded to the surface functional group, with or without the intervention of water molecules. Functional groups attached to the surfaces of lignocellulosic materials may be mono- and polydentate ligands, where the latter are preferred, due to the possibility of simultaneous attraction of different types of ions. Furthermore, polydentate ligands form more stable chelates, compared to other complexes [28,31].

Recent studies focusing on the biosorption of metal ions have shown important contributions of hydroxyl, carboxyl, sulfonyl, and amine groups to the sorption capacity and selectivity of biosorbents for target pollutants, since these groups can coordinately bind to form chelates with metal ions [32]. In particular, amine groups are considered the most efficient in removal of both cationic and anionic species, because chelation (with the amine in its molecular form) and electrostatic (with the amine in its quaternary form) interactions can occur simultaneously, depending on the pH of the water [33]. If the concentration of metal ions on a solid surface increases above its adsorption capacity, depending on the solution pH, insoluble metal oxides, hydroxides, carbonates, or phosphates may accumulate on the surface of the biosorbent, implying the occurrence of a precipitation phenomenon, leading to a slower adsorption rate, compared to other mechanisms [29].

### 4.3. Batch and Continuous Adsorption

Batch, fixed-bed, and fluidized-bed are the main configuration modes discussed in the literature for decontamination of water and industrial effluents by adsorption. The batch mode is generally restricted to the treatment of smaller quantities of water, because its non-continuous operation hinders practical application [8]. In contrast, continuous mode using a column is more suitable for real applications involving the treatment of larger quantities of water, so this mode is widely used on an industrial scale [6,8,27,34]. Considering the two continuous operating modes, the fixed-bed column generally provides longer and more uniform operating times, compared to the fluidized-bed column. However, the fluid velocity in a fluidized-bed column is sufficiently fast for the bed to expand, resulting in faster adsorbent-adsorbate mixing and minimization of problems associated with fouling, plugging, and increased pressure drop during the treatment of water, especially in the case of industrial effluents [26]. On the other hand, the fixed-bed column is a simpler and cheaper arrangement, compared to the fluidized-bed column [35]. In the treatment of water and industrial effluents, the use of fluidized-bed reactors is indicated for the secondary treatment of water with high organic load, suspended solids, or potential biofilm formation [26].

Fixed-bed columns are frequently employed for tertiary treatment of water, since they are suitable for the removal of inert inorganic and organic contaminants from water containing little or no suspended solids [26]. In a fixed-bed column, a known amount of adsorbent is packed into the column, until reaching the desired height, and fixed into position using porous supports at the top and bottom. The porous supports avoid bed mobility and act as fluid dispersers, providing homogenization of the fluid entering the column and preventing formation of preferential channels [6]. The liquid phase may be fed into the column in either upward or downward flow, with the adsorbate concentrations in the solid and fluid phases varying as a function of time and position along the column [8]. Upward flow is most widely used, because it avoids bed compaction and the formation of preferential channels, although downward flow is sometimes preferred, since it can avoid fluidization of the particles and loss of fines, which may occur when low-density particles are fed at high rates [36,37].

Fixed-bed column adsorption does not operate within the equilibrium condition of batch adsorption, so data provided by the batch adsorption isotherm are not fully capable of describing the adsorption in a fixed-bed column [9]. To circumvent this, it is necessary to carry out tests to obtain the best operating parameters to optimize the process and achieve the highest adsorption capacity, the greatest efficiency of the adsorption process, and the most effective use of the bed [9]. To investigate the characteristics of adsorption columns, it is necessary to develop models that can accurately represent their behaviors. These models should be able to describe the dynamic behavior of the column, in terms of the inlet and outlet concentrations of the adsorbates, as a function of time (using breakthrough curves) [9]. In continuous mode operation, the adsorption mechanism is determined by phenomena including axial dispersion, film diffusion resistance, intraparticle diffusion resistance (both pore and surface diffusion), and adsorption equilibrium [10].

**Figure 3:**
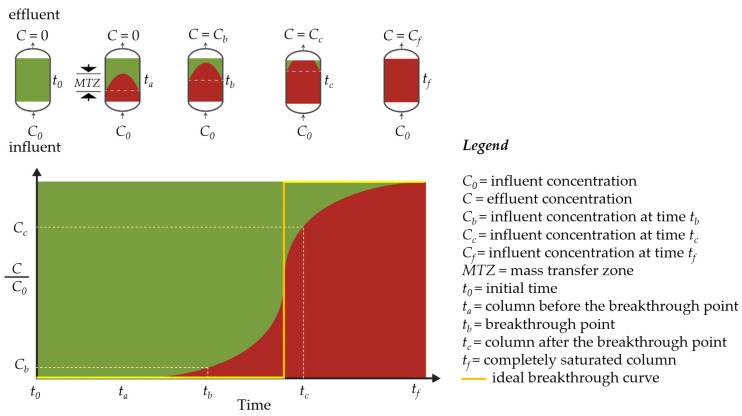
Scheme of the adsorption process for a monocomponent contaminant solution in a fixed-bed column, and breakthrough curve. Adapted from Thirunavukkarasu, Nithya, and Sivashankar [6].

Figure 3 shows the behavior of the mass transfer zone (MTZ) and the breakthrough curve, as a function of time, for a monocomponent adsorption process. At the beginning of the operation, the fresh column is fed with an influent with a known inlet concentration (*C*_0_). As the influent passes through the column, adsorption occurs, forming an adsorption region known as the MTZ [6], which is where the mass transfer rate is highest. In ideal adsorption systems (yellow solid line in Figure 3), where the mass transfer resistances are neglected, a linear frontline within the column results in a sudden increase in the concentration in the effluent when the bed becomes saturated. However, resistance to mass transfer occurs due to non-ideal behavior of the system (sigmoidal curve in Figure 3), including external and internal mass transfer resistances (kinetic effects) at the solid–liquid interface, together with thermodynamic equilibrium (thermodynamic effects), which act to increase the MTZ length [6,35]. In a non-ideal system, the MTZ for a monocomponent system may be represented by a sigmoidal curve [15,35] (Figure 3). When the outlet (*C*) to inlet (*C*_0_) adsorbate concentration ratio reaches a stipulated minimum value, the breakpoint occurs. Subsequently, the *C*/*C*_0_ ratio continues to increase until reaching *C*/*C*_0_ = 1, corresponding to the exhaustion point and saturation of the column [8]. The area above the curve of *C*/*C*_0_ plotted against time (Figure 3) reflects the amount of adsorbate retained by the bed [8].

Particularly for lignocellulosic bioadsorbents, the challenges associated with the resistance to mass transfer are directly related to the characteristics of the solids and their behavior in the column, such as specific gravity, granulometry, and particle length-to-diameter ratio, which can be the cause of pressure drops, formation of preferential channels, and non-uniform flows through the bed [6]. Other factors influencing the behaviors of these materials in continuous adsorption systems include the surface heterogeneity of lignocellulosic materials, as well as the presence of different functional groups (aliphatic hydroxyl, phenolic hydroxyl, carboxylic acid, ester, ether, ketone, methoxy, and aromatic rings), including those that can be introduced on the surface by chemical modification.

## 5. Fixed-Bed Column Adsorption Studies with Raw and Modified Lignocellulosic Residues

### 5.1. Overview

**Figure 4:**
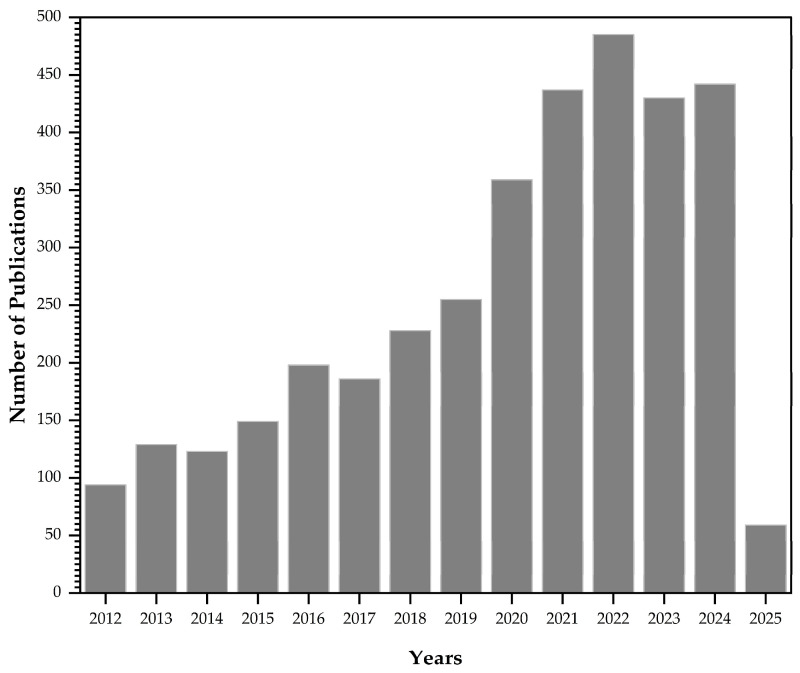
Number of articles published from 2012 to 2025 related to the application of biosorbents in fixed-bed adsorption. Database: Web of Science (Clarivate Analytics^®^, Boston, MA, USA).

In recent years, there has been increasing exploration of continuous adsorption using fixed-bed columns packed with raw and chemically modified lignocellulosic bioadsorbents (Figure 4), due to the need to find alternative treatment methods that are not only technically efficient and economically feasible, but also environmentally friendly and sustainable.

Table 2 presents adsorption data reported in some relevant studies over the last decade, selected according to the criteria shown in the Supplementary Materials, where different lignocellulosic materials were employed as bioadsorbents in fixed-bed columns. Table 2 also includes the types of lignocellulosic biomasses and modifying agents (MA) used for production of bioadsorbents; the contaminant ions removed; and the operational conditions, such as pH, bed height (*Z*), flow rate (*Q*), and influent concentration (*C*_0_). The equilibrium adsorption capacity (*Q*_e_), mathematical models applied to describe the adsorption, effective use of the bed (*Z*_e_), and performance parameters for regeneration of bioadsorbents are also presented in Table 2. The regeneration parameters include adsorption efficiency (*E*_ads_), desorption efficiency (*E*_des_), re-adsorption efficiency (*E*_re-ads_), number of cycles, and types of eluents used. All the equations used to estimate the parameters presented in Table 2 can be found in the Supplementary Materials. The data presented in Table 2 will be discussed throughout the review.

Evaluating Table 2, many lignocellulosic materials derived from agricultural residues have the potential to be used in the production of new bioadsorbents for the efficient removal of inorganic pollutants from aqueous media, including stalks [38,39,40], shells [41,42,43,44,45,46,47,48,49,50], *Moringa oleifera* seeds [51], legume husks [52,53,54], rice straw [55], leaves [37,56,57,58,59,60], sugarcane and palm bagasse [44,61,62,63,64,65,66,67,68,69,70,71,72,73], peels [44,56,70,74,75,76,77,78,79], and fibers [80,81,82] (Table 2). These materials can be highlighted for their ability to remove inorganic contaminants, with a wide adsorption capacity range (0.001–11.00 mmol g^−1^). Remarkable adsorption capacities were reported for Litchi peel [74], which was able to remove 10.99 mmol g^−1^ of Cr(VI) (Table 2), and for olive tree prunings treated with HNO_3_ [83], which could remove 2.89 mmol g^−1^ of Pb(II) (Table 2). The wide range of adsorption capacities can be explained by factors including the nature of the lignocellulosic biomass, the type of chemical modification employed, the toxic species removed, and the adsorption mechanism.

Low cost and high availability throughout the year are crucial for the feasibility of applying lignocellulosic materials as bioadsorbents. Biomasses that are non-perennial or with low availability can be problematic for commercial applications, even if they provide high performance [29,84]. A greater focus on the use of residues from agricultural crops such as rice [52,55], wheat [85], corn [38], and sugarcane [44,61,62,63,64,65,66,67,68,69,71,72,73] (Table 2) can assist in overcoming this difficulty, since these types of residues are abundant and widely available in many regions of the world.

**Table 2:** Adsorption data from the most relevant studies that applied lignocellulosic biomasses as adsorbents in fixed-bed columns in recent years (from 2012 to 2025).

Matrix	MA	pH	Ion	*C*_0_ /mg L^−1^	*Z* /cm	*Q* /mL min^−1^	*Q*_e_ /mg g^−1^	*Q*_e_ /mmol g^−1^	Model	Z_e_/%	Performance Parameters for Regeneration					Reference
											Number of Cycles	*E*_ads_/%	*E*_des_/%	*E*_re-ads_/%	Eluent	
Coconut leaves (*Cocos nucifera*)	-	1	Cr(VI)	100	20	4	4.88	0.98	Tho, YN, MDR	58.3	-	-	-	-	-	[86]
		2	V(V)	100	20	4	4.89	1.11		57.9						
Hazelnut shell (*Corylus* spp.)	H_3_PO_4_ Urea	6.27	Li	10	1.5	0.25	22.29	3.21	Tho, YN, MDR	81.20	5	-	100	-	H_2_SO_4_	[87]
Pine cone shell (*Pinus sylvestris*)	-	5.6	Cu(II)	120.9	20	2.65	11.1	0.17	Sips	-	-	-	-	-	HCl	[88]
			Pb(II)	96.7	0.7		55.57	0.27								
Fibers, coniferous trees (*Pinus* spp.)	H_3_PO_4_		Fe	1420	60 (4 columns)	1000	1635.0	29.27	-	-	-	-	-	-	-	[89]
			Mn(II)	18.3			23.1	0.42								
			Zn(II)	19.2			18.1	0.28								
Sugarcane bagasse (*Saccharum officinarum*)	BAD	5.75	Cd(II)	56.2	3.1	2.5	65	0.58	-	16	4	-	99.4–92.8	94.3–90.1	HNO_3_	[90]
		5.25	Pb(II)	207.2			153	0.71		15		-	100.5–98.7	108.3–91.2		
*Gundelia tournefortii*	EDTAD	6	Cu(II)	10	2	7	15.61	0.25	BA, Tho, YN	53.8						[91]
Ramie stalk (*Boehmeria nivea*)	H_3_PO_4_	5.5	Zn(II)	32.7	5	5	32.4	0.495	BA, Tho, YN, BDST	71	5	-	98.5–97.1	99–90	HCl	[92]
Cucumber peel (*Cucumis sativus*)	-	5	Cd(II)	50	8	20	300.26	2.67	Tho, YN, BDST	-	3	-	97–95	-	HCl	[93]
Walnut shell (*Juglans regia*)	ECH TEA DETA	3	Cr(VI)	200	3	10	308.4	5.76	Tho, Ck	-	2	-	-	83	NaOH	[94]
Palm bagasse *Opuntia ficus-indica*	-	2	Cr(VI)	100	-	45	111.45	2.1434	BA, Tho, YN, BDST	-	-	99–100	-	-	-	[70]
		6	Ni(II)				103.49	1.7633				87–100				
Yam peels (*Dioscorea alata*)		2	Cr(VI)				50.12	0.9641				65–100				
		6	Ni(II)				30.04	0.5118				87–99				
Plantain (*Musa paradisiaca*)	NaOH	2	Cr(VI)	100	11.4	45	18.25	0.351	-	-	-	-	-	-	-	[95]
		6	Ni(II)				22.08	0.376								
Yam (*Dioscorea alata*)	NaOH	2	Cr(VI)	100	9.0	45	28.01	0.539	-	-	-	-	-	-	-	
		6	Ni(II)				28.01	0.477								
*Moringa oleifera* seed	-	7.0	Cd(II)	0.3	32.1	2.0	0.065	5.82 × 10^−4^	Tho	80	-	-	-	-	-	[51]
Sugarcane bagasse (*Saccharum officinarum*)	-	4	Zr(IV)	75	3	1.8	20.32	0.223	Tho, BDST	59	-	15–26	-	-	-	[65]
Sugarcane bagasse (*Saccharum officinarum*)	H_3_PO_4_ NaNO_2_	5	Co(II)	1.26	3.8	1.4	13.20	0.224	BA	39	-	-	85	93	-	[67]
			Cu(II)	0.68			35.20	0.554		18		-	98	104		
Orange peel (*Citrus sinensis*)	-	2	Se(IV)	140	9	1	3.37	0.043	Tho, BDST	25	2	87	70	45	NaOH	[77]
Cariaquillo (*Lantana camara*)	H_2_SO_4_	1.5	Cr(VI)	300	4	4	362.8	6.98	Tho, BDST	9	3	53	-	30–71	NaOH	[96]
Almond shell (*Prunus dulcis*)	-	3.7	Cr(VI)	67.5	7	3	21.92	0.422	Tho, YN, MDR	-	-	66	-	-	-	[49]
			Cu(II)	7.0			2.39	0.038		-		70	-	-		
Cashew nut shell (*Anacardium occidentale*)	H_2_SO_4_		Cr(VI)	21.05	10	5	10.79	0.208	BA, YN, Ck	-	-	56	-	-	-	[50]
			Mg(II)	20.3			9.82	0.179		-		53	-	-		
Walnut shell (*Juglans regia*)	NaOH ECH DETA	3	Cr(VI)	30	2	8.0	28.1	0.540	YAN	6.5	3	49	18–21	51–72	NaOH	[45]
Sugarcane bagasse (*Saccharum officinarum*)	ECH TEPA	5	Cu(II)	20	-	6.25	16.51	0.26	-	38	-	52	>95	-	EDTA-2Na	[62]
			Cd(II)				11.24	0.10		33		75				
Sugarcane bagasse (*Saccharum officinarum*)	ECH TEPA	4.5	Cu(II)	12.5	-	6.25	10.8	0.170	YN	48	5	26	>95	-	EDTA-2Na	[73]
	H_3_PO_4_ Urea	4.5	Pb(II)	20.8	-	6.25	64.2	0.310	YN	19	5	80	>95	-		
Olive oil residues (*Olea europaea*)	-	6.0	Pb(II)	10	9.2	5	8.15	0.04	-	-	-	-	-	-	-	[97]
			Cd(II)				3.50	0.03								
			Ni(II)				2.90	0.05								
Breadfruit (*Artocarpus nobilis*)	-	-	Ni(II)	10.0	10	8.4	4.19	0.071	Tho, BA, YN	-	-	93	-	-	-	[98]
Sugarcane bagasse (*Saccharum officinarum*)	H_3_PO_4_ Urea	5	Pb(II)	103.6	-	6.25	76.5	0.369	BA, YN	-	-	-	90–99	-	EDTA-2Na	[71]
			Cu(II)	31.78			23.1	0.363								
			Zn(II)	32.69			21.7	0.332								
			Cd(II)	56.21			30.3	0.270								
			Ca(II)	20			17.9	0.447								
Sugarcane bagasse (*Saccharum officinarum*)	PMDA DMAc	5	Pb(II)	25	12.1	6.25	163.69	0.79	BA, modified BA	-	-	-	>99	-	EDTA-2Na	[64]
Waste tea (*Camellia sinensis*)	-	2	Cr(VI)	100	-	300	94.34	1.81	-	>80	4	30–90	10–20	>50	NaOH	[99]
Sugarcane bagasse (*Saccharum officinarum*)	TA SA	5.75	Co(II)	123.8	3.1	2.5	59.52	1.01	BA	23	5	-	93	100	HNO_3_	[61]
			Ni(II)	123.3			61.03	1.04		29		-	93	100		
Sugarcane bagasse (*Saccharum officinarum*)	CA	5	Pb(II)	99.87	7	1.5	158.9	0.767	Tho, YN	-	4	-	10–95	>80	HCl, HNO_3_, NaOH, NaCl	[63]
Guava leaves (*Psidium guajava*)	-	-	Cr(VI)	20	10	40	8.72	0.168	Tho, BA	77	-	0.168	-	-	-	[59]
Tea tree shell (*Melaleuca alternifolia*)	-	-	Cu(II)	28.82	-	7.4	7.42	0.117	Tho	<30	-	90–95	-	-	-	[46]
			Pb(II)	150.66		8.0	4.17	0.020								
			Cd(II)	80.59		7.4	18.02	0.160								
Eucalyptus leaf (*Eucalyptus globulus*)	-	3.8	Pb(II)	20.72	24	10	32.74	0.158	-	51	3	30–50	>80	>75	HNO_3_	[57]
			Cd(II)	11.24			7.24	0.064		48				>54		
			Ni(II)	5.87			1.77	0.030		33				>43		
Coconut coir (*Cocos nucifera*)	H_2_O_2_	6	Cd(II)	30	21	15	7.22	0.642	BDST	37	3	70–77	>94	>93	HCl	[100]
Pine bark (*Pinus pinaster*)	NaOH CS_2_	5	Cr(III)	500	2.5	10	53.04	0.78	Tho, BA, YN	47	1	79	53–68	-	H_2_SO_4_	[101]
Olive cake (*Olea europaea*)	NaOH	4.5–5	Pb(II)	200	-	4.0	13.21	0.064	Tho, YN, BDST	45	-	-	-	-	-	[102]
			Cu(II)				5.17	0.082		28						
			Cr(VI)				3.08	0.059		13						
			Zn(II)				5.51	0.843		21						
Lentil husk	-	5	Pb(II)	100	10	20	205.87	0.993	Tho, BA, YN	63	3	96	96–98	>95	HNO_3_	[53]
Fig leaf (*Ficus carica*)	-	5.5	Co(II)	5.4	2	1	11.09	0.188	Tho, BA, YN	22	-	50	-	-	-	[58]
			Pb(II)				12.27	0.059		24						
Pine nuts shell (*Pinus pinea*)	-	5	Cu(II)	100	3.80	6	7.14	0.112	Tho, YN, BDST		-	19–99	-	-	-	[42]
			Pb(II)				20.25	0.098				51–100				
Sugarcane bagasse (*Saccharum officinarum*)	-	5	Cd(II)	10	28	1.6	0.146	1.30 × 10^−3^	YN, Tho, BDST	44	-	91	-	-	-	[69]
			Pb(II)				0.154	7.43 × 10^−4^		55		90				
Ramie stalk (*Boehmeria nivea*)	EPH NaOH Na_2_CO_3_ TEPA	5	Cu(II)	44.49	10	5	34.0	0.535	BA, Tho, YN, BDST	81	5	82	97–98	100	EDTA-2Na	[32]
Apple pomace (*Malus domestica Borkh*)	H_2_SO_4_ CS_2_	4	Pb(II)	50	7	60	192	0.927	Tho	53	5	18–70	75–100	>80	HNO_3_	[103]
Mercerizing pine bark (*Pinus pinaster*)	NaOH	5	Cr(III)	100	7.5	10	35.35	0.680	Mech, BA, Tho, YN	53	1	50	47	-	HNO_3_	[104]
Sugarcane bagasse (*Saccharum officinarum*)	TA	5.5	Cu(II)	60.37	3.2	1.7	67.36	1.06	BA, Tho	58	3	60	>95	>92	HNO_3_	[105]
			Co(II)	64.83			44.79	0.76		61		62				
			Ni(II)	204.84			57.52	0.98		50		55				
Sugarcane bagasse (*Saccharum officinarum*)	PMDA	5	Pb(II)	80	-	6.25	258	1.25	YN, modified YN	56	-	>99	98	-	HNO_3_	[68]
Pongamia oil cake (*Millettia pinnata*)	-	4.5	Zn(II)	100	14.8	5.6	85.98	1.315	Tho, BDST	35	6	86	-	-	EDTA	[106]
Raw watermelon rind (*Citrullus lanatus*)	H_3_PO_4_	6	Ni(II)	75	5	5	33.12	0.564	BA, Tho, YN	1	-	44	80	-	HCl	[107]
Tarap leaves (*Corchorus capsularis*)	NaOH EDTA	5	Pb(II)	100	10	8.33	109.70	0.529	-	77	4	85	20–95	100	Acid, base, water	[108]
Jute fiber (*Corchorus capsularis*)	PMDA	6	Cu(II)	100	12	54.78	40.05	0.630	Tho, YN, BDST	34	-	59	-	-	-	[80]
Sugarcane bagasse (*Saccharum officinarum*)	-	2	Cr(VI)	15	20	2	1.99	0.038	-	12	-	98	-	-	-	[66]
		5	Ni(II)				3.43	0.058		39		96				
Olive oil residues (*Olea europaea*)	-	6	Pb(II)	10	-	11.67	5.33	0.026	-	34	-	-	-	-	-	[109]
			Cd(II)				2.71	0.024		14						
			Ni(II)				1.98	0.034		10						
Calabasa (*Cucurbita moschata*)	Na_2_CO_3_	5	Cu(II)	50	-	1.0	5.0	0.08	-	-	-	10	87.82	-	HCl	[110]
			Ni(II)				6.0	0.10				12	81.57			
Olive stone (*Olea europaea*)	-	5	Cu(II)	100	13.4	6.0	5.06	0.080	BA, Tho, YN, BDST, ANFIS	-	-	20–100	-	-	-	[43]
Pine shell (*Pinus pinea*)							7.14	0.112				10–81				
Grapefruit peel (*Vitis vinifera*)	CA	5	Cu(II)	266	16	1.3	48.54	0.764	BA, Tho, YN, BDST	0	-	-	-	-	-	[78]
Tea waste (*Camellia sinensis*); mapleleaves (*Acer pseudoplatanus*; mandarin peel (*Citrus reticulata*)	NaOH CaCl_2_	5.5	Cd(II)	20	31	10	38.25	0.340	Tho, BDST	61	3	47–50	22–49	>99	HCl	[56]
			Cu(II)				63.37	0.997		61		48–51	23–48			
			Pb(II)				108.12	0.522		61		56–57	34–58			
			Zn(II)				35.23	0.539		60		52–54	23–46			
Cucumber peel (*Cucumis sativus*)	-	5	Cd(II)	100	8	20	180.53	1.61	BA, Tho, YN	36	3	77–85	94–98	-	HCl	[75]
Sugar beet pulp (*Beta vulgaris*)	-	5	Zn(II)	500	66	50	5.23	0.08	BA	35	3	-	-	-	H_2_SO_4_, HCl, HNO_3_, EDTA-2Na	[111]
Pine cone shell (*Pinus halepensis*)	-	5	Pb(II)	100	14.3	2	8.09	0.175	-	0	4	92	14–64	83	HCl	[47]
			Cu(II)				36.19	0.127		0		23	19–63	100		
Litchi peels (*Litchi chinensis*)	-	3	Cr(VI)	50	5	1.5	41.2	0.65	BDST	40	3	-	>99	>94	HNO_3_	[74]
Maple tree leaves (*Acer pseudoplatanus*)	-	5	Cu(II)	55.0	21	21	18.3	0.288	SREQ	21	8	53	93–99	100	H_2_SO_4_	[37]
Java jute (*Hibiscus cannabinus*)	NaClO NaOH HCl	7	Cr(VI)	0.5	15	2.2	0.021	4.04 × 10^−4^	YN, Tho, BA	25	-	52	88–92	78–98	NaOH, HCl	[112]
Spent coffee grounds (*Coffea*)	-	4.5	Cd(II)	11.41	14	5.5	13.49	0.12	-	13	4	<50	60–100	>99	HNO_3_, CaCl_2_, AC	[113]
			Cu(II)	6.35			13.34	0.21		21						
			Pb(II)	20.72			66.30	0.32		20						
Grapefruit peel (*Vitis vinifera*)	H_2_O_2_	5.5	Cr(VI)	35	150	-	39.06	0.615	HSDM (simulation)	50	-	-	27–73	>95	-	[79]
Watermelon rind (*Citrullus lanatus*)	-	-	Pb(II)	Pb(II)	5	1	55.00	0.265	BA, Tho, YN	-	3	0.265	-	-	HCl	[114]
Rice husk (*Oryza sativa*)	-	8.0	As(V)	0.070	28	7.0	0.417	5.57 × 10^−3^	BA, Tho, YN	-	-	60–95	-	-	-	[52]
Olive tree pruning (*Olea europaea*)	-	4	Pb(II)	150	5	6	5.38	0.026	-	75	5	40	-	-	HCl	[83]
	H_2_SO_4_						6.48	0.313		76		50	-	-		
	HNO_3_						31.56	0.152		65		45	-	-		
	NaOH						34.42	0.166		57		53	60–99	>51		
Sesame	-	5.5	Cd(II)	60	2.0	2.5	22.88	0.204	-	56	4	74	72–94	>55	HNO_3_	[115]
Tururi fibers (*Manicaria saccifera* Gaertn.)	-	5.5	Cu(II)	276.72	9.5	4	104.03	1.64	HSDM	16–50	-	>90	-	-	-	[82]
			Cd(II)	299.53			126.75	1.13								
			Ni(II)	306.53			48.83	0.832								
			Pb(II)	303.7			52.59	0.254								
Grapefruit peel (*Citrus maxima*)	-	6	Pb(II)	300	3	2.5	160	0.772	Tho	25–56	-	-	-	-	-	[44]
			Cd(II)				132	1.17								
			Cu(II)				84	1.32								
			Ni(II)				60.7	1.03								
Passion fruit shell (*Passiflora edulis*)	-	6	Pb(II)	300	3	2.5	98.4	0.475								
			Cd(II)				48.6	0.432								
			Cu(II)				40	0.629								
			Ni(II)				25.8	0.440								
Sugarcane bagasse (*Saccharum officinarum*)	-	6	Pb(II)	300	3	2.5	49.8	0.240								
			Cd(II)				26.3	0.234								
			Cu(II)				23	0.362								
			Ni(II)				16.1	0.274								
Sunflower biomass (*Helianthus annuus*)	-	6.5	Co(II)	40	5	8	11.68	0.198	-	>90	-	-	-	-	-	[116]
Citrus peels (*Vitis vinifera*)	HNO_3_	4.0	Pb(II)	10.36	24	9	85	0.410	Tho	42	-	30–100	74–100	-	HNO_3_	[76]
			Cd(II)	5.62			44	0.391		31						
			Zn(II)	3.27			20	0.306		30						
Green coconut shells (*Cocos nucifera*)	NaOH	5.5–5.7	Cu(II)	200	100	200	47.41	0.746	Tho, EBCT	30–60	-	50–99	-	-	HNO_3_	[48]
			Ni(II)				26.53	0.452								
			Zn(II)				25.76	0.394								
Waste phoenix tree leaf (*Phoenix dactylifera*)	PMDA	6	Cu(II)	50	-	4.5	34.5	0.543	YN, modified YN, Wol	40–60	-	40–70	-	-	-	[60]
			Cd(II)				38.6	0.343								
			Zn(II)				28.5	0.436								
Coconut husk (*Cocos nucifera*)	-	7	Cu(II)	10	20	10	7.25	0.114	BDST, YN, Ck	45	-	52	-	-	-	[41]
Grape stalks (*Vitis vinifera*)	-	5.2	Cu(II)	12.71	6.7	0.5	11.69	0.184	HSDM	40–75	-	-	-	-	-	[39]
			Cd(II)	22.48			26.87	0.239								
			Ni(II)	11.74			13.81	0.228								
			Pb(II)	41.44			54.29	0.262								
Green bean husk (*Phaseolus vulgaris*)	-	4	Sb(III)	5	50	5.0	20.6	0.1692	Tho	20–30	7	>99	>97	>90	HCl	[54]
Hemp fiber (*Cannabis sativa*)	-	5	Co(II)	50	7	-	15.44	0.2620	Tho, YN	90	-	40–83	-	-	-	[81]
Rice straw (*Oryza sativa*)	NaOH	-	Ni(II)	75	2	0.5	43	0.7327	Tho, BA, YN	-	-	-	-	-	-	[55]
Residue of allspice (*Pimenta dioica*)	-	5.75	Pb(II)	15	15	20	16.2	0.0782	Tho, BDST	81	-	>99	-	-	-	[117]
Oil cake (*Brassica juncea*)	-	8	Ni(II)	10	-	1	9.5	0.1619	-	5	7	69	>99	>99	HCl	[118]
Olive pit (*Olea europaea*)	H_2_SO_4_	5	Pb(II)	150	4.4	6	17.7	0.0854	-	75	14	49	86–100	>80	HCl	[119]
Sugarcane bagasse (*Saccharum officinarum*)	ECH TEPA	5	Cu(II)	20	-	6.25	18.43	0.29	BA, YN	42	-	61	-	-	EDTA-2Na	[120]
Almond shell (*Prunus amygdalus*)	-	5	Cu(II)	100	4.4	6	32.158	0.155	-	-	-	29.29	-	-	-	[121]
			Pb(II)				10.531	0.166				82.27				
Corn stalk (*Zea mays*)	EPH DETA TEA	7.2	Cr(VI)	200	1.4	5	250	4.81	BA, Tho, YN	60–80	-	70–94	-	-	-	[38]

MA: modifying agent, *C*_0_: initial adsorbate concentration, *Z*: distance along column length, *Z*_e_: effective use of the bed, *Q*: volumetric flow rate, *Q*_e_: equilibrium adsorption capacity, *E*_ads_: efficiency of adsorption, *E*_des_: efficiency of desorption, *E*_re-ads_: efficiency of re-adsorption, BA: Bohart–Adams, YN: Yoon–Nelson, Wol: Wolborska, Tho: Thomas, BDST: Bed Depth Service Time, EBCT: Empty-Bed Contact Time, Ck: Clark, HSDM: homogeneous surface diffusion model, MDR: modified dose–response, ANFIS: Artificial Neural-Fuzzy Inference System, ECH: epichlorohydrin, TEPA: tetraethylenepentamine, DETA: diethylenetriamine, TEA: triethylamine, EDTAD: ethylenediaminetetraacetic dianhydride, BAD: butane-1,2,3,4-tetracarboxylic acid dianhydride, TA: trimellitic anhydride, SA: succinic anhydride, CA: citric acid, PMDA: pyromellitic dianhydride.

Among inorganic contaminants, the adsorption of divalent cations such as Cu(II), Pb(II), Ni(II), Co(II), Cd(II), Zn(II), Mn(II), and Hg(II) has been most extensively investigated (88%) (Table 2), mainly because these are the cations most commonly detected in water bodies and industrial effluents. Raw *Citrus maxima* peel (CM), passion fruit shell (PF), and sugarcane bagasse (SB) were used as bioadsorbents for the removal of Pb(II), Cd(II), Cu(II), and Ni(II) in monocomponent aqueous systems [44]. The best performance was shown by CM, followed by PF and SB (Table 2). PF had an advantage over SB, because it only required a simple pretreatment to avoid secondary pollution, without the need to use Soxhlet extraction with organic solvents, which was required for the removal of SB extractives [44].

To a lesser extent, there have been studies concerning the use of bioadsorbents for the removal from water of anionic species such as arsenic [52], chromium [38,45,49,59,70,79,86,94,96,101,104,112,122,123], and selenium [77] (Table 2). The smaller number of works can mainly be attributed to the low ability of raw lignocellulosic bioadsorbents to remove anionic species, due to the absence of a sufficient number of groups capable of interacting with anionic species, except for protein-rich lignocellulosic biomass containing high levels of amine and sulfur. Chemical modification with inorganic (mainly acids and bases) and organic (mainly ligands containing nitrogen, oxygen, and/or sulfur atoms) reactants is one of the techniques used to enhance the performance of lignocellulosic bioadsorbents in removing cations and anions.

### 5.2. Chemically Modified Lignocellulosic Adsorbents

**Figure 5:**
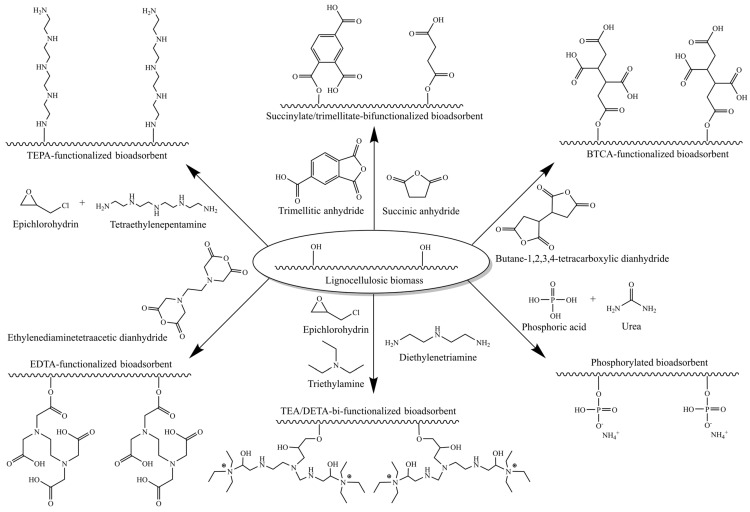
Some pathways used to prepare versatile fixed-bed adsorbers via chemical modification.

Figure 5 presents some chemical modifications directly performed on lignocellulosic biomass (without chemical pretreatment). As shown in Table 2, several studies have conducted treatments with acids, such as H_2_SO_4_ [50,96,119], HNO_3_ [76], and HCl [83], and with bases, especially NaOH [48,55,56,83,95,102,104,112].

The treatments with acids or bases can lead to improvements in the performance of lignocellulosic bioadsorbents. Acids such as phosphoric acid can be used to functionalize the surface of lignocellulosic materials with phosphate groups [73]. Acids such as H_2_SO_4_, HNO_3_, and HCl protonate the functional groups of lignocellulosic biomass, increasing the swelling capacity of the fibers in the structure of material and making it more selective for certain contaminant ions, by increasing their access to adsorption sites [119]. Treatment with NaOH can also increase the swelling capacity of fibers and the porosity of lignocellulosic materials, by solubilization of biomass components (such as hemicelluloses, protolignin, and extractives, among others), making the hydroxyl groups more available for chemical modification and/or increasing the adsorption capacity of the material by hydrolyzing ester bonds, leading to the formation of carboxylate groups and phenolate groups [48]. In some applications of lignocellulosic bioadsorbents, colored soluble compounds that cause secondary pollution are released into the water, highlighting the importance of performing extraction with organic solvents or using soft pretreatments with very diluted acids or bases, and even using diluted H_2_O_2_ to oxidize some compounds and make them more soluble and easier to remove from lignocellulosic biomass in the pretreatment step [79].

A multi-metal binding bioadsorbent (MMBB) was developed by combining tea waste, maple leaves, and mandarin peel, in a ratio of 3:2:1 (w w^−1^), followed by chemical modification using alkaline hydrolysis [56]. Under the best removal conditions (Table 2), the removal capacities for Cu(II), Cd(II), Pb(II), and Zn(II) were 1.00, 0.34, 0.52, and 0.54 mmol g^−1^ (63.54, 32.22, 107.7, and 34.31 mg g^−1^), respectively [52]. The column packed with MMBB, with a height of 31 cm, exhibited similar removal efficiencies for both real and semi-simulated effluent, achieving an effective use of the bed exceeding 60% [56] (Table 2).

The chemical modification routes of lignocellulosic materials are based on strategies involving the modification of hydroxyl groups, predominantly hydroxyl groups of AGU in cellulose, but can also be extended to hydroxyl groups and other functional groups in lignin and hemicelluloses. The chemical modification of hydroxyl groups with cyclic carboxylic acid anhydrides (Figure 5) is one of the most used strategies, as it introduces carboxylic acid functionality in lignocellulosic biomass. In this type of reaction, hydroxyl groups act as nucleophiles that promote esterification with one or more carbonyl groups (electrophiles) of the cyclic carboxylic acid anhydride, forming ester bonds and releasing carboxylic acid groups on the surfaces of materials [124].

On the other hand, for the introduction of amine groups, the most common approach is the use of intermediate molecules, such as epichlorohydrin, which serve as anchors for the new amine groups (when primary and secondary amines are used) or ammonium groups (when tertiary amines are used) [124]. To introduce phosphate groups, a reaction is carried out using H_3_PO_4_ in the presence of urea, which acts as a catalyst [73,87,107] (Figure 5). Finally, a less common method is the introduction of xanthate groups, by reaction of hydroxyl groups with CS_2_ in the presence of a strong base, such as NaOH (Table 2) [101].

Among the issues that must be considered for the technical-economic and environmental feasibility of chemical modifications of lignocellulosic materials is the reaction yield. Low reaction yields may result in high costs with solvents and reactants, requiring recovery operations. Another critical point is that reactants that were not consumed or that generated byproducts in the reaction medium after synthesis can become an environmental liability. The degree of hazard associated with the solvents and reactants should also be considered, which may impose higher operational safety costs. Therefore, it is highly recommended that both solvents and reactants should be obtained from renewable sources and be nontoxic and biodegradable, in accordance with the concept of a bio-based circular economy.

Under similar column operating conditions, raw sugarcane bagasse [69] and sugarcane bagasse modified with phosphoric acid [73] were used for Pb(II) removal. The presence of the phosphate group resulted in an adsorption capacity of 64.2 mg g^−1^ (0.310 mmol g^−1^) [73], which was 400 times higher than obtained using raw sugarcane bagasse (0.154 mg g^−1^ or 7.43 × 10^−3^ mmol g^−1^) [73] (Table 2).

Therefore, chemical modification of lignocellulosic materials has been used to obtain higher adsorption capacities and/or enhanced adsorptive properties, such as superior performance over a wide pH range, reduced swelling capacity, and increased surface area and porosity [29,84].

#### 5.2.1. Bifunctional Adsorbents for Simultaneous Cation and Anion Removal

The development of bioadsorbents capable of efficient simultaneous adsorption of both cations and anions is a more recent trend [125]. In this review, only a small number of studies were found that used lignocellulosic bioadsorbents to remove inorganic cations and anions, either separately or together in more complex systems [49,66,70,102] (Table 2).

For the same column operating conditions (pH 5, *C*_0_ = 100 mg L^−1^, *Q* = 45 mL min^−1^), Villabona-Ortíz et al. [70] obtained greater removal of Cr(VI) (2.14 mmol g^−1^) and Ni(II) (1.76 mmol g^−1^) using oil palm bagasse, compared to the use of yam peels (Cr(VI): 0.96 mmol g^−1^; Ni(II): 0.51 mmol g^−1^) (Table 2). These differences in the removal capacities of the biomaterials could be mainly explained by the different compositions of their structures, in terms of cellulose (including degree of oxidation of anomeric carbon, degree of polymerization, and crystallinity), hemicelluloses (including the presence of carboxylic groups in the oxidized sugar units, such as D-glucuronic and D-galacturonic acids), and lignin (including the type of lignin, such as guaiacyl-syringyl (GS) and guaiacyl-syringyl-*p*-hydroxyphenyl (GSH), and the presence of carboxyl, carbonyl, methoxyl, phenolic, and alcoholic hydroxyl groups). Additional factors affecting the removal capacity include the presence of minor components, such as proteins, and the role of each type of functional group in the adsorption of metal ions [124].

In general, raw lignocellulosic materials do not provide efficient removal of anionic inorganic contaminants from water. To overcome this limitation, chemical modifications can be performed to introduce functional groups with greater affinity for these species, such as quaternary amines [124].

Dovi et al. [94] and Chen et al. [38] developed bioadsorbents containing quaternary ammonium groups, using walnut shells (ACWNS) and corn stalks (MCS), respectively. The incorporation of these new functional groups in the biomass structure enhanced Cr(VI) removal, with ACWNS and MCS achieving adsorption capacities of 308.4 mg g^−1^ (5.78 mmol g^−1^) and 250 mg g^−1^ (4.81 mmol g^−1^), respectively (Table 2). These adsorption capacities are higher than those obtained using non-chemically modified biomass-based adsorbents, as shown in Table 2.

The introduction of tertiary, secondary, and primary amines increases the versatility of the materials for the removal of cations [32,62,73,120] and anions [45], although these chemical groups exhibit a strong dependence on the pH of the medium. Consequently, the removal of anions is only possible when the amines are in the protonated form. On the other hand, the removal of cations is only possible when the amines are in the molecular form.

Simultaneous removal of cationic and anionic species is not possible over a wide pH range, mainly due to the net surface charge of the bioadsorbent, as well as the charges and solubilities of the species in solution. Depending on the pH of the solution and the concentrations of the chemical species (cations, anions, or both) in solution, precipitate formation may occur.

Therefore, it is necessary to determine whether the removal of inorganic contaminants could occur by precipitation, rather than by adsorption. The pH of the solution is one of the most important operating parameters in bioadsorption processes, because it significantly affects the surface charges of the bioadsorbent and the adsorbate, consequently determining the affinity of the surface sites of the bioadsorbent for the adsorbed species.

#### 5.2.2. pH of the Point of Zero Charge (pH_pzc_)

Performance evaluation of bioadsorbents requires knowledge of the pH of the point of zero charge (pH_pzc_), which is the pH at which the surface of the bioadsorbent has zero net charge [22,126]. When the pH is below the pH_pzc_, the surface is predominantly positively charged, while above the pH_pzc_, the charge is predominantly negative [126,127]. The pH_pzc_ is crucial for understanding the behavior of the biomaterial during adsorption, since electrostatic attraction is favored when the surface charge is opposite to that of the ionic species to be adsorbed. However, this does not exclude the possibility of adsorption according to other types of mechanisms.

Chemical modifications strongly influence the pH_pzc_. Wang et al. [32] chemically modified ramie stalk with epichlorohydrin and tetraethylenepentamine, resulting in an amino-functionalized bioadsorbent, where the presence of amine groups in the biomass structure acted to change the pH_pzc_ from 2.0 to 9.4 [32]. The optimum condition for removing Cu(II) ions was at pH 5.0 (*Q*_e_ = 34.0 mg g^−1^) (Table 2). At this pH, the net surface charge of the bioadsorbent was positive, possibly causing electrostatic repulsion between the bioadsorbent and the adsorbate. Therefore, Wang et al. [32] proposed that the removal mechanism involved not only electrostatic attraction, but also complexation by coordination of amine groups with Cu(II) ions. Ideally, an adsorbent should provide good removal performance over a wide pH range, avoiding the need for pH correction in the water or effluent treatment unit, which would increase the cost of the chemicals used in the process.

### 5.3. Design and Optimization of Fixed-Bed Columns

**Figure 6:**
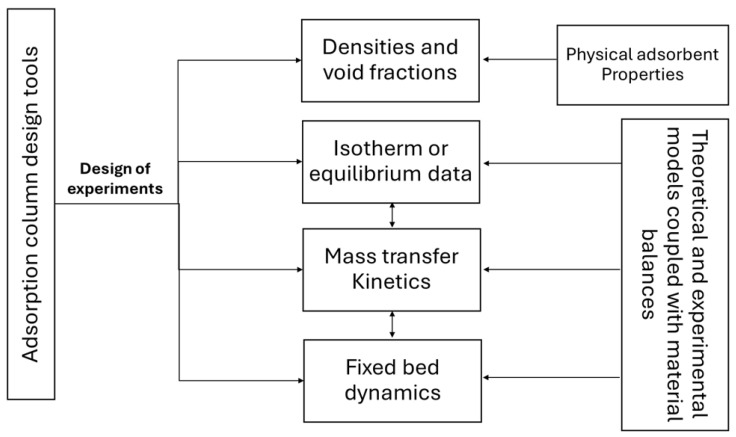
Conceptual flowchart of tools for designing fixed-bed adsorbers.

There are two distinct approaches to designing fixed beds. The first approach involves a thorough solution of conservation, transport, and thermodynamic equations. The second approach uses simplified techniques based on experimental data at laboratory, pilot, or industrial scales (Supplementary Materials). The rigorous method may require experimental work, if the necessary data are not available in the existing literature (as in the case of new lignocellulosic adsorbents). A conceptual flowchart of tools for designing adsorber columns is shown in Figure 6. Material balances, in integral or differential form, are essential relationships for describing adsorption on fixed-bed adsorbers and can include or neglect adsorption kinetics. Batch studies are important for obtaining data to enable prediction of the operating behavior in fixed-bed columns. Batch adsorption studies are performed considering the effects of parameters such as solution pH, contact time (kinetics), and initial contaminant concentration (isotherm), followed by application of the optimum conditions identified in column tests [8]. In continuous water treatment using a fixed-bed column, the adsorbate concentrations in the liquid and solid phases change according to time and column position [6]. The performance of the column is evaluated in experiments carried out under different operating conditions, where the main variables considered are the distance along column length (*Z*), the initial contaminant concentration (*C*_0_), and the flow rate (*Q*). Table 2 shows these parameters for operating conditions under which the best removal capacity (*Q*_e_) was obtained. Although the adsorption capacity is a useful parameter for measuring the efficiency of a bioadsorbent, there are other important criteria that need to be considered in optimization of column operation, such as the mass transfer zone and the effective use of the bed.

The mass transfer zone (*MTZ*, cm) of a column can also be related to the length of the unused bed height (*H*_unb_, cm), or expressed as a percentage (*H*_unb_/% = *H*_unb_/*H*_b_), according to the following equation:

(2) $$H_{unb}/\%=\left[\left(\frac{MTZ}{H_{b}}\right)\times \left(1-\frac{t_{b}}{t_{s}}\right)\right]\times 100$$

where *H*_b_ is the column bed height (cm), *t*_b_ is the breakthrough time (min), and *t*_s_ is the saturation time (min).

It is important to define the breakthrough time considering the permissible contaminant concentration according to the limit set by local environmental legislation, or other process conditions [66]. In general, the publications compiled in this review adopted the commonly used definition of *t*_b_ as the time when the outlet concentration (*C*) was equal to 0.05 *C*_0_ [8].

Values reported for the effective use of the bed (*Z*_e_/% = 100% − *H*_unb_) are presented in Table 2. Many of the *Z*_e_ values were calculated indirectly by visual analysis of the breakthrough curves, considering *t*_b_ (*C*/*C*_0_ = 0.05) and *t*_s_ (*C*/*C*_0_ = 0.95). Small *H*_unb_ values mean that the breakthrough curve is close to ideal, with negligible mass transfer resistance. The breakthrough time and saturation time are directly proportional to distance along column length (*Z*), while the initial contaminant concentration (*C*_0_) and flow rate (*Q*) are inversely proportional [128]. It can be seen that the *Z*_e_ values were on average low (~40%) (Table 2). The granulometric size of the biomaterial is an essential parameter to consider for column packing, operation, and maintenance, avoiding operational failures such as the appearance of preferential channels [4].

Another factor that must be considered when using lignocellulosic materials is their capacity for swelling by the absorption of water [29]. For these reasons, operation in upward flow mode is preferable, because it enables a uniform solute distribution and reduces pressure gradients and the potential for clogging of the column [26,36].

Furthermore, if the contaminated solution is fed using an upward flow, it is possible to use bioadsorbent particles with smaller diameters, consequently reducing the quantity of bioadsorbent required and leading to higher adsorption rates [129]. In addition to the adsorption capacity of the column, it is important that adsorption data such as the effective use of the bed and the mass transfer zone are properly obtained and evaluated, enabling optimization of the column treatment operation and the provision of sufficient information for scale-up of the technology, envisaging potential real applications.

### 5.4. Scale-Up and Technology Maturity of Lignocellulosic Adsorbent Columns

Several factors must be considered when designing small-scale column experiments to collect data for the design of a full-scale plant. Particular attention must be paid to the selection of length, diameter, and flow rate in small-scale experiments, as these variables can influence the hydrodynamics and dispersion characteristics, and, consequently, the correct upscaling.

The methods used for scale-up are typically based on one of the following principles [130]: (i) estimation of characteristic parameters of the MTZ and its application for full-scale adsorber design and (ii) application of an experimental setup that guarantees the similarity of mass transfer conditions between small- and large-scale adsorbers, in order to determine a normalized breakthrough curve (scale-independent). Therefore, optimal scale-up methods are fundamental to minimize technical, economic, and environmental risks. Table 3 shows the column dimensions and characteristics of the beds reported in works published since 2012 that used raw and chemically modified lignocellulosic residues in fixed-bed columns.

As with any technological development, adsorption using lignocellulosic adsorbents needs to be mature to the point at which it can be validated as a new product/process. A common measure of technology maturity is the TRL, which evaluates the maturity of technologies on a scale from TRL 1, corresponding to conception of the idea, to TRL 9, when the product reaches the industrial scale and is available on the market [131].

The experimental apparatus used in bench-scale adsorption tests has typically consisted of small columns with internal diameters of 1–5 cm and heights of 10–60 cm, which were employed in ~90% of the studies covered in this review (Table 3). In terms of maturity level, these new biomaterials could be classified at TRL ≤ 4, where there are laboratory-scale experimental tests for validation of the application of the prototype material [125,131]. Issues that were less studied (in ~7% of the studies compiled in this review), due to their greater complexity for academic researchers, were operational costs (including consideration of longer column heights, larger column diameters, and larger amounts of bioadsorbent) and advanced data analysis, including complex mathematical modeling using nonlinear models (Table 3). In this case, the adsorption tests were at maturity level 4 < TRL ≤ 6, with few applications under real and simulated pilot-scale conditions [41,48,79,89,99,111]. These data indicated the need, in bench-scale studies, for researchers to select columns with diameters and heights proportional to those that would be employed in large-scale operations. Only one study included in this review used more than one column [89], so there were columns in series, but without circular permutations, which would not allow the bioadsorbent to be saturated (increasing the effective use of the bed), without losing toxic and/or valuable metals, if, for economic reasons, the process was designed to recover the contaminant. Furthermore, it is necessary to consider technical and economic aspects.

Once the operating conditions of the bench-scale column have been optimized, the proportionality and scalability of these operating conditions, in relation to large-scale operation, can avoid undesirable variations in the maximum adsorption capacity, the efficiency of the adsorption process, and the effective use of the bed, which could make the process technically and economically unfeasible [99,111]. In this way, an advance of the TRL from 4 (bench-scale) to ≥6 (pilot-scale) would be technically feasible, allowing technology prototyping to develop a business model and, consequently, preparation for transfer of the technology to the market [93].

Raulino et al. [48] developed a pilot-scale experimental apparatus composed of an adsorption column with internal diameter of 62 cm and height of 100 or 160 cm (Table 3), packed with green coconut husks (480–600 g) (Table 3), for decontamination of effluent containing Cu(II), Ni(II), and/or Zn(II). For monocomponent systems, removal capacities of 0.746, 0.452, and 0.439 mmol g^−1^ (47.41, 26.53, and 25.76 mg g^−1^) were reported for Cu(II), Ni(II), and Zn(II), respectively, while for the three-component system, the removal capacities were 0.509, 0.185, and 0.121 mmol g^−1^ (32.35, 10.86, and 7.91 mg g^−1^) (Table 2), respectively [48].

Although the experimental conditions in the mono- and multicomponent systems were not equimolar, a notable competitive adsorption effect was observed in the multicomponent experiments, suggesting a greater affinity of the bioadsorbent for Cu(II) ions. The cumulative adsorption capacity in the ternary-component system (0.815 mmol g^−1^) was slightly higher than the adsorption capacity for Cu(II) (0.746 mmol g^−1^, the best result obtained in the monocomponent systems) [48]. This suggested that all the active sites of the bioadsorbent were filled by metal ion species, with no participation of additional adsorption sites, which could explain the strong reduction in the adsorption capacity for metals in the multicomponent system, compared to the individual adsorption capacities in the monocomponent systems [48].

Gagnon et al. [89] chemically modified a softwood (*Pinus* spp.) with phosphoric acid to introduce phosphate groups (PLF) to remove Fe, Mn, and Zn ions. To mitigate the high concentrations of these ions in acid drainage water originating from an abandoned mine, a mobile treatment system with a series of adsorption columns was developed. The system included an initial column containing 290 kg of limestone gravel, followed by four adsorption columns with diameter of 54 cm and bed height of 60 cm, each containing 3.55 kg of PLF (dry weight) mixed with silica sand (using a cement mixer). The system successfully treated nearly 5000 L of acid drainage water, achieving a removal capacity of 1635 mg g^−1^ (29.27 mmol g^−1^) for Fe, 23.1 mg g^−1^ (0.42 mmol g^−1^) for Mn, and 18.1 mg g^−1^ (0.28 mmol g^−1^) for Zn (Table 2) [89]. This treatment system demonstrated that a chemically modified lignocellulosic material can be successfully applied on an industrial scale for the treatment of a complex effluent containing inorganic pollutants, as well as to mitigate environmental impacts caused by mining activities.

Advances in computational tools, including artificial neural networks and machine learning [43,79,106,116], have enabled simulations to predict column performance. Nonetheless, maintaining proportionality between larger and smaller scale operations usually leads to small variations in maximum adsorption capacity, efficiency of the adsorption process, and effective use of the bed [132]. Under satisfactorily controlled operating conditions, mathematical models can be considered fundamental tools for predicting, describing, and understanding the behavior and performance of adsorption columns.

## 6. Simplified Models for Monocomponent Fixed-Bed Columns

### 6.1. Redundant Comparisons and Misuse of Models

Monocomponent column adsorption models allow temporal evaluation of the concentration of a solute species in the column. The most common simplified models for dynamic adsorption in a column are the Bohart–Adams [11], Thomas [12], Yoon–Nelson [13], Wolborska [14], and Bed Depth Service Time (BDST) models (Supplementary Materials). These models consider local equilibrium approaches without resistance to intraparticle diffusion and axial dispersion. Around 60% of the articles considered in this review used at least one of these models. It is quite common to find studies comparing these models, although it should be noted that it is only in their simplified forms that they are mathematically identical [15].

Chu [17] used some mathematical deductions to reveal that the Bohart–Adams and Thomas models are mathematically equivalent, with the main difference between them being the type of adsorption isotherm describing the adsorption equilibrium. The Bohart–Adams model is based on the premise that adsorption equilibrium can be described by a rectangular irreversible isotherm, while the Thomas model is based on the assumption that adsorption equilibrium can be described by a favorable Langmuir isotherm [61]. Chu [17] demonstrated that the simplified Bohart–Adams, Thomas, and Yoon–Nelson models can be generically described by a logistic population growth equation (Equation (3)). The logistic equation profile shows that as column saturation is reached, the output concentration reaches higher asymptotic values, with the curve presenting symmetrical sigmoidal behavior [15]. Table 4 provides the parameters of the simplified models, expressed in terms of the fitting parameters “*X*” and “*Y*” of a generalized logistic equation.

(3) $$\frac{C}{C_{0}}=\frac{1}{1+e^{\left(X-Yt\right)}}$$

Chu [15] reported that in the environmental research area, there is an erroneously simplified Bohart–Adams model (Equation (4a)), where a unit value (−1) of the correct Bohart–Adams equation (Equation (4b)) has been unduly ignored. This leads to a serious problem, because the exponential behavior of the incorrect equation only allows detection of the experimental data points at the beginning of the breakthrough curve, making it unsuitable for dynamic adsorption modeling [15].

(4a) $$\ln\left(\frac{C_{0}}{C}\right)=\frac{k_{BA}N_{0}L}{u}-k_{BA}C_{0}t$$

(4b) $$\ln\left(\frac{C_{0}}{C}-1\right)=\frac{k_{BA}N_{0}L}{u}-k_{BA}C_{0}t$$

The Wolborska model also has an erroneous equation version (Equation (5)) in the literature [15]. Although it has been corrected by the authors themselves [133], this equation presents the same mistake mentioned above for the Bohart–Adams equation.

(5) $$\ln\left(\frac{C_{0}}{C}\right)=\frac{\epsilon \beta L}{u}-\frac{\varepsilon \beta C_{0}L}{N_{0}}\;$$

The main general problem related to the use of these incorrect equations is that they are less predictive than the others. Consequently, they have been discarded in many studies, because they are not able to describe the behaviors of typical breakthrough curves [15]. Among the articles evaluated, 27 (33%) used the Bohart–Adams equation, with 13 (48%) using incorrect forms of the Bohart–Adams or Wolborska equations.

Recent works, due to inattention, continue to use this flawed equation. Chen et al. [38] investigated the removal of Cr(VI) by continuous adsorption in fixed-bed columns packed with chemically modified corn stalk, where the poor predictive capacity of the incorrect Bohart–Adams model was confirmed by its lower *R*^2^ values (0.59–0.88), compared to the Thomas (0.74–0.95) and Yoon–Nelson (0.84–0.97) models.

Safardastgerdi et al. [91] chemically modified a medicinal plant (*Gundelia tournefortii*, commonly found on the Asian continent) with ethylenediaminetetraacetic dianhydride (EDTAD), to produce a bioadsorbent for Cu(II) removal. In continuous adsorption (see the column operating parameters in Table 2), an adsorption capacity of 15.61 mg g^−1^ (0.25 mmol g^−1^) was achieved, with an effective use of the bed (*Z*_e_) of 53.8% (Table 2).

**Figure 7:**
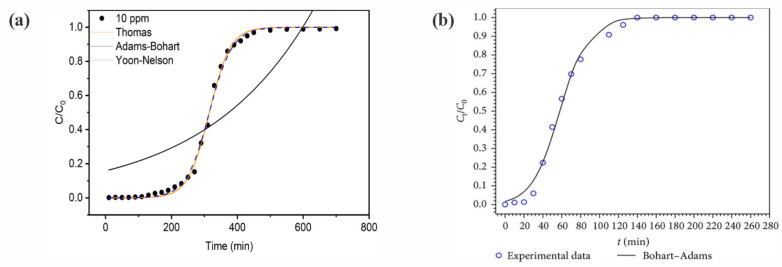
(**a**) Breakthrough curve for Cu (II) adsorption on *Gundelia tournefortii* chemically modified with EDTAD modeled using the incorrectly propagated Bohart–Adams model. Reprinted from Minerals Engineering, Vol. 204, Safardastgerdi et al. [91], Copyright (2023), with permission from Elsevier. (**b**) Breakthrough curve for Cu(II) adsorption on sugarcane bagasse chemically modified with H_3_PO_4_/NaNO_2_ modeled using the correct Bohart–Adams model. Reprinted from Advances in Polymer Technology, Vol. 2020, Rodrigues et al. [67], Copyright (2020).

Figure 7a presents the curves resulting from the fitting of the Thomas, Bohart–Adams, and Yoon–Nelson models to Cu(II) adsorption on *Gundelia tournefortii* modified with EDTAD to describe the monocomponent column adsorption [91]. Figure 7a illustrates the use of the incorrectly propagated Bohart–Adams model, which did not provide a good fit (*R*^2^ = 0.667 to 0.788) to the experimental data [91]. It also demonstrates the similarity between the Thomas and Yoon–Nelson models, which are mathematically identical in their simplified forms.

When the correct monocomponent Bohart–Adams model equation is used, the fitting is identical to the fits provided by the Thomas and Yoon–Nelson models. Figure 7b presents a curve resulting from the fitting of the correct Bohart–Adams model to Cu(II) adsorption in a fixed-bed column consisting of a chemically modified (with H_3_PO_4_ and NaNO_2_) bioadsorbent derived from sugarcane bagasse [67]. The model presented *R*^2^ > 0.97, accurately describing the experimental data in the breakthrough curve.

According to Chu [15], modeling continuous adsorption data with the Bohart–Adams model, as described here for the work by Safardastgerdi et al. [91], may have contributed to the Bohart–Adams model falling into disuse, with other models being considered more predictive.

The misapplication of simplified models and the lack of strength comparison criteria reinforce the need for the scientific community to recognize that parameter interpretations that claim to be based on sound physicochemical principles may lack validity when derived from oversimplified models. Understanding these biases is crucial for ensuring the accuracy of research results.

### 6.2. Data Distortion by Linearized Models

Mathematical models are essential for the interpretation of experimental data and prediction of column operation in industrial continuous adsorption processes [134]. To facilitate practical applicability, mathematical simplifications of the models can be employed to describe the adsorption behavior, without great loss of precision. Complex nonlinear regression methods, such as the least squares method, have become more accessible, and computer technology has simplified their application. Since kinetic and isotherm models are now well described by nonlinear equations available in the literature, the use of linearized models and linear regression is no longer recommended [135]. Linearized model equations can introduce structural errors in the data that can alter the statistical interpretation of experimental error distributions, invalidating the least squares assumptions of constant error variance and normal fluctuation of measurement errors [136,137]. The linearized model forms used extensively in the adsorption literature sometimes do not consider the basic assumption of data homoscedasticity necessary for application of the least squares method. In addition, the statistical tests used to evaluate the quality of fit to the experimental data often fail to detect parameter bias [137].

Despite the arguments against the use of linearized statistical models and the ready availability of nonlinear regression algorithms, adsorption studies commonly use linearized models to obtain fitting parameters for kinetic, isotherm, and breakthrough curves [136,138]. Although they are based on the same assumptions, the results obtained from fitting nonlinear and linear models to the experimental data differ significantly, mainly due to the mathematical manipulation used in linearization methods, which usually leads to poorer results for linear models, compared to nonlinear models [137]. Regarding the equations developed for continuous adsorption, González-López et al. [134] performed an in-depth analysis using simulated data to determine the accuracies of linear and nonlinear forms of the Thomas model. As expected, the nonlinear equation proved to be more robust and less susceptible to experimental errors, with 0.5% relative error between the experimental and predicted data [134]. For cases when it is not possible to use nonlinear regression, McCuen and Surbeck [138] provided some general guidelines to avoid inaccurate results due to errors generated by linearization methods.

### 6.3. Statistical Criteria for Evaluating Adsorption Models

In adsorption data modeling, it is very common to compare the predictive ability of mathematical models using the coefficient of determination (*R*^2^) (Supplementary Materials) obtained from fitting a model to experimental data [138]. However, this type of comparison is unable to provide accurate conclusions for several reasons: (1) *R*^2^ is sensitive to extreme experimental data points, resulting in misleading results of quality of fit tests; (2) *R*^2^ is directly influenced by the range of results for the dependent variable; and (3) *R*^2^ tends to be higher when more parameters are added to the model, decreasing the degrees of freedom for error estimation [136,138].

To overcome these problems, it is recommended to ignore extreme data points (outliers) and avoid linear transformation of the model equation, as well as to use a more suitable objective function, depending on the complexity of the model and the degree of dependence of the variables, such as the adjusted coefficient of determination (*R*^2^_adj_), chi-square (*χ*^2^), reduced chi-square (*χ*^2^_red_), and the Akaike Information Criterion (AIC), among others [136]. It is also essential to ensure a proper understanding of the physical meaning of the model input and output parameters, including kinetic and equilibrium parameters, to avoid drawing inaccurate conclusions for subsequent modeling of the breakthrough curves. As more input parameters are entered, the column adsorption models become more difficult to implement. However, statistical tools such as design of experiments (Supplementary Materials) can be an efficient procedure for planning and analyzing adsorption experiments, allowing objective conclusions to be drawn.

## 7. Multicomponent Adsorption in Fixed-Bed Columns

### 7.1. Competitive Adsorption and Overshooting Phenomenon

Column adsorption studies using spiked monocomponent aqueous solutions are most common in the literature and have been well designed and discussed with respect to experimental data modeling. However, for more complex contaminated water systems, including industrial effluents, the well-known adsorption models become less predictive [139]. Competition between the chemical species in solution for the active adsorption sites, as well as repulsive or attractive lateral interactions between adsorbed species on the surface of the bioadsorbent, are the main factors responsible for this prediction inaccuracy [140,141]. The ratio (*r*) between the adsorption capacities for monocomponent (*q*_mono_) and multicomponent (*q*_multi_) systems reflects the degree of interference of species *i* in the adsorption of species *j*, with *r* < 1 for synergistic interaction, *r* > 1 for antagonistic interaction, and *r* ≅ 1 for no significant interaction. In practice, synergism implies increased adsorption capacities in multicomponent systems, while antagonism implies reduced adsorption capacities, either for the individual component or for all components in the system [142].

**Figure 8:**
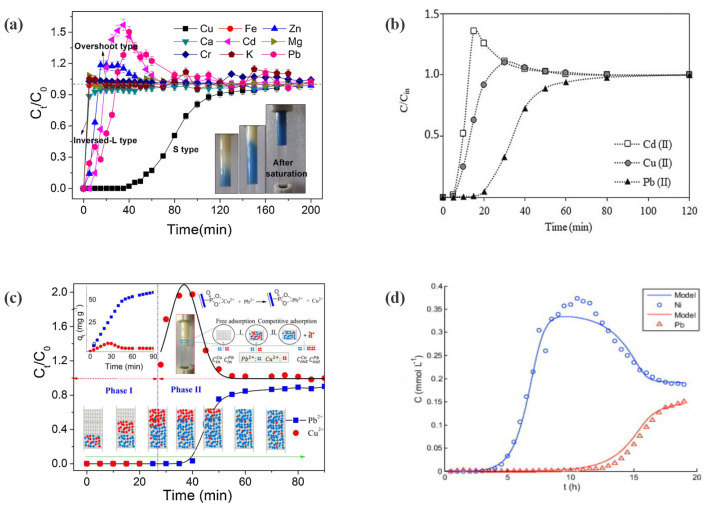
Illustration of the overshooting phenomenon in adsorption studies using fixed-bed columns packed with adsorbents for the treatment of complex multicomponent solutions containing inorganic pollutants: (**a**) Breakthrough curves for adsorption of metal ions on TEPA-modified SCB. Reprinted from Process Safety and Environmental Protection, Vol. 116, Xie et al. [143], Copyright (2018), with permission from Elsevier. (**b**) Breakthrough curves for adsorption of Cd(II), Cu(II), and Pb(II) on pine cone shell (*Pinus sylvestris*). Reprinted from Science of the Total Environment, Vol. 917, Amar et al. [88], Copyright (2024), with permission from Elsevier. (**c**) Breakthrough curves for binary adsorption of Pb^2+^ and Cu^2+^ on PA-modified SCB. Reprinted from Process Safety and Environmental Protection, Vol. 124, Xiong et al. [71], Copyright (2019), with permission from Elsevier. (**d**) Breakthrough curves for binary adsorption of Ni(II) and Pb(II) on grape stalks (*Vitis vinifera*). Reprinted from Chemical Engineering Journal, Vol. 217, Escudero et al. [39], Copyright (2013), with permission from Elsevier.

Overshooting is a very common phenomenon in dynamic multicomponent bioadsorption, occurring when the outlet concentration of one or more components exceeds the inlet concentration. According to Escudero et al. [39], at the beginning, there is a large quantity of binding sites available on the surface of the adsorbent, so even low-affinity adsorbate species can bind to the adsorption sites, without competition among the species in solution. As the system approaches column saturation and the availability of binding sites decreases, selectivity and competition lead to the adsorbate species with higher affinity displacing the lower affinity species already adsorbed, releasing them from the binding sites to the solution. Consequently, the outlet concentration of the displaced species becomes higher, and the overshooting phenomenon can be observed [35,36,60,61]. Figure 8 illustrates the overshooting phenomenon occurring in continuous adsorption for a multicomponent solution or effluent containing inorganic pollutants, using columns packed with different lignocellulosic adsorbents (see Table 2 for the operating parameters).

**Figure 9:**
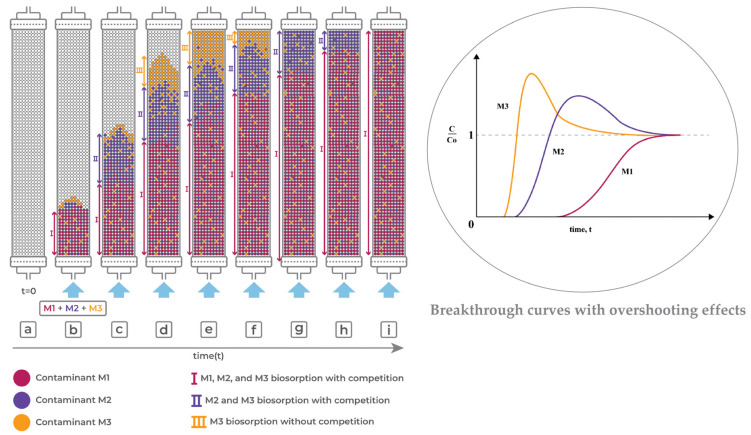
Scheme of the adsorption process for a tricomponent contaminant solution in a fixed-bed column. Adapted from Kleinübing et al. [144].

For a better understanding of the adsorption behavior in a multicomponent system where adsorbate species have different affinities for the surface active sites of an adsorbent, a scheme (Figure 9) representing the adsorption process for a tricomponent contaminant solution in a fixed-bed column was adapted from Kleinübing et al. [144].

According to the strength of affinity of the active sites of the adsorbent for each adsorbate species, it is possible to predict the order in which the species will leave the fixed-bed column at the outlet. This ternary system contains three inorganic contaminants, namely, M1, M2, and M3, with the following order of affinity for the active surface sites: M1 > M2 > M3. The adsorption column will behave analogously to a chromatographic column retaining contaminants for different times. As the column (a) is fed with an influent containing three contaminants (b), the contaminants with lowest affinities for the active sites of the surface are displaced, so they are retained in the column for a shorter time. The contaminant with the highest affinity (M1) will preferentially be retained in the lower region of the column, called region (I). The upward flow of the solution (c) becomes more concentrated with contaminants of lower affinities (M2, M3), forming a second region, called region (II), with competition mainly between species M2 and M3. In region (II), the contaminant of lowest affinity (M3) is displaced, forming a monocomponent region, called region (III), without the occurrence of competitive adsorption (d). The adsorption process will continue until complete saturation of the column.

The contaminant with the highest affinity will not necessarily occupy 100% of the active surface sites, while the contaminant with the lowest affinity may reach a minimum equilibrium concentration. Figure 9 also presents an example of a breakthrough curve for a tricomponent mixture. The contaminant of highest affinity (M1) tends to displace contaminants of lower affinity (M2 and M3), causing the overshooting of these species. Other effects, such as the diffusivity of each contaminant species and column operating conditions, can also influence column adsorption behavior, with overshooting effects not always being perceived under multicomponent conditions [130].

### 7.2. Evaluation of Adsorption Affinity Behavior in Fixed-Bed Columns

The surface heterogeneity of bioadsorbents allows a combination of different adsorption mechanisms, such as ion exchange, electrostatic interaction, and complexation [31]. The greater the number of mechanisms involved, the more complex it becomes to determine the adsorbate–adsorbent affinity order and to understand the antagonistic or synergistic effects between adsorbed species on the surface of the adsorbent [31]. Table 5 presents the adsorption affinity order according to the adsorption mechanisms and the properties of the contaminant ions. Table S5 provides a detailed description of the properties of the species used to predict the affinity order for the active sites of the bioadsorbents.

The selectivity of bioadsorbents for different metal ions depends on several physicochemical properties, such as valence, ionic charge to ionic radius ratio, electronegativity, and chemical speciation as a function of solution pH, among others [31,145]. There have been several studies providing information on the degree of affinity between the chemical groups (binding sites) of the adsorbent and metal ions. Irving and Williams [146] found that the stability of transition metal complexes formed by divalent ions decreased with metal cation size, in the following order: Mn(II) < Fe(II) < Co(II) < Ni(II) < Cu(II) < Zn(II). The affinity order of metals (Table 5), described by the Irving–Williams series, is independent of the ligand group [146]. Varadwaj et al. [147] investigated the stability constant (*β*_L_) of various ligands of the Irving–Williams metal series and reported the same suggested order. The stability of the metal–ligand complexes formed can be explained by the ionic radius and the second ionization potential of the target metal ions [148].

As reported by Varadwaj et al. [147], Pearson [149] proposed a complementation of the Lewis acid–base theory, called “Hard and Soft Acids and Bases” (HSAB), or Pearson acid–base concept, which could explain the stability order in the Irving–Williams series. Pearson [149] stated that hard cations form more stable complexes with hard ligands, while soft cations form more stable complexes with soft ligands. For ligands and cations of different classifications, including complexes formed by hard ligands with soft cations, and by soft ligands with hard cations, the metal–ligand complexes formed are generally less stable [149]. The absolute hardness (*ƞ*) of Lewis acids and bases is a way to quantify and compare species in terms of degree of hardness. The *ƞ* value can be evaluated using the difference between the ionization energy and the electron affinity of the atom [150].

The Irving–Williams series and HSAB theory have been used to explain the order of affinity of metal cations for the binding sites of raw and modified lignocellulosic materials [3], but when different metals of the series are analyzed, different properties are used to explain the observed affinity order (Table 5). Table S5 (Supplementary Materials) summarizes the properties of the metals most used in adsorption studies employing raw and modified lignocellulosic adsorbents. These properties were used to predict or explain the order of affinity of an adsorbate for a given adsorbent, as well as the adsorption mechanism involved.

The order of affinity of metal ions for adsorption sites can also be correlated with the adsorption mechanisms. When the main adsorption mechanisms in multicomponent systems involving metal cations are electrostatic interaction, physical adsorption, and ion exchange, there is a tendency in the literature to evaluate the affinity order of metal cations by considering the ionic character of the metal, including electronegativity and hydrated ionic radius [151]. In turn, when covalent bond, complexation, and oxidation-reduction reactions are dominant, greater affinities tend to be observed for metals with higher reduction potential and electronegativity (Pauling) [145].

**Table 5:** Affinity order of the main bivalent species studied, according to the principal mechanisms involved in adsorption, with suggested explanations based on the property evaluated.

Main Mechanism	Property	Explanation	Order of Affinity	Reference
Electrostatic interaction (electrostatic attraction and ion exchange)	Ionic radius	Ions with greater charge and smaller hydrated radius present greater polarization, facilitating electrostatic interaction	Ni(II) > Cu(II) = Co(II) > Zn(II) > Cd(II) > Hg(II) > Pb(II)	[145,152]
	Hydrated ionic radius		Pb(II) > Ni(II) > Cu(II) > Hg(II) > Co(II) > Cd(II) > Zn(II)	[145,152]
	Ionic potential (ionic charge/ionic radius)	Stronger bonds are formed with ions of higher charge/radius ratios	Pb(II) > Ni(II) > Cu(II) > Hg(II) > Co(II) > Cd(II) > Zn(II)	[145,153]
	Solubility product constant of phosphate salts (*K*_sp_)	Metal ions with higher *K*_sp_ and ligand groups (e.g., PO_4_^3−^) show higher affinity	Ni(II) > Zn(II) > Cd(II) > Co(II) > Pb(II)	[145,154]
Covalent bond, complexation, and redox reaction	Irving–Williams series (ionic radius and second ionization potential energy)	Metal ions with smaller ionic radii form more stable complexes, but for transition metals, bond strength will depend on the radius and electron configuration	Mn(II) < Fe(II) < Co(II) < Ni(II) < Cu(II) > Zn(II)	[145,146]
	Electronegativity (Pauling)	Metals with higher electronegativity form stronger covalent bonds with oxygen-containing ligands	Cu(II) = Hg(II) > Ni(II) > Co(II) > Pb(II) > Fe(II) > Cd(II) > Zn(II) > Mn(II)	[145,155]
	Standard reduction potential	Metals with higher reduction potential tend to form stronger bonds with electron-donating ligands	Hg(II) > Pb(II) > Ni(II) > Co(II) > Cu(II) > Cd(II) > Zn(II)	[145,154]
	Complex formation constant	Metals with higher complex formation constants form more stable complexes, similar to the Irving–Williams series but with the introduction of Pb(II)	Hg(II) > Cu(II) > Ni(II) > Co(II) > Zn(II) > Cd(II)	[145,154]

An effective approach for improving the selectivity towards a species in multicomponent systems is the incorporation of soluble complexing ligands, such as ethylenediaminetetraacetic acid (EDTA), which can mask the affinity of a species by complex formation [156]. Comparative assessment of the complex formation constants (p*K*_f_) for ligands/metals enables a more selective separation, since a greater difference between the p*K*_f_ values of the metal ions competing for the ligand makes it easier to separate the metals [156].

Xiong et al. [71] introduced a phosphate group in the sugarcane bagasse cellulose structure by chemical modification with phosphoric acid, followed by use of the modified material in fixed-bed column adsorption tests for the removal of Pb(II), Cu(II), Cd(II), Zn(II), and Ca(II) from a multicomponent solution. In addition to identifying a main contribution of ion exchange in the adsorption process, the results showed that the affinity order could be defined according to the ionic charge to hydrated ionic radius ratio and electronegativity, resulting in the following affinity order: Pb(II) > Cu(II) > Cd(II) > Zn(II) > Ca(II) [71]. This order may also be related to the solubility product constant (*K*_sp_) of the phosphate salts of the metals, since the metals forming the most soluble phosphate salts had the highest affinity [73].

A similar result was reported by Yu et al. [157], who used pyromellitic dianhydride as a modifying agent to introduce carboxylic functionality in the structure of sugarcane bagasse. The main mechanism involved was ion exchange and the reported affinity order was Pb(II) > Cu(II) > Cd(II) > Zn(II) [157]. Except for Pb(II), the stabilities of the complexes followed the Irving–Williams series [157].

In column adsorption tests using grape stalk waste with binary mixtures composed of Cu(II), Cd(II), Ni(II), and Pb(II), Escudero et al. [39] observed the overshooting phenomenon, with the affinity for the adsorption sites following the order Pb(II) > Cu(II) > Cd(II) > Ni(II). The stabilities of the complexes also followed the Irving–Williams series [39].

Table 5 can provide a basis for reverse engineering of lignocellulosic bioadsorbents by researchers, whether raw or chemically modified, to save resources and experimental effort in the laboratory, by predicting the adsorption behaviors based on the binding groups and the contaminant species.

## 8. Modeling Fixed-Bed Columns in Multicomponent Systems

### 8.1. Mass Transfer Models for Fixed-Bed Columns

For multicomponent adsorption using batch or fixed-bed columns packed with bioadsorbents based on lignocellulosic biomass, there are few reported studies that have used mathematical modeling to investigate the effects of competition and interaction between the adsorbate species in solution and on the surface of the adsorbent [39,71,76]. The main challenge lies in the increasing complexity of factors influencing adsorption behavior in column systems. This requires mathematical expertise, which often prevents many studies from engaging in more in-depth discussions and increasing their maturity levels. Therefore, this is problematic, as practical applications are not limited to monocomponent systems, or even to “ideal” behavior (with an absence of interaction effects among solutes) of each solute in multicomponent systems.

Mass transfer models for column adsorption account for transport mechanisms and adsorption equilibrium, as illustrated in Figure 1. The general mass transfer equation for fixed-bed columns, detailed in the Supplementary Materials (Equation (S19)), is expressed as follows, considering a one-dimensional mass balance and axial dispersion [130]:

(6) $${v_{F}\;\frac{\partial c_{i}\left(t,Z\right)}{\partial Z}+\varepsilon_{b}\frac{\partial c_{i}\left(t,Z\right)}{\partial t}+\rho_{b}\;\frac{\partial \bar{q}\left(t,Z\right)}{\;\partial t}-D}_{ax}\varepsilon_{b}\frac{\partial^{2}c_{i}\left(t,Z\right)}{\partial Z^{2}}=0$$

where

- $v_{F}\frac{\partial c_{i}\left(t,Z\right)}{\partial Z}$ represents solute convective transport along the column in the *Z* direction over time;
- $\varepsilon_{b}\frac{\partial c_{i}\left(t,Z\right)}{\partial t}$ describes the rate of change (accumulation) in solute concentration in the liquid phase;
- $\rho_{b}\frac{\partial \bar{q}\left(t,Z\right)}{\partial t}$ represents the rate of adsorption of the solute on the solid phase;
- $D_{ax}\varepsilon_{b}\frac{\partial^{2}c_{i}\left(t,Z\right)}{\partial Z^{2}}$ accounts for solute dispersion along the flow direction, due to velocity variations and diffusion effects.

Since this is a partial differential equation, solving it requires defining the initial and boundary conditions, discretization, and iterative numerical methods. To simplify the mathematical application, the equation terms are converted into their dimensionless forms. The Supplementary Material details two mass transfer models for column adsorption: the Homogeneous Surface Diffusion Model (HSDM) and the Linear Driving Force (LDF) model.

#### 8.1.1. HSDM and LDF Models

The HSDM assumes that mass transfer occurs along the external (film) and internal surfaces of the adsorbent particles (with diffusion in the liquid phase being negligible), and that the equilibrium at the interface between the liquid and solid phases is established instantaneously. The diffusion on the adsorbent particles is treated as homogeneous, without substantial variation of the surface diffusion coefficient, although it can depend on the adsorbate concentration. Therefore, this model integrates the mass balance equation, considering film diffusion and surface diffusion, while neglecting axial dispersion [39,130]. It is important to note that if axial dispersion cannot be neglected (for example, at low fluid velocities or in small-scale systems), the use of more advanced models should be considered as a more reliable and accurate option. The LDF model provides a simplified approach using ordinary differential equations (ODEs) to describe the mass transfer between the liquid and solid phases, facilitating numerical calculations. In this model, the adsorption rate is directly proportional to the linear driving force, corresponding to the difference between the amount of adsorbate adsorbed on the particle surface at equilibrium and at a given time. This model also simplifies the diffusion on the particle surface, without the need to solve the Fick equation for diffusion, so it should therefore be used to describe adsorption systems where intraparticle diffusion is the rate-limiting step [130].

Escudero et al. [39] modeled adsorption in a fixed-bed column packed with grape stalks, considering binary systems including the ions Cu(II), Pb(II), Ni(II), and Cd(II) (Table 2). The modified Langmuir isotherm model for binary systems adopted the HSDM as the mass transfer model, assuming homogeneous adsorption, with mass transfer according to the coupled outer film and surface diffusion mechanisms [158]. The partial equations discretization method used was finite elements [39]. The mass balance of each component and surface diffusion on the solid phase were calculated by progressive differences and the Crank–Nicolson method, respectively [39]. The models provided reasonable fits, describing the overshooting phenomenon caused by competitive adsorption [39]. In the presence of Cu(II) or Pb(II), there was more than 60% desorption of Ni(II) and Cd(II) ions. The diffusion coefficients obtained by the model for the contaminant species in binary mixtures were higher than the diffusion coefficients obtained for monocomponent solutions. On the other hand, the mass transfer coefficients of the contaminant species remained the same for mono- and bicomponent systems [39].

Davila-Guzman et al. [113] modeled their adsorption studies using adsorbents derived from spent coffee grounds, employing the LDF model to describe the column breakthrough curves for the removal of the metals Cu(II), Pb(II), and Cd(II) (Table 2). The mass transfer model was able to accurately predict the breakthrough curves of the ions from the start to the saturation time [113]. The results showed that the external mass transfer coefficient for Pb^2+^ adsorption was the highest, suggesting faster diffusion, and that axial dispersion was a significant phenomenon in the column adsorption. The affinity order in the ternary system was Pb(II) > Cu(II) > Cd(II) [113].

The isotherm equations are coupled with mass transfer models. The use of multicomponent models that account for competitive adsorption is essential to accurately describe potential overshooting effects.

#### 8.1.2. Multicomponent Isotherm Equations

Batch adsorption isotherms are mathematical model equations used to quantitatively describe the adsorption of solutes by solids at a fixed temperature. A batch adsorption isotherm shows the amount of a given solute adsorbed by an adsorbent as a function of the equilibrium concentration of the solute [16,140]. However, in continuous adsorption, a local equilibrium is assumed to be established along the column, between the mobile (fluid) phase and the stationary (solid) phase [9].

Multicomponent adsorption isotherm models enable the description of more complex adsorption systems, typical of industrial effluents, where interference between different adsorbate species may occur due to competitive adsorption [159]. By coupling the multicomponent adsorption isotherm model equations to mass transfer models, it is possible to describe the overshooting phenomenon in multicomponent experiments [16]. The main isotherm models available in the literature for multicomponent systems are presented in Table S3 (Supplementary Material).

Some multicomponent models are obtained by extending the monocomponent models to pure components, without introducing fitting parameters in the formulas. This allows evaluation of deviations from ideality caused by competitive adsorption, as a function of synergistic or antagonistic effects between adsorbed species [141].

Extended models, such as the extended Langmuir model proposed by Butler and Ockrent [160], consider that the saturation capacity and thermodynamic properties of the system are equal for all adsorbate species present in both mono- and multicomponent systems, which is thermodynamically inconsistent when the maximum adsorption capacities and affinities of all components for the adsorption sites do not have the same value [130,161].

The Ideal Adsorbed Solution Theory (IAST) model [23] has thermodynamic foundations and also takes into account different adsorption capacities and affinities of the adsorbed species [130,161]. The IAST model is based on ideal behavior of the *i* components of the mixture, without synergistic or antagonistic interaction between the components, according to Henry’s law [162]. For the liquid phase, in dilute solutions, ideal behavior of the adsorbate species can be considered. However, for the adsorbed phase, the interactions between adsorbates cannot be disregarded for solids with high saturation capacities.

Despite its reasonable success when applied to multicomponent systems closer to ideality, use of the IAST model and its modified forms is hindered by the complexity of the algorithms, requiring implementation using computer software and knowledge of programming language [163].

Modified isotherm models include new variables and/or interactive parameters to take account of synergistic or antagonistic effects in multicomponent adsorption systems. Schay [164] proposed the addition of a fitting parameter in the extended Langmuir model by including a characteristic interaction term, *η_i_*, for each adsorbate species, depending on the concentration in the liquid phase [161,165]. Fritz and Schluender [166] and Sheindorf et al. [167] developed modified Freundlich models, with the former restricted to bicomponent adsorption and the latter specific to the antagonistic effects of competitive adsorption [141].

McKay and Al Duri [168] proposed an isotherm model to evaluate the deviation from ideality in a multicomponent system, implicitly assuming the existence of competitive adsorption, with addition of the *P_i_* factor to the Langmuir isotherm model. The so-called *P_i_*-factor model does not take into account the interaction between adsorbed species, but it adds an additional fitting parameter that allows the effects of competitive adsorption to be evaluated using a lumped factor (*P_i_*) [169].

Costa et al. [170] developed the Real Adsorbed Solution Theory (RAST) model, an extension of the IAST model, for adsorption of non-ideal mixtures, where the hypothesis of deviation from ideality is assumed, introducing a new fitting parameter called the activity coefficient (*γ_i_*) (Supplementary Materials), which considers the possible occurrence of interactions between the components of the system [162].

The complexity of multicomponent adsorption on lignocellulosic adsorbents makes prediction of the behavior of the system difficult, even for models that take competitive adsorption into account [47,141]. It is common for multicomponent models to provide poor predictions for mixtures with very low concentrations of adsorbate species, since they behave ideally at such concentrations, which is characteristic of adsorption isotherm models where the behavior is linear at very low concentrations [171]. In addition, the quality of the fit is worse for systems with more than two components [141].

Despite these limitations, reasonable fits have been reported for adsorption systems using raw and modified lignocellulosic materials [39,42,162,169]. Some studies have evaluated simpler alternative models for use in describing breakthrough curves for multicomponent systems.

### 8.2. Bell-Shaped Curve Modeling

To overcome the lack of prediction of multicomponent breakthrough curves by the models, Chatterjee and Schiewer [76] and Xiong et al. [71] noted that the overshooting phenomenon is mainly caused by the desorption of the “weaker” (lower affinity) metal ion by the “stronger” (higher affinity) metal ion. The overshooting peaks generated can be described as a bell-shaped curve, typical of a normal or Gaussian distribution (Equation (7)), which is commonly used in liquid chromatography techniques [71].

(7) $$\frac{C}{C_{0}}=Z_{0}^{\prime }+\frac{A^{\prime }}{w^{\prime }\sqrt{\pi /2}}e^{-2{\left(\frac{t-t_{c}^{\prime }}{w^{\prime }}\right)}^{2}}$$

where $Z_{0}^{\prime }$ (1.0) is the offset (equal to $\frac{C_{e}}{C_{0}}$), $A^{\prime }$ is the area of the “desorption curve peak” (min), $t_{c}^{\prime }$ is the center of the “desorption curve peak” (min) (equal to maximum of $\frac{C}{C_{0}}$), $w^{\prime }$ is the half width of the “desorption curve peak” (min), and *t* is the given time (min).

**Figure 10:**
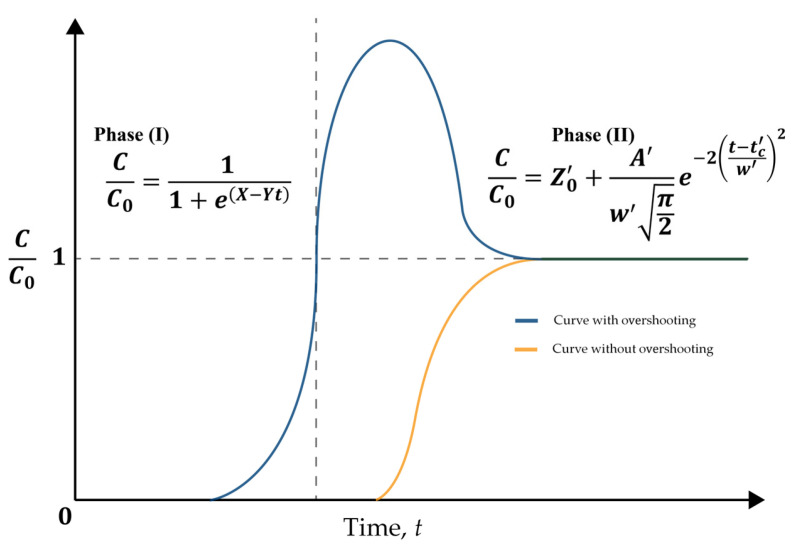
Column breakthrough curves for multicomponent adsorption: the yellow breakthrough curve represents a “stronger” species adsorbed without overshooting and the blue breakthrough curve represents a “weaker” species adsorbed with overshooting (phase I is modeled using a sigmoidal equation (Equation (3)) and phase II is modeled using a bell-type equation (Equation (7)) for overshooting.

Figure 10 illustrates the concept of this alternative modeling approach. In phase (I), the breakthrough curve for the species with the lowest affinity is described using sigmoidal equations, characteristic of monocomponent models, until it reaches its saturation point. Beyond this point, phase (II) begins, where the breakthrough curve is best represented by the equation for a bell-shaped curve.

Chatterjee and Schiewer [76] used a generic sigmoidal equation suggested by Chu [17] (Equation (3)) to describe the breakthrough curve for the weaker adsorbed metal ion, up to the 95% saturation point. From this point, a bell-type equation for desorption (Equation (7)) began to describe the adsorption dynamics in the column packed with a bioadsorbent based on citrus peel. The fitting parameters *A* and *B* (min^−2^), shown in Equation (8), were obtained empirically by Chatterjee and Schiewer [76], and the graphical results indicated reasonable fits.

(8) $$\frac{C}{C_{0}}=A\;t^{2}e^{-Bt^{2}}$$

### 8.3. Quantitative Structure–Activity Relationship (QSAR) Approach

Despite being rarely used in the area of lignocellulosic adsorbents for the treatment of water contaminated with inorganic species, models based on the Quantitative Structure–Activity Relationship (QSAR) concept can be used to obtain quantitative relationships between the chemical structures of adsorbents and their physicochemical activities [172]. QSAR models are mathematical tools that establish correlations between the structural variables and adsorption activities of adsorbents, providing a detailed analysis of the process at the molecular level [173]. QSAR analysis enables evaluation of specific molecular properties that play significant roles in adsorption mechanisms [174]. This approach enables prediction of the selectivity and adsorption capacity of adsorbents, contributing to a better understanding of the performance of bioadsorbents in removing inorganic contaminants from water.

### 8.4. Modeling Using Artificial Neural Networks (ANNs)

As discussed previously, the modeling of adsorption columns is complex, because it involves several parameters connected with the system conditions. This complexity increases as the number of contaminant species in the multicomponent system increases. The interaction between the adsorbate species can lead to synergistic or antagonistic effects, as well as the overshooting phenomenon. The multicomponent adsorption models based on mass balances are difficult to implement and have limitations related to the assumptions adopted [175]. As an alternative, artificial neural network methods have emerged as powerful tools for more straightforward modeling and prediction of adsorption column data. ANNs use machine learning training to identify, recognize, and classify data, enabling the prediction of future events and estimation of nonlinear relationships of the input and output parameters selected for modeling [176]. Stochastic optimization methods including particle swarm optimization (PSO) [177] or genetic algorithms (GAs) [178] are used to assess the weights and biases of the network model. Among the articles compiled in this review containing column adsorption data for bioadsorbents, only three studies applied ANNs for analysis of the adsorption data [43,106,178,179] (Table 2), while only one study employed this computational tool for column modeling [43].

Calero et al. [43] studied the application of bioadsorbents based on olive stone (OS) and pine shell (PS) for Cu(II) removal in columns, using both traditional mathematical models and the Artificial Neural–Fuzzy Inference System (ANFIS) model to describe the results. Training of the model was performed using the output variable *C*/*C*_0_ and the input variables feed flow rate (A*), inlet concentration (B*), bed height (C*), and time (D*), employing 322 and 339 rows of data for the PS and OS biomaterials, respectively.

Validation of the ANFIS model employed 12 experiments for each type of bioadsorbent. The best fits of the experimental data were obtained with application of a Gaussian function, which is a method used to quantify the degree of association of a value to a fuzzy set [43], allowing analysis of the model uncertainty and inaccuracy, contributing to a more accurate and robust understanding of the behavior of the system.

The results showed that the ANFIS model was able to provide a more precise prediction of the experimental values, compared to traditional models, with *R*^2^ values of 0.9832 and 0.9868 for OS and PS, respectively. The ANFIS model provided estimated adsorption capacity (*Q*_e,est_) values of 2.51 and 3.24 mg g^−1^ for OS and PS, respectively, which were similar to the experimental adsorption capacity (*Q*_e,exp_) values of 2.52 and 3.26 mg g^−1^ for OS and PS (Table 2), respectively [43].

Although machine learning models can be applied for adsorption columns, there is discussion about their “black box” nature, which is a term used for conclusions or decisions made by artificial intelligence, without providing explanations that can be understood, because they are not visible to the user [180]. The mechanisms and factors that enable machine learning models to describe the adsorption phenomena are not known. Therefore, it is impossible to arrive at any type of physical-chemical interpretation of the models. However, the potential of artificial intelligence technology and its rapid advancement in various scientific fields suggest that AI-based models could soon become a viable solution for adsorption column modeling involving more complex systems.

## 9. Future Prospects for Use of Depleted Lignocellulosic Bioadsorbents

**Figure 11:**
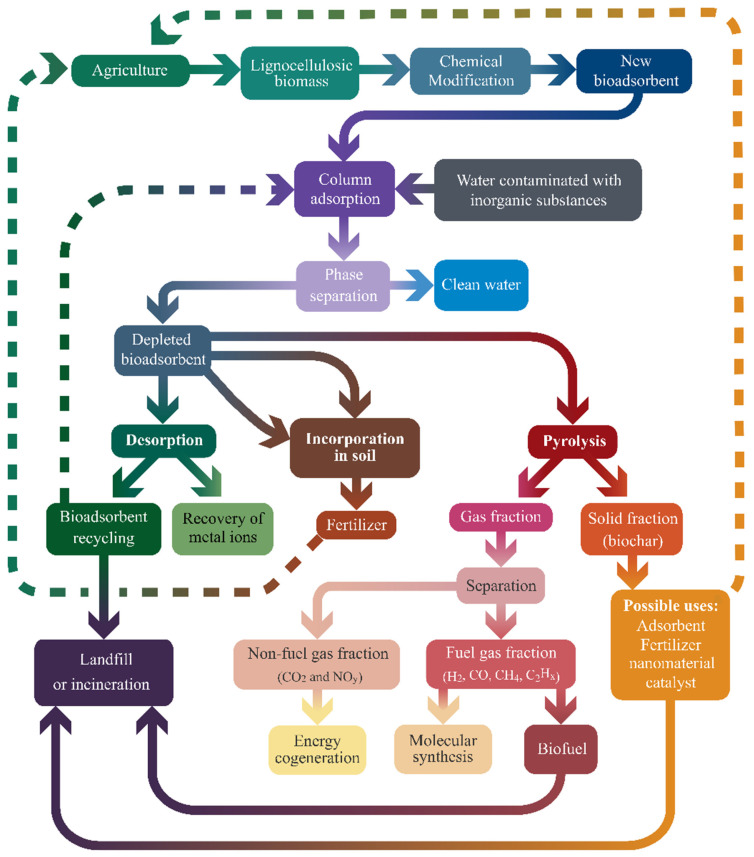
Flowchart for use and destination of lignocellulosic adsorbents. Adapted from Badescu et al. [181].

Although there is still a long way to go until the commercial application of bioadsorbents based on lignocellulosic materials is established, it is evident from the above discussion that this is a very promising approach for the treatment of water, regardless of the nature of the target pollutant (organic or inorganic) [105]. Figure 11 presents a flowchart of the production and use of bioadsorbents, highlighting possible recovery routes for these biomaterials that could potentialize their commercial use in water treatment and, when depleted, their use in other profitable applications, in line with the bio-based circular economy concept.

### 9.1. Bioadsorbent Regeneration and Reuse

Regeneration (desorption) and reuse (re-adsorption) in successive adsorption operation cycles is one of the most common ways proposed in the literature to improve the performance and service life of bioadsorbents, as well as their economic viability. Table 2 presents the main collected data for desorption and reuse of lignocellulosic adsorbents in columns, using eluents (desorption solutions) to recover the adsorption capacity of the biomaterials. Calculations of adsorption efficiency (*E*_ads_), desorption efficiency (*E*_des_), and re-adsorption efficiency (*E*_re-ads_) for adsorption–desorption–re-adsorption cycles can be found in the Supplementary Materials. Adsorption–desorption–re-adsorption cycles are carried out to evaluate the regeneration capacity of bioadsorbents, applying mass balance equations to understand the behavior of the bioadsorbent throughout the operation cycles. Ideally, the eluent used should provide high desorption efficiency, without compromising the bioadsorbent re-adsorption efficiency, avoiding degradation of the active sites on the surface.

The desorption method may involve the use of liquid eluents containing acids such as HCl [75], HNO_3_ [61,63], CH_3_COOH [75,113], and H_2_SO_4_ [56,63,87], or bases such as NaOH [63,77,94,99], as well as complexing agents such as EDTA, Na_2_EDTA [32,62,64], and (poly)amines (Table 2). The liquid-to-solid ratio of the desorption operation should be selected to recover as much bioadsorbent as possible, using the smallest volume of desorption solution, taking into consideration environmental and economic issues [181]. Otherwise, it is not possible to remove dilute toxic species from the contaminated water and transfer them to the desorption solution in a more concentrated form that facilitates their treatment or disposal.

Estimating the overall efficiency of the desorption operation is crucial for the process, since the ability to use the bioadsorbent in a greater number of adsorption–desorption–re-adsorption cycles extends its service life and reduces replacement costs. Furthermore, the recovery of contaminants that may have potential economic value or be useful in other applications is important for the economic viability of the process, requiring optimization of the concentration of toxic species transferred from contaminated water to the desorption eluent after the adsorption and desorption steps [181]. Recepoglu et al. [87] suggested a method for lithium recovery by its adsorption in columns, using phosphorylated hazelnut shell waste (FHS) as a bio-ecological adsorbent. The columns packed with 1.5 cm of FHS presented a removal capacity of 22.29 mg g^−1^ (3.21 mmol g^−1^) and an effective use of the bed of 81.2% (Table 2). Five regeneration cycles were performed, achieving approximately 100% recovery by desorption [87] (Table 2). These results highlight the potential for sustainable and cost-effective lithium extraction in continuous adsorption by a chemically modified lignocellulosic bioadsorbent.

The toxicity of non-desorbed pollutants may make it unfeasible to use the partially desorbed bioadsorbent in subsequent cycles [181], or increase the complexity of their disposal, since toxic solid waste requires specific procedures and monitoring, according to the environmental legislation of the country [4].

A limitation of the process is that the efficiency of pollutant removal from water gradually decreases with regeneration of the adsorbent and its use in successive adsorption–desorption cycles [181]. It is mainly the weaker adsorbent–adsorbate interactions that are affected, while stronger bonds remain intact, although the contact with desorption eluents may irreversibly degrade some of the active surface sites, reducing the adsorption capacity of the bioadsorbent [181]. Higher costs are a major concern in the regeneration of fixed-bed columns packed with activated carbon for water treatment, as these expenses can represent up to 75% of the total operation and maintenance costs of the process [26]. Regeneration does not eliminate the need to dispose of depleted bioadsorbents, in addition to generating a concentrated effluent that must be correctly handled [4]. Therefore, it is important to consider these aspects when selecting the desorption agent to be used in a technically, economically, and environmentally viable process.

### 9.2. Depleted Bioadsorbents: Disposal Concerns

None of the articles compiled in this review reported procedures for the final disposal of the depleted bioadsorbents. After exhaustion of the adsorption capacity, bioadsorbents are usually sent to appropriate landfills, or are incinerated [181,182], which must be carefully controlled to avoid creating negative environmental impacts [5,183]. Hence, it is necessary to evaluate and classify the contaminated biomaterials, according to the technical standards defined by relevant environmental legislation. In Brazil, the NBR 10004/2004 standard (https://www.abntcatalogo.com.br/, accessed on 26 March 2025), established by the Brazilian Association of Technical Norms, based on the North American Technical Procedure Code of Federal Regulations, determines that solid waste can be classified into three categories: (I) hazardous; (II-A) non-hazardous/non-inert; and (II-B) non-hazardous/inert [184].

At the end of their service life, depleted bioadsorbents composed of cellulose, hemicelluloses, and lignin are classified as non-hazardous/non-inert waste, mainly due to their biodegradability and combustibility [184]. However, depending on the toxicity and content of toxic contaminants, detected using standard leaching tests, this pre-established classification may change significantly [150]. When toxic pollutants are detected at levels exceeding the maximum permissible concentration limits, the depleted bioadsorbents are classified as toxic waste, so they must be sent to landfills for their proper disposal, with adequate control to prevent the leachate from reaching the soil, surface waters, and groundwater [184].

### 9.3. Incineration and Pyrolysis Evaluation

Controlled incineration is another option for the disposal of bioadsorbents, but pyrolysis emits compounds such as CO, CO_2_, CH_4_, CH_3_OH, C_2_H_4_, and SO_2_, among others, to the atmosphere, contributing to global warming and degradation of the ozone layer. Furthermore, the emissions from incineration may also include inorganic contaminants in the form of aerosols, requiring special attention and adequate control to avoid the release of toxic and harmful compounds into the environment [182].

The emission of carbon dioxide from the incineration of non-chemically modified bioadsorbents has no direct impact on global warming (carbon-neutral footprint), since the carbon emitted is already a component of the biogeochemical cycle of carbon, so the depleted lignocellulosic bioadsorbent can be used as a source of renewable fuel [181]. If bioadsorbents are chemically modified with ligands from non-renewable resources, the emissions will not be carbon-neutral. Inorganic contaminants released during incineration are mainly in the form of particulate matter, which can be removed by physical separation techniques such as bag filters and electrostatic precipitators [185]. For gaseous components, such as mercury, it may be necessary to add washing steps using wet scrubbers, or dry washing by the injection of activated carbon into the flue gas stream [185].

Depleted bioadsorbents can also be subjected to pyrolytic treatment, consisting of thermal degradation in an oxygen-poor environment, at moderate temperatures (200–800 °C). During this process, the organic fraction of the lignocellulosic biomass is thermally decomposed into biochar, bio-oil, and biogas [186]. The biochar has several possible applications, including as an adsorbent, catalyst, precursor in nanoparticle synthesis, and additive for the manufacture of metal alloys, as well as use in soil amendment [181]. The gaseous fraction can be divided into non-combustible gas (CO_2_ and NO_x_) and combustible gas (H_2_, CO, CH_4_, and C_2_H_x_), with potential for use in biofuel production [186]. In addition to generating bioproducts of economic value, biomass pyrolysis can be applied to any type of depleted bioadsorbent, even those containing toxic inorganic contaminants, which will remain immobilized in the biochar structure [24]. Despite the high equipment costs and concerns about the generation of both flue gas and contaminated biochar, biomass pyrolysis is a process that is economically viable and easily adaptable for use on a large scale [181].

Thermogravimetric analysis (TGA) is a well-known technique that can be used to investigate the thermal stability of lignocellulosic biomass and its thermal decomposition profile. When coupled to mass spectrometry, it can be used to investigate the formation of volatile compounds and identify their chemical structures for the purposes of environmental control and energy production. TGA can also be used to elucidate the combustion properties of the lignocellulosic biomass, providing criteria to determine whether incineration would be an appropriate option. Blázquez et al. [187] used TGA to obtain the thermal decomposition profile of a bioadsorbent derived from pine cone shell (PCS) after its saturation with Cu(II) ions. It was found that the presence of Cu(II) ions adsorbed on the PCS did not alter the thermal behavior of the biomaterial, compared to the raw PCS. Furthermore, the amount of Cu(II) present in the residual ash (biochar) was practically the same as that adsorbed by the PCS, indicating that pyrolysis of the PCS saturated with Cu(II) did not result in the release of Cu(II) in the form of volatile compounds. It was concluded that thermal treatment of PCS loaded with Cu(II) was a way to produce renewable energy, without emitting volatile metal-containing toxic compounds to the atmosphere.

### 9.4. Second Uses of Depleted Bioadsorbents as Fertilizers and Catalysts

When not classified as hazardous waste, depleted lignocellulosic bioadsorbents can be applied as fertilizers. This is due to their chemical composition, since they contain essential elements for plant growth, including macronutrients (N, P, K, Ca, Mg, and S) and micronutrients (B, Cl, Cu, Fe, Mn, and Mo), and present high biodegradability [5]. In addition to in-depth knowledge of the chemical composition of the bioadsorbent, the chemical composition of the effluent treated by adsorption must also be evaluated, considering the presence of toxic compounds, so that the depleted bioadsorbent does not become a source of soil pollution. When safely performed, the application of bioadsorbents as fertilizers could be a sustainable low-cost option for the agricultural sector.

The use of bioadsorbents as catalytic supports can be advantageous in the field of heterogeneous catalysis, since adsorption employing partially depleted bioadsorbents is difficult to control/monitor, which can result in partially treated effluents [188]. Partially depleted bioadsorbents loaded with “smart” metal ions can be used in catalytic reactions, offering a second life for these materials [188]. A recent study explored the potential of a bioadsorbent derived from chemically modified cellulose for the removal of As(V) and Cu(II) from water [151], and as a Cu(II)-containing catalyst for the conversion of aryl boronic acids to phenol derivatives [188]. These results highlight the versatility and reusability of bioadsorbents in the fields of heterogeneous catalysis and fine chemistry, to produce value-added products.

## 10. Future Perspectives

Some of the most important issues in adsorption involving lignocellulosic materials are as follows:

1. Early identification of which type of contaminant will be removed, the existence of competing contaminants, and the operating mode (batch or continuous) and conditions necessary to optimize its removal (including flow rate, volume to be treated, contaminant concentration, and permissible limits according to legislation) are essential criteria for defining the synthesis route, before carrying out any chemical modification.
2. The type of chemical modification and ligand(s) introduced into the structure of the lignocellulosic material should be based on the Williams–Irving series, considering the ionic character, electronegativity, and hydrated ionic radius of the metal ion, to ensure that the bioadsorbent is suitable for removing the target pollutant, in the presence of competing contaminants.
3. The cost-effectiveness of the bioadsorbent must be evaluated using a preliminary life-cycle analysis and a business model (TRL > 6) to ensure an efficient and economically viable product/process.
4. Comparison must be made between activated carbon and lignocellulosic bioadsorbents with specific functionalization for the removal of target pollutants, since these materials compete for similar applications.
5. Regeneration studies must not only evaluate different eluent solutions, from technical, economic, and environmental perspectives, but also consider the effects on the pollutant removal efficiency and the service life of the bioadsorbent.
6. Multicomponent column adsorption data modeling is still scarce in the literature, especially for systems containing three or more components. Due to the high computational cost, the use of emerging tools such as artificial neural networks and machine learning should be considered.
7. Tests in relevant environments and mini-pilot plants are essential to validate the business model and the life cycle analysis.
8. Environmentally safe disposal of depleted bioadsorbents and/or their second use is another crucial topic that should not be neglected, considering the concepts of a bio-based circular economy.

## 11. Conclusions

Analysis of the key aspects of the treatment of water and industrial effluents containing inorganic pollutants, using fixed-bed columns packed with lignocellulosic adsorbents, showed that few works have advanced to the technology readiness level (TRL) ≥6, corresponding to the mini-pilot or pilot scales in a relevant environment. Most of the reported studies applied bioadsorbents in fixed-bed columns, with monocomponent solutions, while few works have advanced to the use of fixed-bed columns with multicomponent solutions or effluents. This shows that more efforts are needed by researchers to overcome the limitations of bench-scale studies and the bottleneck of testing technologies on a larger scale, from grams to tens of grams. Although chemical modifications can improve the adsorptive properties of lignocellulosic materials, leading to more specific, selective, efficient, and regenerable bioadsorbents, the scaling-up of synthesis routes developed in the laboratory is one of the main bottlenecks to be overcome to achieve TRL > 6.

The different adsorption mechanisms evaluated in this review showed the complexity of competitive adsorption in multicomponent systems. The complexity of the mathematical modeling of adsorption columns in multicomponent systems proved to be another bottleneck for researchers, because the models are difficult to implement and the results are difficult to interpret. Therefore, strategies using more simplified models, as well as other tools such as artificial neural networks and artificial intelligence, are strongly recommended to accelerate the development of new continuous adsorption technologies based on bioadsorbents. The disposal and potential reuse of depleted bioadsorbents is another bottleneck. The viability of technologies is directly related to the principles of the bio-based circular economy and sustainable practices in the treatment of water and industrial effluents. Therefore, bioadsorbents that cannot be disposed of at low cost and have no second use are probably technologies with low profitability and will not be adopted by the market.

Another recommendation is to optimize the column operating conditions, considering not only the adsorption capacities of saturated biomaterials, but also the mass transfer zone and the effective use of the bed. The extent of the mass transfer zone influences the slope of the breakthrough curve, which, in an industrial unit where columns are installed in series, may require a greater number of columns. Only one study has evaluated the use of columns in series to improve bioadsorbent performance, which would obviously have a critical impact on the treatment cost.

Finally, the maturity of continuous adsorption column technologies must be accompanied by life cycle analysis, considering the long-term environmental and social benefits of using bioadsorbents. Although substantial progress has been made, it is clear that the developed technologies need to be tested on a larger scale, using configurations more similar to those of industrial units, so that they can achieve higher TRLs and receive investments that ensure progress until they become commercially viable, avoiding their abandonment.

## Figures and Tables

**Table 3:** Column dimensions and characteristics of the beds used in the most relevant studies that employed lignocellulosic biomasses as adsorbents in fixed-bed columns in recent years (from 2012 to 2025).

*T*/°C	Column Dimensions		Characteristics of the Bed			Reference
	*H*/cm	*D*/cm	*P*/μm	*H*_b_/cm	*w*/g	
-	1.5	30.0	355	10, 15, 20	-	[86]
25	0.7	12	-	1.0, 1.5, 2.0	-	[87]
22	20	1.2	<200	0.5, 0.7, 1.0	0.15, 0.22, 0.33	[88]
-	90	54	-	60	3550	[89]
25	10	1	500	3.1	-	[90]
25	51	3	<210	3.0, 7.0, 15.0	0	[91]
-	20	1.00	-	5.0, 10.0, 15.0	0.24, 0.44, 0.70	[92]
-	20	1	<500	5.0, 6.5, 8.0	-	[93]
30	30	1.3	200–400	3.0, 6.0, 9.0	0.93, 1.86, 2.64	[94]
30–80	15	4.1	100–1220	0.61, 3, 6.5, 10, 12.4	-	[70]
33–76	-	-	1000	1.6, 3.0, 6.0, 10.0, 11.5	-	[95]
29	10	3.0	<600	11.3, 20.9, 32.1	2.0, 4.0, 6.0	[51]
-	43	2	<300	1, 2, 3	-	[65]
25	10	1.00	<150	3.8	-	[67]
-	15	1.4	2000–3000	3.0, 6.0, 9.0	-	[77]
-	30	2.4	850	2, 3, 4	-	[96]
-	30	3	250–350	3, 5, 7	-	[49]
-	30	1.6	250–350	4, 8,10	-	[50]
-	25	1	250–400	2	1.03	[45]
30–35	20	1	149	-	0.5	[62]
-	20	2.54	<150	-	0.5	[73]
25	50	1.0	1000–3000	5.1, 6.4, 9.2	4.0, 5.5, 8.0	[97]
-	10	1.0	710–1000	2.5, 5.0, 7.5, 10.0	0.75, 1.50, 2.25, 3.00	[98]
-	20	1.00	-	-	0.5	[71]
-	20	1.00	<150	12.1	1.0	[64]
28–30	25	6.25	-	-	200	[99]
25	10	1.00	-	3.1	0.5	[61]
-	30	2.00	100–500	7.0	3	[63]
-	50	1.8	290–350	4, 6, 10	-	[59]
-	20.3	2	<2000	-	5	[46]
25	35	2.5	2580	24	24	[57]
-	24	3.00	-	21.0	-	[100]
-	1.5	-	128	2.5	1.8	[101]
-	23	1.5	420, 960, 1500	-	18	[102]
-	20	1	<520	5.0, 8.0, 10.0	2.10, 2.75, 3.20	[53]
-	-	-	<63	2.0, 3.0, 4.0	0.44, 0.66, 0.88	[58]
25	1.5	23	<1000	3.8, 11.5	5.0, 15.0	[42]
17	50	1.60	594	28.0	7.15	[69]
30	-	1.10	<150	5.0, 10.0, 15.0	-	[32]
-	28	0.8	500–1000	5, 7, 10, 15	0.5, 0.6, 0.75, 1.0	[103]
25	12	0.15	88–149	2.5, 5.0, 7.5	-	[104]
25	10	1.0	<250	3.20	0.5	[105]
-	20	1.00	<150	-	0.5	[68]
30	45	2	-	5.0, 10, 15	-	[106]
	30	1	-	2.5, 5.0, 7.5, 10	-	[107]
-	-	1	<350	2.5, 5.0, 10	-	[108]
-	25	4	-	2, 4, 6	4, 8, 12	[80]
-	20	1.00	500–100	-	-	[66]
-	50	1	1000–3000	-	5.5	[109]
-	-	0.6	150–300	-	0.5	[110]
25	-	-	<1000	5.0, 15.0	4.4, 13.4	[43]
-	16	1	1000–2000	4, 6, 12, 16	1.24, 1.99, 4.07, 5.09	[78]
-	100	22.00	425–600	9.5, 21, 31	5, 10, 15	[56]
-	30	1.00	<500	5.0, 6.5, 8.0	1.5, 1.92, 2.25	[75]
-	100	7.5	500–1500	66	350	[111]
-	23	1.5	<1000	14.3, 4.4	15, 5	[47]
30	5	2.5	244–355	2, 4, 5	0.120, 0.2341, 0.3426	[74]
-	27	3.42	500–1000	21.0–23.0	40.0	[37]
-	25	0.55	-	15	2.2	[112]
25	30	1	217–324.7	7, 14, 21	2, 4, 8	[113]
-	30	150	<500	150	75,000	[79]
-	15	1.00	500–1000	1.0, 3.0, 5.0	0.28, 0.84, 1.39	[114]
-	150	2.5, 5.0	710–1180	28.0, 15.0	47.5, 25.5	[52]
25	23	1.5	<1000	-	5	[83]
25	30	2.2	<210	2.0	4.0	[115]
-	9.5	1.00	-	-	2	[82]
25	10	2.54	<840	3	4.5	[44]
15	-	1.00	200–250	5.0, 10.0, 15.0	-	[116]
-	100, 160	62.00	200–250	-	480	[48]
-	20	1.00	<150	-	2	[60]
30	80	25.40	500–1400	20.0	972	[41]
20	10	1	250–500	6.7	0.5	[39]
-	55	1.70	1000–2000	50	25	[54]
-	15	1.5	-	7.0	0.7	[81]
-	30	2	300–500	1.5, 2.0	1.5, 2.0	[55]
-	30	1.78	<840	8.0, 15.0	-	[117]
-	25	1.14	250–500	-	1	[118]
25	23	1.50	<1000	4.4	5	[119]
-	20	1	75–150	-	0.5	[120]
25	23	1.5	<1000	4.41, 13.4	15, 5	[121]
23	29	2.20	300–500	1.4, 2.2, 2.9	2, 3, 4	[38]

*T*: temperature, *H*: column height, *D*: diameter, *P*: particle size, *H*_b_: column bed height, *w*: weight.

**Table 4:** Fitting parameters of the Bohart–Adams, Thomas, and Yoon–Nelson models expressed in terms of a generalized logistic equation.

Model	Logistic Equation Parameter		Reference
	*X*	*Y*	[15]
Bohart–Adams	$k_{BA}N_{0}L/u$	$k_{BA}C_{0}$	[11]
Thomas	$k_{Th}q_{0}M/Q$	$k_{Th}C_{0}$	[12]
Yoon–Nelson	$k_{YN}\tau$	$k_{YN}$	[13]

## Data Availability

Data are contained within the article and Supplementary Materials.

## Supplementary Materials

The following supporting information can be downloaded at https://www.mdpi.com/article/10.3390/polym17070953/s1. Figure S1: Schematic representation of the control volume adopted for a fixed-bed column adsorption modeling [130]; Table S1: Dimensionless parameters used in the HSDM; Table S2: Dimensionless parameters used in the LDF model; Table S3: Multicomponent adsorption isotherms models with nonlinear equations; Table S4: Monocomponent column adsorption models in their linear forms; Table S5: Properties of divalent cations used to predict the affinity order for the surface active sites of bioadsorbents. References [189,190,191,192,193] are cited in the Supplementary Materials.

## Abbreviations

The following abbreviations are used in this manuscript:

$A^{\prime }$	Area of the “desorption curve peak” (min)
*A*, *B*	Fitting parameters (min^−2^)
ANN	Artificial neural network
ANFIS	Artificial neural–fuzzy inference system
AIC	Akaike Information Criterion
BDST	Bed Depth Service Time
*C*_0_	Initial adsorbate concentration (mmol L^−1^ or mg L^−1^)
*C*	Column effluent concentration at time *t* (mmol L^−1^ or mg L^−1^)
*C*_e_	Equilibrium concentration (mmol L^−1^ or mg L^−1^)
*D*	Adsorption column diameter (cm)
$D_{ax}$	Axial dispersion coefficient (cm^2^ min^−1^)
*E*_ads_	Adsorption efficiency (%)
*E*_des_	Desorption efficiency (%)
*E*_re-ads_	Re-adsorption efficiency (%)
GA	Genetic algorithm
*H*_b_	Column bed height (cm)
*H*_unb_	Unused bed height (cm)
HSAB	Hard and Soft Acids and Bases
HSDM	Homogeneous Surface Diffusion Model
IAST	Ideal Adsorbed Solution Theory
*k*_BA_	Bohart–Adams rate constant (L mg^−1^ min^−1^ or L mmol^−1^ min^−1^)
*K*_sp_	Solubility product constant
*k*_Th_	Thomas rate constant (L mg^−1^ min^−1^ or L mmol^−1^ min^−1^)
*k*_YN_	Yoon–Nelson rate constant (min^−1^)
*JCR*	Journal citation reports
*L*	Bed depth (cm)
LDF	Linear Driving Force
*M*	Mass of the adsorbent in the column (mg or g)
*N*_0_	Adsorption capacity of the adsorbent per unit volume of the bed (mg L^−1^ or mmol L^−1^)
NC	Number of citations
*MTZ*	Mass transfer zone (cm)
*P*	Particle size (μm)
p*K*_f_	Complex formation constants
PSO	Particle swarm optimization
*q*_0_	Maximum adsorption capacity of the adsorbent (mg g^−1^ or mmol g^−1^)
*Q*	Volumetric flow rate (L min^−1^)
*Q*_e_	Solid loading per unit weight of adsorbent at equilibrium condition (mg g^−1^ or mmol g^−1^)
*Q*_e,est_	Estimated adsorption capacity (mg g^−1^ or mmol g^−1^)
*Q*_e,exp_	Experimental adsorption capacity (mg g^−1^ or mmol g^−1^)
*Q*_mono_	Solid loading per unit weight of adsorbent in a monocomponent system (mg g^−1^ or mmol g^−1^)
*Q*_multi_	Solid loading per unit weight of adsorbent in a multicomponent system (mg g^−1^ or mmol g^−1^)
RAST	Real Adsorbed Solution Theory
*R*^2^	Coefficient of determination
*R*^2^_adj_	Adjusted coefficient of determination
*T*	Temperature (ºC)
*t*	Time (min)
*t*_b_	Breakthrough time (min)
$t_{c}^{\prime }$	Center of the “desorption curve peak” (min)
*t*_s_	Saturation time (min)
TRL	Technology readiness level
*u*	Linear velocity of the fluid through the column (m s^−1^ or cm min^−1^)
*w*	Weight of adsorbent (mg or g)
*Z*	Distance along column length (cm)
$Z_{0}^{\prime }$	Offset (equal to $\frac{C_{e}}{C_{0}}\;$(1.0))
*Z*_e_	Effective use of the bed (%)
$w^{\prime }$	Half width of the “desorption curve peak”(min)
α	Fitting parameter for InOrdinatio equation
*β*	External mass transfer coefficient (min^−1^)
*β*_L_	Stability constant
*ƞ*	Absolute hardness
*χ*^2^	Chi-square
*χ*^2^_red_	Reduced chi-square
ε	Bed porosity
$\varepsilon_{b}$	Empty fraction of the column
$v_{F}$	Interstitial velocity (cm min^−1^)
*ρ*_p_	Particle density (g mL^−1^)

## References

[1] Ballesteros L.F., Michelin M., Vicente A.A., Teixeira J.A., Cerqueira M.A. (2018). Lignocellulosic Materials and Their Use in Bio-Based Packaging.

[2] O’Connell D.W., Birkinshaw C., O’Dwyer T.F. (2008). Heavy metal adsorbents prepared from the modification of cellulose: A review. Bioresour. Technol..

[3] Fomina M., Gadd G.M. (2014). Biosorption: Current perspectives on concept, definition and application. Bioresour. Technol..

[4] Maia L.C., Soares L.C., Gurgel L.V.A. (2021). A review on the use of lignocellulosic materials for arsenic adsorption. J. Environ. Manag..

[5] Reddy D.H.K., Vijayaraghavan K., Kim J.A., Yun Y.S. (2017). Valorisation of post-sorption materials: Opportunities, strategies, and challenges. Adv. Coll. Int. Sci..

[6] Thirunavukkarasu A., Nithya R., Sivashankar R. (2021). Continuous fixed-bed biosorption process: A review. Chem. Eng. J. Adv..

[7] Thirunavukkarasu O.S., Viraraghavan T., Subramanian K.S., Chaalal O., Islam M.R. (2005). Arsenic removal in drinking water—Impacts and novel removal technologies. Energy Sources.

[8] Patel H. (2019). Fixed-bed column adsorption study: A comprehensive review. Appl. Water Sci..

[9] Ruthven D.M. (1984). Principles of Adsorption and Adsorption Processes.

[10] Gu T. (2015). Mathematical Modeling and Scale-Up of Liquid Chromatography: With Application Examples.

[11] Bohart G.S., Adams E.Q. (1918). Some aspects of the behavior of charcoal with respect to chlorine.1. J. Am. Chem. Soc..

[12] Thomas H.C. (1944). Heterogeneous ion exchange in a flowing system. J. Am. Chem. Soc..

[13] Yoon Y.H., Nelson J.H. (1984). Application of gas adsorption kinetics. I. A theoretical model for respirator cartridge service life. Am. In. Hyg. Assoc. J..

[14] Wolborska A. (1989). Adsorption on activated carbon of *p*-nitrophenol from aqueous solution. Water Res..

[15] Chu K.H. (2020). Breakthrough curve analysis by simplistic models of fixed bed adsorption: In defense of the century-old Bohart-Adams model. Chem. Eng. J..

[16] Shafeeyan M.S., Daud W.M.A.W., Shamiri A. (2014). A review of mathematical modeling of fixed-bed columns for carbon dioxide adsorption. Chem. Eng. Res. Des..

[17] Chu K.H. (2010). Fixed bed sorption: Setting the record straight on the Bohart-Adams and Thomas models. J. Hazard. Mater..

[18] Pagani R.N., Kovaleski J.L., Resende L.M. (2015). Methodi Ordinatio: A proposed methodology to select and rank relevant scientific papers encompassing the impact factor, number of citation, and year of publication. Scientometrics.

[19] Bilal M., Shah J.A., Ashfaq T., Gardazi S.M.H., Tahir A.A., Pervez A., Haroon H., Mahmood Q. (2013). Waste biomass adsorbents for copper removal from industrial wastewater—A review. J. Hazard. Mater..

[20] Fu F., Wang Q. (2011). Removal of heavy metal ions from wastewaters: A review. J. Environ. Manag..

[21] Ferreira Junior J.E.G., Maia L.C., dos Santos G.R., Soares L.C., Gurgel L.V.A. (2023). An affordable bioadsorbent system to treat arsenic-contaminated drinking water in the developing world: Prototyping and economic assessment. J. Environ. Chem. Eng..

[22] Afroze S., Sen T.K. (2018). A Review on heavy metal ions and dye adsorption from water by agricultural solid waste adsorbents. Water Air Soil Pollut..

[23] Myers A.L., Prausnitz J.M. (1965). Thermodynamics of mixed-gas adsorption. AIChE J..

[24] Wang Y., Lü J., Feng D., Guo S., Li J. (2020). A Biosorption-pyrolysis process for removal of Pb from aqueous solution and subsequent immobilization of Pb in the char. Water.

[25] Weber W.J., Smith E.H. (1986). Activated carbon adsorption: The state of the art. Stud. Environ. Sci..

[26] Çeçen F., Aktaş Ö. (2011). Activated Carbon for Water and Wastewater Treatment: Integration of Adsorption and Biological Treatment.

[27] Malik D.S., Jain C.K., Yadav A.K. (2018). Heavy metal removal by fixed-bed column—A review. ChemBioEng Rev..

[28] Dai Y., Sun Q., Wang W., Lu L., Liu M., Li J., Yang S., Sun Y., Zhang K., Xu J. (2018). Utilizations of agricultural waste as adsorbent for the removal of contaminants: A review. Chemosphere.

[29] Agarwal A., Upadhyay U., Sreedhar I., Singh S.A., Patel C.M. (2020). A review on valorization of biomass in heavy metal removal from wastewater. J. Water Process Eng..

[30] Aksu Z. (2005). Application of biosorption for the removal of organic pollutants: A review. Process Biochem..

[31] Ambaye T.G., Vaccari M., van Hullebusch E.D., Amrane A., Rtimi S. (2020). Mechanisms and adsorption capacities of biochar for the removal of organic and inorganic pollutants from industrial wastewater. Int. J. Environ. Sci. Technol..

[32] Wang F., Yu J., Zhang Z., Xu Y., Chi R.-A. (2018). An amino-functionalized ramie stalk-based adsorbent for highly effective Cu^2+^ removal from water: Adsorption performance and mechanism. Process Saf. Environ. Prot..

[33] Singh S., Kumar V., Datta S., Dhanjal D.S., Sharma K., Samuel J., Singh J. (2020). Current advancement and future prospect of biosorbents for bioremediation. Sci. Total Environ..

[34] Nassar M.M. (2006). Adsorption of Fe^+3^ and Mn^+2^ from groundwater onto maize cobs using batch adsorber and fixed bed column. Sep. Sci. Technol..

[35] Taka A.L., Klink M.J., Mbianda X.Y., Naidoo E.B. (2021). Chitosan nanocomposites for water treatment by fixed-bed continuous flow column adsorption: A review. Carbohydr. Polym..

[36] Ali I. (2013). Water treatment by adsorption columns: Evaluation at ground level. Sep. Purif. Rev..

[37] Amirnia S., Ray M.B., Margaritis A. (2016). Copper ion removal by *Acer saccharum* leaves in a regenerable continuous-flow column. Chem. Eng. J..

[38] Chen S., Yue Q., Gao B., Li Q., Xu X., Fu K. (2012). Adsorption of hexavalent chromium from aqueous solution by modified corn stalk: A fixed-bed column study. Bioresour. Technol..

[39] Escudero C., Poch J., Villaescusa I. (2013). Modelling of breakthrough curves of single and binary mixtures of Cu(II), Cd(II), Ni(II) and Pb(II) sorption onto grape stalks waste. Chem. Eng. J..

[40] Wang L., Xu Z., Fu Y., Chen Y., Pan Z., Wang R., Tan Z. (2018). Comparative analysis on adsorption properties and mechanisms of nitrate and phosphate by modified corn stalks. RSC Adv..

[41] Acheampong M.A., Pakshirajan K., Annachhatre A.P., Lens P.N.L. (2013). Removal of Cu(II) by biosorption onto coconut shell in fixed-bed column systems. J. Ind. Eng. Chem..

[42] Blazquez G., Calero M., Trujillo C., Martín-Lara Á., Ronda A. (2018). Binary biosorption of Cu(II)-Pb(II) mixtures onto pine nuts shell in batch and packed bed systems. Environ. Eng. Manag. J..

[43] Calero M., Ianez-Rodriguez I., Perez A., Martín-Lara M.A., Blázquez G. (2018). Neural fuzzy modelization of copper removal from water by biosorption in fixed-bed columns using olive stone and pinion shell. Bioresour. Technol..

[44] Chao H.-P., Chang C.-C., Nieva A. (2014). Biosorption of heavy metals on *Citrus maxima* peel, passion fruit shell, and sugarcane bagasse in a fixed-bed column. J. Ind. Eng. Chem..

[45] Li J., Ma J., Guo Q., Zhang S., Han H., Zhang S., Han R. (2020). Adsorption of hexavalent chromium using modified walnut shell from solution. Water Sci. Technol..

[46] Liu J., Hu C., Huang Q. (2019). Adsorption of Cu^2+^, Pb^2+^, and Cd^2+^ onto oiltea shell from water. Bioresour. Technol..

[47] Martín-Lara M.A., Blázquez G., Calero M., Almendros A.I., Ronda A. (2016). Binary biosorption of copper and lead onto pine cone shell in batch reactors and in fixed bed columns. Int. J. Miner. Process..

[48] Raulino G.S., Vidal C.B., Lima A.C., Melo D.Q., Oliveira J.T., Nascimento R.F. (2014). Treatment influence on green coconut shells for removal of metal ions: Pilot-scale fixed-bed column. Environ. Technol..

[49] Yahya M.D., Abubakar H., Obayomi K.S., Iyaka Y.A., Suleiman B. (2020). Simultaneous and continuous biosorption of Cr and Cu (II) ions from industrial tannery effluent using almond shell in a fixed bed column. Results Eng..

[50] Yahya M.D., Aliyu A.S., Obayomi K.S., Olugbenga A.G., Abdullahi U.B., Arellano-Garcia H. (2020). Column adsorption study for the removal of chromium and manganese ions from electroplating wastewater using cashew nutshell adsorbent. Cogent Eng..

[51] Rajeswari M., Agrawal P., Rao N.N., Sharma A., Hiremath L., Tippareddy K.S., Shivandappa (2021). Modelling and efficiency assessment of the up flow fixed bed process packed with *Moringa oleifera* for continuous Cd(II) removal from drinking water. J. Mol. Struct..

[52] Asif Z., Chen Z. (2015). Removal of arsenic from drinking water using rice husk. Appl. Water Sci..

[53] Basu M., Guha A.K., Ray L. (2019). Adsorption of lead on lentil husk in fixed bed column bioreactor. Bioresour. Technol..

[54] Iqbal M., Saeed A., Edyvean R.G.J. (2013). Bioremoval of antimony(III) from contaminated water using several plant wastes: Optimization of batch and dynamic flow conditions for sorption by green bean husk (*Vigna radiata*). Chem. Eng. J..

[55] Sharma R., Singh B. (2013). Removal of Ni (II) ions from aqueous solutions using modified rice straw in a fixed bed column. Bioresour. Technol..

[56] Abdolali A., Ngo H.H., Guo W., Zhou J.L., Zhang J., Liang S., Chang S.W., Nguyen D.D., Liu Y. (2017). Application of a breakthrough biosorbent for removing heavy metals from synthetic and real wastewaters in a lab-scale continuous fixed-bed column. Bioresour. Technol..

[57] Feisther V.A., Scherer Filho J., Hackbarth F.V., Mayer D.A., de Souza A.A.U., de Souza S.M.A.G.U. (2019). Raw leaves and leaf residues from the extraction of essential oils as biosorbents for metal removal. J. Environ. Chem. Eng..

[58] Hymavathi D., Prabhakar G. (2019). Modeling of cobalt and lead adsorption by *Ficus benghalenesis* L. in a fixed bed column. Chem. Eng. Commun..

[59] Mitra T., Das S.K. (2019). Cr(VI) removal from aqueous solution using *Psidium guajava* leaves as green adsorbent: Column studies. Appl. Water Sci..

[60] Yu J., Feng L., Cai X., Wang L., Chi R. (2014). Adsorption of Cu^2+^, Cd^2+^ and Zn^2+^ in a modified leaf fixed-bed column: Competition and kinetics. Environ. Earth Sci..

[61] de Almeida F.T.R., Elias M.M.C., Xavier A.L.P., Ferreira G.M.D., Silva I.A., Filgueiras J.G., de Azevedo E.R., da Silva L.H.M., Gil L.F., Gurgel L.V.A. (2019). Synthesis and application of sugarcane bagasse cellulose mixed esters. Part II: Removal of Co^2+^ and Ni^2+^ from single spiked aqueous solutions in batch and continuous mode. J. Colloid Interface Sci..

[62] Chen J., Yu J., Wang F., Tang J., Zhang Y., Xu Y., Chi R. (2017). Selective adsorption and recycle of Cu^2+^ from aqueous solution by modified sugarcane bagasse under dynamic condition. Environ. Sci. Pollut. Res..

[63] Hoang M.T., Pham T.D., Nguyen V.T., Nguyen M.K., Pham T.T., Van der Bruggen B. (2019). Removal and recovery of lead from wastewater using an integrated system of adsorption and crystallization. J. Clean. Prod..

[64] Yu J., Xiong W., Sun Q., Zhu J., Chi R., Zhang Y. (2019). Separation of Pb^2+^ from Mg^2+^ by modified sugarcane bagasse under batch and column conditions: Effect of initial concentration ratio. Arab. J. Chem..

[65] Kausar A., Bhatti H.N., Iqbal M. (2021). Kinetics and equilibrium of radioactive metal adsorption onto sugarcane bagasse waste: Comparison of batch and column adsorption modes. Z. Phys. Chem..

[66] Rico I.L.R., Carrazana R.J.C., Karna N.K., Iáñez-Rodríguez I., de Hoces M.C. (2018). Modeling the mass transfer in biosorption of Cr(VI) y Ni(II) by natural sugarcane bagasse. Appl. Water Sci..

[67] Rodrigues J.A.V., Martins L.R., Furtado L.M., Xavier A.L.P., de Almeida F.T.R., Moreira A.L.S.L., Melo T.M.S., Gil L.F., Gurgel L.V.A. (2020). Oxidized renewable materials for the removal of cobalt(II) and copper(II) from aqueous solution using in batch and fixed-Bed column adsorption. Adv. Polym. Technol..

[68] Tang J., Xi J., Yu J., Chi R., Chen J. (2018). Novel combined method of biosorption and chemical precipitation for recovery of Pb^2+^ from wastewater. Environ. Sci. Pollut. Res..

[69] Vera L.M., Bermejo D., Uguña M.F., Garcia N., Flores M., González E. (2018). Fixed bed column modeling of lead(II) and cadmium(II) ions biosorption on sugarcane bagasse. Environ. Eng. Res..

[70] Villabona-Ortíz A., Tejada-Tovar C., González-Delgado Á.D. (2022). Elimination of chromium(VI) and nickel(II) ions in a packed column using oil palm bagasse and yam peels. Water.

[71] Xiong W., Zhang J., Yu J., Chi R. (2019). Competitive adsorption behavior and mechanism for Pb^2+^ selective removal from aqueous solution on phosphoric acid modified sugarcane bagasse fixed-bed column. Process Saf. Environ. Prot..

[72] Teodoro F.S., Soares L.C., Filgueiras J.G., de Azevedo E.R., Patiño-Agudelo Á.J., Adarme O.F.H., da Silva L.H.M., Gurgel L.V.A. (2022). Batch and continuous adsorption of Cu(II) and Zn(II) ions from aqueous solution on bi-functionalized sugarcane-based biosorbent. Environ. Sci. Pollut. Res..

[73] Xu Y.-L., Song S.-Y., Chen J.-D., Chi R.-A., Yu J.-X. (2019). Simultaneous recovery of Cu^2+^ and Pb^2+^ from metallurgical wastewater by two tandem columns fixed respectively with tetraethylenepentamine and phosphoric acid modified bagasse. J. Taiwan Inst. Chem. Eng..

[74] Manikandan N.A., Alemu A.K., Goswami L., Pakshirajan K., Pugazhenthi G. (2016). Waste *Litchi* peels for Cr(VI) removal from synthetic wastewater in batch and continuous systems: Sorbent characterization, regeneration and reuse study. J. Environ. Eng..

[75] Basu M., Guha A.K., Ray L. (2017). Adsorption of cadmium ions by cucumber peel in continuous mode. Int. J. Environ. Sci. Technol..

[76] Chatterjee A., Schiewer S. (2014). Effect of competing cations (Pb, Cd, Zn, and Ca) in fixed-bed column biosorption and desorption from citrus peels. Water Air Soil Pollut..

[77] Mora B.P., Bertoni F.A., Mangiameli M.F., González J.C., Bellú S.E. (2020). Batch and fixed-bed column studies of selenite removal from contaminated water by orange peel-based sorbent. Water Sci. Eng..

[78] Romero-Cano L.A., González-Gutiérrez L.V., Baldenegro-Pérez L.A., Carrasco-Marín F. (2017). Grapefruit peels as biosorbent: Characterization and use in batch and fixed bed column for Cu(II) uptake from wastewater. J. Chem. Technol. Biotechnol..

[79] Rosales E., Meijide J., Tavares T., Pazos M., Sanromán M.A. (2016). Grapefruit peelings as a promising biosorbent for the removal of leather dyes and hexavalent chromium. Process Saf. Environ. Prot..

[80] Du Z., Zheng T., Wang P. (2018). Experimental and modelling studies on fixed bed adsorption for Cu(II) removal from aqueous solution by carboxyl modified jute fiber. Powder Technol..

[81] Tofan L., Teodosiu C., Paduraru C., Wenkert R. (2013). Cobalt(II) removal from aqueous solutions by natural hemp fibers: Batch and fixed-bed column studies. Appl. Surf. Sci..

[82] Vidal C.B., Melo D.Q., Raulino G.S.C., da Luz A.D., da Luz C., Nascimento R.F. (2015). Multielement adsorption of metal ions using Tururi fibers (*Manicaria saccifera*): Experiments, mathematical modeling and numerical simulation. Desalin. Water Treat..

[83] Ronda A., Calero M., Blázquez G., Pérez A., Martín-Lara M.A. (2015). Optimization of the use of a biosorbent to remove heavy metals: Regeneration and reuse of exhausted biosorbent. J. Taiwan Inst. Chem. Eng..

[84] Jain C.K., Malik D.S., Yadav A.K. (2016). Applicability of plant based biosorbents in the removal of heavy metals: A review. Environ. Process..

[85] Hasan S.H., Ranjan D., Talat M. (2010). Agro-industrial waste ’wheat bran’ for the biosorptive remediation of selenium through continuous up-flow fixed-bed column. J. Hazard. Mater..

[86] Gawas A.S., Sutar P.R., Gokhale J.S. (2025). Biosorption of vanadium(V) and chromium(VI) using abscised coconut leaves powder: Equilibrium and continuous packed bed column studies. Environ. Chem. Ecotoxicol..

[87] Recepoglu Y.K., Arar Ö., Yuksel A. (2024). Breakthrough curve analysis of phosphorylated hazelnut shell waste in column operation for continuous harvesting of lithium from water. J. Chromatogr. A.

[88] Amar M.B., Mallek M., Valverde A., Monclus H., Myers T.G., Salvado V., Cabrera-Codony A. (2024). Competitive heavy metal adsorption on pinecone shells: Mathematical modelling of fixed-bed column and surface interaction insights. Sci. Total Environ..

[89] Gagnon-Poirier S., Zagury G.J., Robert T., Courcelles B. (2023). Pilot-scale removal of metals from iron-rich contaminated groundwater using phosphorylated lignocellulosic fibers. Water Air Soil Pollut..

[90] Elias M.M.C., Soares L.C., Maia L.C., Taylor J.G., Adarme O.F.H., Ferreira G.M.D., de Azevedo E.R., de Siervo A., da Silva L.H.M., Gurgel L.V.A. (2023). Batch and continuous adsorption of Cd(II) and Pb(II) on polycarboxylated sugarcane bagasse. J. Water Process Eng..

[91] Safardastgerdi M., Ardejani F.D., Mahmoodi N.M. (2023). Lignocellulosic biomass functionalized with EDTA dianhydride for removing Cu(II) and dye from wastewater: Batch and fixed-bed column adsorption. Min. Eng..

[92] Wang F., Hu X., Tang C., Liu C., Zhu Z. (2023). Phosphate-functionalized ramie stalk adsorbent for efficient removal of Zn^2+^ from water: Adsorption performance, mechanism, and fixed-bed column treatment of real wastewater. Environ. Sci. Pollut. Res..

[93] Basu M., Guha A.K. (2023). Separation of lead from aqueous phase by cucumber peel in column bioreactor: A phenomenon of interaction between biological and chemical system and its ecological importance. J. Environ. Manag..

[94] Dovi E., Aryee A.A., Kani A.N., Mpatani F.M., Li J., Qu L., Han R. (2022). High-capacity amino-functionalized walnut shell for efficient removal of toxic hexavalent chromium ions in batch and column mode. J. Environ. Chem. Eng..

[95] Tejada-Tovar C., Villabona-Ortíz A., Ortega-Toro R. (2021). Batch and packed bed column study for the removal of Cr(VI) and Ni(II) using agro-industrial wastes. Appl. Sci..

[96] Nithya K., Sathish A., Kumar P.S. (2020). Packed bed column optimization and modeling studies for removal of chromium ions using chemically modified *Lantana camara* adsorbent. J. Water Process Eng..

[97] Petrella A., Spasiano D., Rizzi V., Cosma P., Race M., De Vietro N. (2019). Thermodynamic and kinetic investigation of heavy metals sorption in packed bed columns by recycled lignocellulosic materials from olive oil production. Chem. Eng. Commun..

[98] Priyantha N., Kotabewatta P.A. (2019). Biosorption of heavy metal ions on peel of Artocarpus nobilis fruit: 1—Ni(II) sorption under static and dynamic conditions. Appl. Water Sci..

[99] Cherdchoo W., Nithettham S., Charoenpanich J. (2019). Removal of Cr(VI) from synthetic wastewater by adsorption onto coffee ground and mixed waste tea. Chemosphere.

[100] Gondhalekar S.C., Shukla S.R. (2019). Enhanced adsorption performance of oxidised coconut coir for removal of Cd(II) ions by multi-column arrangement in series. Environ. Sci. Pollut. Res..

[101] Arim A.L., Quina M.J., Gando-Ferreira L.M. (2019). Uptake of trivalent chromium from aqueous solutions by xanthate pine bark: Characterization, batch and column studies. Process Saf. Environ. Prot..

[102] Fernández-González R., Martín-Lara M.Á., Blázquez G., Pérez A., Calero M. (2019). Recovering metals from aqueous solutions by biosorption onto hydrolyzed olive cake. Water.

[103] Jangde V., Umathe P., Antony P.S., Shinde V., Pakade Y. (2018). Fixed-bed column dynamics of xanthate-modified apple pomace for removal of Pb(II). Int. J. Environ. Sci. Technol..

[104] Arim A.L., Neves K., Quina M.J., Gando-Ferreira L.M. (2018). Experimental and mathematical modelling of Cr(III) sorption in fixed-bed column using modified pine bark. J. Clean. Prod..

[105] Xavier A.L.P., Adarme O.F.H., Furtado L.M., Ferreira G.M.D., da Silva L.H.M., Gil L.F., Gurgel L.V.A. (2018). Modeling adsorption of copper(II), cobalt(II) and nickel(II) metal ions from aqueous solution onto a new carboxylated sugarcane bagasse. Part II: Optimization of monocomponent fixed-bed column adsorption. J. Colloid Interface Sci..

[106] Shanmugaprakash M., Venkatachalam S., Rajendran K., Pugazhendhi A. (2018). Biosorptive removal of Zn(II) ions by *Pongamia* oil cake (*Pongamia pinnata*) in batch and fixed-bed column studies using response surface methodology and artificial neural network. J. Environ. Manag..

[107] Mathangi J.B., Kalavathy M.H. (2018). Study of mathematical models for the removal of Ni^2+^ from aqueous solutions using *Citrullus lanatus* rind, an agro-based waste. Water Environ. J..

[108] Zaidi N.A.H.M., Lim L.B.L., Usman A. (2018). Enhancing adsorption of Pb(II) from aqueous solution by NaOH and EDTA modified *Artocarpus odoratissimus* leaves. J. Environ. Chem. Eng..

[109] Petrella A., Spasiano D., Acquafredda P., De Vietro N., Ranieri E., Cosma P., Rizzi V., Petruzzelli V., Petruzzelli D. (2018). Heavy metals retention (Pb(II), Cd(II), Ni(II)) from single and multimetal solutions by natural biosorbents from the olive oil milling operations. Process Saf. Environ. Prot..

[110] Khan U., Rao R.A.K. (2017). A high activity adsorbent of chemically modified *Cucurbita moschata* (a novel adsorbent) for the removal of Cu(II) and Ni(II) from aqueous solution: Synthesis, characterization and metal removal efficiency. Process Saf. Environ. Prot..

[111] Castro L., Blazquez M.L., González F., Muñoz J.A., Ballester A. (2017). Biosorption of Zn(II) from industrial effluents using sugar beet pulp and *F. vesiculosus*: From laboratory tests to a pilot approach. Sci. Total Environ..

[112] Borna M.O., Pirsaheb M., Niri M.V., Mashizie R.K., Kakavandi B., Zare M.R., Asadi A. (2016). Batch and column studies for the adsorption of chromium(VI) on low-cost *Hibiscus Cannabinus* kenaf, a green adsorbent. J. Taiwan Inst. Chem. Eng..

[113] Davila-Guzman N.E., Cerino-Córdova F.J., Loredo-Cancino M., Rangel-Mendez J.R., Gómez-González R., Soto-Regalado E. (2016). Studies of Adsorption of Heavy Metals onto Spent Coffee Ground: Equilibrium, Regeneration, and Dynamic Performance in a Fixed-Bed Column. Int. J. Chem. Eng..

[114] Lakshmipathy R., Sarada N.C. (2015). A fixed bed column study for the removal of Pb^2+^ ions by watermelon rind. Environ. Sci. Water Res. Technol..

[115] Cheraghi E., Ameri E., Moheb A. (2015). Adsorption of cadmium ions from aqueous solutions using sesame as a low-cost biosorbent: Kinetics and equilibrium studies. Int. J. Environ. Sci. Technol..

[116] Oguz E., Ersoy M. (2014). Biosorption of cobalt(II) with sunflower biomass from aqueous solutions in a fixed bed column and neural networks modelling. Ecotoxicol. Environ. Saf..

[117] Cruz-Olivares J., Pérez-Alonso C., Barrera-Díaz C., Ureña-Nuñez F., Chaparro-Mercado M.C., Bilyeu B. (2013). Modeling of lead (II) biosorption by residue of allspice in a fixed-bed column. Chem. Eng. J..

[118] Khan M.A., Ngabura M., Choong T.S., Masood H., Chuah L.A. (2012). Biosorption and desorption of nickel on oil cake: Batch and column studies. Bioresour. Technol..

[119] Martín-Lara M.A., Blázquez G., Ronda A., Rodríguez I.L., Calero M. (2012). Multiple biosorption–desorption cycles in a fixed-bed column for Pb(II) removal by acid-treated olive stone. J. Ind. Eng. Chem..

[120] Hang Y., Shan J., Jun-Xia Y., Ru-An C. (2019). Effects of Ca^2+^ initial concentration on Cu^2+^ selective adsorption from aqueous solution by modified sugarcane bagasse under batch and column condition. Int. J. Environ. Anal. Chem..

[121] Ronda A., Martín-Lara M.A., Dionisio E., Blázquez G., Calero M. (2013). Effect of lead in biosorption of copper by almond shell. J. Taiwan Inst. Chem. Eng..

[122] Zhang Y., Zhao M., Cheng Q., Wang C., Li H., Han X., Fan Z., Su G., Pan D., Li Z. (2021). Research progress of adsorption and removal of heavy metals by chitosan and its derivatives: A review. Chemosphere.

[123] Zhang S., Ding J., Tian D., Kang R., Zhao X., Chang M., Yang W., Xie H., Lu M. (2023). As(V) removal from aqueous environments using quaternary ammonium modified ZIF-8/chitosan composite adsorbent. Appl. Surf. Sci..

[124] Sun R.C. (2010). Cereal Straw as a Resource for Sustainable Biomaterials and Biofuels: Chemistry, Extractives, Lignins, Hemicelluloses and Cellulose.

[125] Bukva M., Soares L.C., Maia L.C., Costa C.S.D., Gurgel L.V.A. (2023). A review on the design and application of bi-functionalized adsorbents to remove different pollutants from water. J. Water Process Eng..

[126] Hoang A.T., Nižetić S., Cheng C.K., Luque R., Thomas S., Banh T.L., Pham V.V., Nguyen X.P. (2022). Heavy metal removal by biomass-derived carbon nanotubes as a greener environmental remediation: A comprehensive review. Chemosphere.

[127] Afroze S., Sen T.K., Ang H.M. (2016). Adsorption removal of zinc (II) from aqueous phase by raw and base modified *Eucalyptus sheathiana bark*: Kinetics, mechanism and equilibrium study. Process Saf. Environ. Prot..

[128] Ahmed M.J., Hameed B.H. (2018). Removal of emerging pharmaceutical contaminants by adsorption in a fixed-bed column: A review. Ecotoxicol. Environ. Saf..

[129] da Costa T.B., da Silva M.G.C., Vieira M.G.A. (2020). Recovery of rare-earth metals from aqueous solutions by bio/adsorption using non-conventional materials: A review with recent studies and promising approaches in column applications. J. Rare Earths.

[130] Worch E. (2012). Adsorption Technology in Water Treatment: Fundamentals, Processes, and Modeling.

[131] Mankins J.C. (1995). Technology Readiness Levels—A White Paper.

[132] Taty-Costodes V.C., Fauduet H., Porte C., Ho Y. (2005). Removal of lead(II) ions from synthetic and real effluents using immobilized *Pinus sylvestris* sawdust: Adsorption on a fixed-bed column. J. Hazard. Mater..

[133] Wolborska A., Pustelnik P. (1996). A simplified method for determination of the break-through time of an adsorbent layer. Water Res..

[134] González-López M.E., Laureano-Anzaldo C.M., Pérez-Fonseca A.A., Arellano M., Robledo-Ortíz J.R. (2020). Chemically modified polysaccharides for hexavalent chromium adsorption. Sep. Purif. Rev..

[135] Wang J., Guo X. (2020). Adsorption kinetic models: Physical meanings, applications, and solving methods. J. Hazard. Mater..

[136] El-Khaiary M.I., Malash G.F. (2011). Common data analysis errors in batch adsorption studies. Hydrometallurgy.

[137] Osmari T.A., Gallon R., Schwaab M., Barbosa-Coutinho E., Severo J.B., Pinto J.C. (2013). Statistical analysis of linear and non-linear regression for the estimation of adsorption isotherm parameters. Adsorpt. Sci. Technol..

[138] McCuen R.H., Surbeck C.Q. (2008). An alternative to specious linearization of environmental models. Water Res..

[139] Pagnanelli F., Esposito A., Vegliò F. (2002). Multi-metallic modelling for biosorption of binary systems. Water Res..

[140] Majd M.M., Kordzadeh-Kermani V., Ghalandari V., Askari A., Sillanpaa M. (2022). Adsorption isotherm models: A comprehensive and systematic review (2010–2020). Sci. Total Environ..

[141] Neris J.B., Luzardo F.H.M., da Silva E.G.P., Velasco F.G. (2019). Evaluation of adsorption processes of metal ions in multi-element aqueous systems by lignocellulosic adsorbents applying different isotherms: A critical review. Chem. Eng. J..

[142] Mohan S., Sreelakshmi G. (2008). Fixed bed column study for heavy metal removal using phosphate treated rice husk. J. Hazard. Mater..

[143] Xie Y., Xiong W., Yu J., Tang J., Chi R. (2018). Recovery of copper from metallurgical sludge by combined method of acid leaching and biosorption. Process Saf. Environ. Prot..

[144] Kleinübing S.J., Guibal E., da Silva E.A., da Silva M.G.C. (2012). Copper and nickel competitive biosorption simulation from single and binary systems by *Sargassum filipendula*. Chem. Eng. J..

[145] Holmberg J.P. (2006). Competitive Adsorption and Displacement Behaviour of Heavy Metals on Peat. Master’s Thesis.

[146] Irving H., Williams R.J.P. (1948). Order of stability of metal complexes. Nature.

[147] Varadwaj P.R., Varadwaj A., Jin B. (2015). Ligand(s)-to-metal charge transfer as a factor controlling the equilibrium constants of late first-row transition metal complexes: Revealing the Irving-Williams thermodynamical series. Phys. Chem. Chem. Phys..

[148] Xu H., Xu D.C., Wang Y. (2017). Natural indices for the chemical hardness/softness of metal cations and ligands. ACS Omega.

[149] Pearson R.G. (1963). Hard and soft acids and bases. J. Am. Chem. Soc..

[150] Parr R.G., Pearson R.G. (1986). Absolute hardness: Companion parameter to absolute electronegativity. J. Am. Chem. Soc..

[151] Pereira A.R., Soares L.C., Teodoro F.S., Elias M.M.C., Ferreira G.M.D., Savedra R.M.L., Siqueira M.F., Martineau-Corcos C., da Silva L.H.M., Prim D. (2020). Aminated cellulose as a versatile adsorbent for batch removal of As(V) and Cu(II) from mono- and multicomponent aqueous solutions. J. Colloid Interface Sci..

[152] Goel J., Kadirvelu K., Rajagopal C. (2016). Competitive sorption of Cu(II), Pb(II) and Hg(II) ions from aqueous solution using coconut shell-based activated carbon. Adsorp. Sci. Technol..

[153] Nightingale E.R. (2002). Phenomenological theory of ion solvation. Effective radii of hydrated ions. J. Phys. Chem..

[154] Speight J.G. (2005). Lange’s Handbook of Chemistry.

[155] Huheey J.E., Keiter E.A., Keiter R.L. (1993). Inorganic Chemistry: Principles of Structure and Reactivity.

[156] Ballesteros-Plata D., Zhang Y., Rodríguez-Castellón E., Vincent T., Guibal E. (2023). Brown algal residue for the recovery of metal ions—Application to La(III), Cd(II), and Ni(II) sorption. Adv. Sustain. Syst..

[157] Yu J., Wang L., Chi R., Zhang Y., Xu Z., Guo J. (2013). Adsorption of Pb^2+^, Cd^2+^, Cu^2+^, and Zn^2+^ from aqueous solution by modified sugarcane bagasse. Res. Chem. Intermed..

[158] Inglezakis V.J., Fyrillas M.M., Park J. (2019). Variable diffusivity homogeneous surface diffusion model and analysis of merits and fallacies of simplified adsorption kinetics equations. J. Hazard. Mater..

[159] Amrutha, Jeppu G., Girish C.R., Prabhu B., Mayer K. (2023). Multi-component adsorption isotherms: Review and modeling studies. Environ. Process..

[160] Butler J.A.V., Ockrent C. (1930). Studies in electrocapillarity. III. J. Phys. Chem..

[161] Bellot J.C., Condoret J.S. (1993). Modelling of liquid chromatography equilibria. Process Biochem..

[162] Teodoro F.S., Adarme O.F.H., Gil L.F., Gurgel L.V.A. (2017). Synthesis and application of a new carboxylated cellulose derivative. Part II: Removal of Co^2+^, Cu^2+^ and Ni^2+^ from bicomponent spiked aqueous solution. J. Colloid Interface Sci..

[163] Soetaredjo F.E., Kurniawan A., Ki O.L., Ismadji S. (2013). Incorporation of selectivity factor in modeling binary component adsorption isotherms for heavy metals-biomass system. Chem. Eng. J..

[164] Schay G.J., Fejes F.P., Szethmary J. (1957). Adsorption of gases and Gas Mixtures. Acta Chim. Acad. Sci. Hung..

[165] Ho Y.S., McKay G. (1999). Pseudo-second order model for sorption processes. Process Biochem..

[166] Fritz W., Schluender E.U. (1974). Simultaneous adsorption equilibria of organic solutes in dilute aqueous solutions on activated carbon. Chem. Eng. Sci..

[167] Sheindorf C., Rebhun M., Sheintuch M. (1981). A Freundlich-type multicomponent isotherm. J. Colloid Interface Sci..

[168] McKay G., Al Duri B. (1987). Simplified model for the equilibrium adsorption of dyes from mixtures using activated carbon. Chem. Eng. Process. Process Intensif..

[169] Ramos S.N.C., Xavier A.L.P., Teodoro F.S., Gil L.F., Gurgel L.V.A. (2016). Removal of cobalt(II), copper(II), and nickel(II) ions from aqueous solutions using phthalate-functionalized sugarcane bagasse: Mono- and multicomponent adsorption in batch mode. Ind. Crops Prod..

[170] Costa E., Sotelo J.L., Calleja G., Marrón C. (1981). Adsorption of binary and ternary hydrocarbon gas mixtures on activated carbon: Experimental determination and theoretical prediction of the ternary equilibrium data. AIChE J..

[171] Reynel-Avila H.E., Mendoza-Castillo D.I., Olumide A.A., Bonilla-Petriciolet A. (2016). A survey of multi-component sorption models for the competitive removal of heavy metal ions using bush mango and flamboyant biomasses. J. Mol. Liq..

[172] Cho B., Mun S., Lim C., Kang S.B., Cho C., Yun Y. (2022). Adsorption modeling of microcrystalline cellulose for pharmaceutical-based micropollutants. J. Hazard. Mater..

[173] Jin S., Cho B., Mun S., Kim S., Cho C. (2023). Investigation of the adsorption affinity of organic micropollutants on seaweed and its QSAR study. Environ. Res..

[174] Sun M., Wang X., Xiong R., Chen X., Zhai L., Wang S. (2023). High-performance biochar-loaded MgAl-layered double oxide adsorbents derived from sewage sludge towards nanoplastics removal: Mechanism elucidation and QSAR modeling. Sci. Total Environ..

[175] Pauletto P.S., Dotto G.L., Salau N.P.G. (2020). Optimal artificial neural network design for simultaneous modeling of multicomponent adsorption. J. Mol. Liq..

[176] Fagundez J.L.S., Salau N.P.G. (2022). Optimization-based artificial neural networks to fit the isotherm models parameters of aqueous-phase adsorption systems. Environ. Sci. Pollut. Res..

[177] Kennedy J., Eberhart R. Particle swarm optimization. Proceedings of the ICNN’95—International Conference on Neural Networks.

[178] Kramer O. (2017). Genetic Algorithm Essentials.

[179] Oguz E., Ersoy M. (2010). Removal of Cu^2+^ from aqueous solution by adsorption in a fixed bed column and Neural Network Modelling. Chem. Eng. J..

[180] Setzu M., Guidotti R., Monreale A., Turini F., Pedreschi D., Giannotti F. (2021). GLocalX—From local to global explanations of black box AI models. Artif. Intell..

[181] Bădescu I.S., Bulgariu D., Ahmad I., Bulgariu L. (2018). Valorisation possibilities of exhausted biosorbents loaded with metal ions—A review. J. Environ. Manag..

[182] Cherubini F., Bargigli S., Ulgiati S. (2009). Life cycle assessment (LCA) of waste management strategies: Landfilling, sorting plant and incineration. Energy.

[183] Chojnacka K. (2010). Biosorption and bioaccumulation—The prospects for practical applications. Environ. Int..

[184] (2004). Solid Waste—Classification.

[185] Daskalopoulos E., Badr O., Probert S.D. (1997). Economic and environmental evaluations of waste treatment and disposal technologies for municipal solid waste. Appl. Energy.

[186] Guedes R.E., Luna A.S., Torres A.R. (2018). Operating parameters for bio-oil production in biomass pyrolysis: A review. J. Anal. Appl. Pyrolysis.

[187] Blázquez G., Martín-Lara M.A., Dionisio-Ruiz E., Tenorio G., Calero M. (2012). Copper biosorption by pine cone shell and thermal decomposition study of the exhausted biosorbent. J. Ind. Eng. Chem..

[188] Torres F.G., Teodoro F.S., Gurgel L.V.A., Bourdreux F., Zayene O., Gaucher A., Gil L.F., Prim D. (2022). Application of raw and chemically modified biomasses for heterogeneous Cu-catalysed conversion of aryl boronic acids to phenols derivatives. Catalysts.

[189] Myers R.H., Montgomery D.C., Anderson-Cook C.M. (2016). Response Surface Methodology Process and Product Optimization Using Designed Experiments.

[190] Yang R.T. (1987). Gas Separation by Adsorption Processes.

[191] Sips R. (1948). On the Structure of a Catalyst Surface. J. Chem. Phys..

[192] Redlich O., Peterson D. L. (1959). A Useful Adsorption Isotherm. J. Phys. Chem..

[193] Hutchins R.A. (1973). New simplified design of activated carbon systems. Chem. Eng..

