# Supplementary material for: A Review on Advances in the Use of Raw and Modified Agricultural Lignocellulosic Residues in Mono- and Multicomponent Continuous Adsorption of Inorganic Pollutants for Upscaling Technologies
Source: Polymers (Basel). 2025 Mar 31;17(7):953. doi: 10.3390/polym17070953 (PMC11991513; doi:10.3390/polym17070953)
Supplement: Supplementary file 1 [file polymers-17-00953-s001.zip › polymers-3469365-supplementary.pdf]

Electronic Supplementary Material

A Review on Advances in the Use of Raw and Modified Agricultural Lignocellulosic Residues in Mono- and Multicomponent Continuous Adsorption of Inorganic Pollutants for Upscaling Technologies

Ricardo Silva Coelho [1,2], Liliane Catone Soares [1], Oscar Fernando Herrera Adarme [3], Luisa Cardoso Maia [1,2], Camila Stéfanne Dias Costa [1,2], Eric Guibal [4] and Leandro Vinícius Alves Gurgel [1,*]

[1] Group of Physical Organic Chemistry, Department of Chemistry, Institute of Exact and Biological Sciences, Federal University of Ouro Preto, Campus Universitário Morro do Cruzeiro, Rua Quatro, 786, Bauxita, Ouro Preto 35402-136, MG, Brazil; ricardo.coelho@aluno.ufop.edu.br (R.S.C.); liliane.catone@ufop.edu.br (L.C.S.); luisa.maia@ufop.edu.br (L.C.M.); camilasdcosta@gmail.com (C.S.D.C.)

[2] Environmental Engineering Graduate Program (ProAmb), School of Mines, Federal University of Ouro Preto, Campus Universitário Morro do Cruzeiro, Rua Nove, s/n, Bauxita, Ouro Preto 35402-163, MG, Brazil

[3] Faculdade de Engenharia Agrícola, Universidade Estadual de Campinas (Unicamp), Av. Cândido Rondon, 501, Campinas 13083-875, SP, Brazil; oscarf@unicamp.br (O.F.H.A.)

[4] Polymers Composites and Hybrids (PCH), IMT Mines Ales, 30100 Ales, France; eric.guibal@mines-ales.fr (E.G.)

*Correspondence: legurgel@ufop.edu.br (L.V.A.G.)

Web of Science search Equation:

ALL=((column OR continuous OR pack*) AND (sorption* AND capacity) AND (Stalk OR shell OR seed OR husk OR straw OR waste* OR bran OR leaves OR chaff OR hauls OR peel OR bagasse OR root OR sawdust OR fiber OR residue OR agricul*))

Link: https://www.webofscience.com/wos/woscc/summary/960be776-1854-4e74-b8e0-4a8050ff6794-699b147a/date-descending/1

The coefficient of determination

The coefficient of determination (R^2) is obtained from the residual sum of squared errors (*SSE*) and total sum of squares (SS_{total}) (Eq. (1)) [138]. *SSE* refers to the squared difference between the observed and predicted values (Eq. (2)), while SS_{total} is the squared difference between the experimental data and its mean value (Eq. (3)) [138].

$$R^2 = 1 - \frac{SSE}{SS_{\text{total}}} \qquad \text{Eq. (1)}$$

$$SSE = \sum_{i=1}^{n} (y_{i,\text{pred}} - y_{i,\text{exp}})^2 \qquad \text{Eq. (2)}$$

$$SS_{\text{total}} = \sum_{i=1}^{n} (y_{i,\text{exp}} - \bar{y})^2 \qquad \text{Eq. (3)}$$

Efficiency of adsorption

The efficiency of adsorption (E_{ads}) was calculated using Eq. (4) [61]:

$$E_{\text{ads}}/\% = \left(\frac{C_0 Q_{\text{cont}} t}{q_{\text{max}} w_{\text{ad}}}\right) \times 100 \qquad \text{Eq. (4)}$$

where C_0 (mg L^{-1}) is the initial contaminant concentration in solution, Q_{cont} (L min^{-1}) is contaminant flow rate, t (min) is the time, q_{max} (mg g^{-1}) is the maximum adsorption capacity of the contaminant determined for the adsorbent, w_{ad} (g) is the weight of the adsorbent.

Efficiency of desorption

The efficiency of desorption (E_{des}) was calculated using Eq. (5) [61]:

$$E_{des}/\% = \left(\frac{C_{e,M}V_{eluent}}{q_{max}w_{ad}'}\right) \times 100 \quad \text{Eq. (5)}$$

where $C_{e,M}$ (mg L^{-1}) is the contaminant concentration in the desorption solution at equilibrium, V_{eluent} (L) is the eluent solution volume, q_{max} (mg g^{-1}) is the maximum adsorption capacity of the contaminant determined for the adsorbent, w_{ad}' (g) is the weight of pure adsorbent in the weight of adsorbent loaded with the contaminant species ($w_{ad,cont}$). The value of w_{ad}' is calculated using Eq. (6) [61]:

$$w_{ad}'/g = \frac{w_{ad,cont}}{\left(\frac{q_{max}}{1000}\right) + 1} \quad \text{Eq. (6)}$$

Efficiency of re-adsorption

The efficiency of re-adsorption ($E_{re\text{-}ads}$) for one adsorption-desorption-re-adsorption cycle was calculated using Eq. (7) [61]:

$$E_{re-ads}/\% = \left(\frac{Q_{re-ads}}{Q_e}\right) \times 100 \quad \text{Eq. (7)}$$

where $Q_{\text{re-ads}}$ (mg g^{-1}) is the adsorption capacity of the adsorbent after the desorption process, Q_{e} (mg g^{-1}) is the adsorption capacity of the adsorbent before the desorption process. The value of $Q_{\text{re-ads}}$ can be calculated using Eq. (8) [61]:

$$Q_{\text{re-ads}}/(\text{mg g}^{-1}) = \frac{w'_{\text{cont}} + w''_{\text{cont}}}{w_{\text{ad}}} \qquad \text{Eq. (8)}$$

where w'_{cont} (mg) is weight of contaminant that was not desorbed from the adsorbent after desorption, w''_{cont} (mg) is the weight of contaminant adsorbed on the adsorbent after re-adsorption. The values of w'_{cont} and w''_{cont} can be calculated using Eq. (9) and Eq. (10) [61], respectively.

$$w'_{\text{cont}}/\text{ mg} = \left[w_{\text{ad,M}} - w_{\text{ad}}\right]\left(1 - \frac{E_{\text{des}}}{100}\right) \qquad \text{Eq. (9)}$$

$$w''_{\text{cont}}/\text{ mg} = (c_0 - c_{\text{e}})V \qquad \text{Eq. (10)}$$

where $w_{\text{ad,M}}$ and w_{ad} (mg) are the weight of adsorbent with and without the adsorbed contaminant species, respectively, E_{des} (%) is the efficiency of desorption, c_0 (mg L^{-1}) is the initial contaminant concentration, c_{e} (mg L^{-1}) is the equilibrium contaminant concentration, and V (L) is the solution volume.

Design of experiments (DOE) for column adsorption assays

Design of experiments (DOE) is defined as a branch of applied statistics that deals with planning, conducting, analyzing, and interpreting controlled tests to evaluate the factors (inputs/influence variables) that control the value of experimental outputs (response variables). In this sense, for the chosen experimental response, a correlation can be developed involving the significant factors [189]. Different types of DOEs, such as full,

fractional, or response surface methods can be applied to identify important multivariate interactions that may be missed when varying different factors univariately. Typically, different types of variables such as bed height, volume and porosity, cross-sectional area, inlet concentration, particle diameter, and inlet flow rate are used in the experimental design for adsorption column assays. However, the main variables used in the literature are inlet concentration, adsorbent mass, and spatial time, which is defined as the ratio of the column void volume and the applied inlet flow rate. An example of experimental conditions for column optimization design can be found in Xavier et al. [105] where, for example, batch adsorption data such as equilibrium time obtained from kinetic data and maximum adsorption capacity, adsorbent mass, and initial solute concentration obtained from equilibrium data, were used to estimate the initial inlet solute concentration at the center point of the central composite design. It is important to note that there is a relationship between initial and equilibrium solute concentration and the adsorbent mass for adsorption isotherms obtained by varying the initial solute concentration while keeping the adsorbent mass fixed or varying adsorbent mass while keeping the initial solute concentration fixed. This way, Eq. (11) is used to obtain the parameters for fixed-bed column operation, as follows:

$$C_0 = \frac{q_{\mathrm{max}} m_{\mathrm{ads}}}{t_{\mathrm{e}} \dot{V}} \qquad \text{Eq. (11)}$$

where C_0 (mg L^{-1} or mmol L^{-1}) is the initial inlet solute concentration, q_{max} (mg g^{-1} or mmol g^{-1}) is the maximum adsorption capacity of the adsorbent for the target solute species obtained in batch, m_{ads} (g) is the weight of adsorbent to be packed into the bed, t_e (min) is the equilibrium time obtained in batch, and $\dot{V}$ (L min^{-1}) is the inlet flow rate.

Differential mass balance equations for column modeling

The mass balance equations for column adsorption were obtained from Worch [130]. The control volume adopted to establish a differential mass balance using an infinite volume element ($\mathrm{d}V = A_\mathrm{R}\ \mathrm{d}_\mathrm{Z}$) [130], from a cylindrical column filled with adsorbent with cross-sectional area (A_R) and upward flow is shown in Figure S1.

Figure S1. Schematic representation of the control volume adopted for a fixed-bed column adsorption modeling [130].

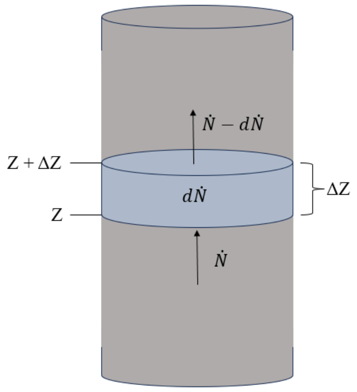

It is assumed that the amount of adsorbate that is adsorbed on the adsorbent or accumulated in the void fraction of the volume element must be equal to the difference between the input and output of the volume element. The transport of adsorbate occurs by advection and axial dispersion. This way, the overall mass balance can be given by Eq. (12) [130]:

$$\dot{N}_\mathrm{accu} + \dot{N}_\mathrm{ads} = \dot{N}_\mathrm{disp} + \dot{N}_\mathrm{adv} \qquad \text{Eq. (12)}$$

where $\dot{N}$ represents the change in the amount of adsorbate with time, and the subscripts indicate the processes of accumulation, adsorption, dispersion, and advection [130]. The

accumulation of an adsorbate species within the void fraction of the volume element, dV, is given by Eq. (13) [130]:

$$\dot{N}_{\mathrm{accu}} = \varepsilon_{\mathrm{b}}\, A_{\mathrm{R}} \partial Z\, \frac{\partial c_i(t, Z)}{\partial t} = \varepsilon_{\mathrm{b}} \partial V\, \frac{\partial c_i(t, Z)}{\partial t} \qquad \text{Eq. (13)}$$

where ε_{b} is the empty fraction of the column, Z is the distance along the length of the column (cm), c_i concentration of component i in the phase fluid (mmol L^{-1}), t is the time (min).

The adsorption of adsorbate on the adsorbent in the volume element can be written as shown in Eq. (14) [130]:

$$\dot{N}_{\mathrm{ads}} = \rho_{\mathrm{b}}\, A_{\mathrm{R}} \partial Z\, \frac{\partial \bar{q}(t, Z)}{\partial t} = \rho_{\mathrm{b}} \partial V\, \frac{\partial \bar{q}(t, Z)}{\partial t} \qquad \text{Eq. (14)}$$

where $\bar{q}$ is the mean adsorbent loading capacity (g g^{-1} or mmol g^{-1}), ρ_{P} is the particle density (g mL^{-1}). To describe advection (Eq. (15)) [130], it is necessary to consider the difference between the amount of adsorbate fed to and released by the volume element per unit of time. Appling differential form, the advection process can be written as Eq. (16).

$$\dot{N}_{\mathrm{adv}} = v_{\mathrm{F}}\, A_{\mathrm{R}}\, \partial c_i(t, Z) - \partial c_i(t, Z + dZ) \qquad \text{Eq. (15)}$$

$$\dot{N}_{\mathrm{adv}} = -v_{\mathrm{F}}\, A_{\mathrm{R}}\, \frac{\partial c_i(t, Z)}{\partial Z} dZ = -v_{\mathrm{F}}\, dV\, \frac{\partial c_i(t, Z)}{\partial Z} \qquad \text{Eq. (16)}$$

where v_{F} is the interstitial velocity (cm min^{-1}), c_i concentration of component i in the fluid phase (mmol L^{-1}), and A_R is the cross-sectional area. The Fick's first law allows describing the axial dispersion in the bed. The difference between input and output

caused by axial dispersion can be written as shown in Eq. (17) or at differential form shown in Eq. (18) [130].

$$\dot{N}_{\text{disp}} = D_{\text{ax}}\varepsilon_{\text{b}}A_{\text{R}}\left[\frac{\partial c_i(t,Z)}{\partial Z}\right]_{Z+dZ} - D_{\text{ax}}\varepsilon_{\text{b}}A_{\text{R}}\left[\frac{\partial c_i(t,Z)}{\partial Z}\right]_{Z} \quad \text{Eq. (17)}$$

$$\dot{N}_{\text{disp}} = D_{\text{ax}}\varepsilon_{\text{b}}A_{\text{R}}\frac{\partial^2 c_i(t,Z)}{\partial Z^2}dZ = D_{\text{ax}}\varepsilon_{\text{b}}dV\frac{\partial^2 c_i(t,Z)}{\partial Z^2} \quad \text{Eq. (18)}$$

where D_{ax} is the axial dispersion coefficient (cm^2 min^{-1}).

Substituting Eqs. (12), (13), (15), and (17) into Eq. (11) and rearranging the resulting equation, yields Eq. (19), which is the mass balance equation in its general form [130]:

$$v_{\text{F}}\frac{\partial c_i(t,Z)}{\partial Z} + \varepsilon_{\text{b}}\frac{\partial c_i(t,Z)}{\partial t} + \rho_{\text{b}}\frac{\partial \bar{q}(t,Z)}{\partial t} - D_{\text{ax}}\varepsilon_{\text{b}}\frac{\partial^2 c_i(t,Z)}{\partial Z^2} = 0 \quad \text{Eq. (19)}$$

The breakthrough curve models consider adsorption equilibrium and kinetics. Adsorption kinetics (third term of Eq. (19)) involves the mass transport of adsorbate from the bulk phase to the outer surface of the adsorbent (film diffusion), the transport of adsorbate from the film to the pores of the adsorbent (pore diffusion), and the transport of the adsorbate on the inner surface of the adsorbent (surface diffusion).

The third term of Eq. (19) represents the mass balance of the solid phase (adsorbent) which provides the adsorption rate equation for each *i* component. Typically, rather than being a simple algebraic expression, this term is more likely to represent a set of diffusion equations (mass transfer resistances) with their associated boundary conditions. Due to the complexity of modeling and interpreting the data, the models choose to consider only outer (film) and inner (pore and surface) diffusions [130]. In

most cases, inner diffusion is assumed to be dominated by surface diffusion, and possible additional mass transport by pore diffusion is accounted for the effective surface diffusion coefficient [130]. The HSDM and LDF models, for the third term of Eq. (19), are the most frequently applied and will be presented in more detail [130].

Homogeneous surface diffusion model (HSDM)

The surface diffusion approach assumes that mass transfer takes place along the inner surface of the adsorbent particle. The solid phase concentration gradient within the particle acts as the driving force for transport of adsorbate species. In the surface diffusion model, the adsorbent is considered a homogeneous medium. This model is therefore called the homogeneous surface diffusion model (HSDM) [130]. The HSDM combines the mass balance equation (Eq. (19)), including terms for film diffusion, surface diffusion, and dispersion effect, with the latter term being able to be disregarded [130]. Neglecting the axial dispersion effect, Eq. (19) can be rewritten as shown in Eq. (20) [130]:

$$v_{\mathrm{F}} \frac{\partial c_i(t,Z)}{\partial Z} + \varepsilon_{\mathrm{b}} \frac{\partial c_i(t,Z)}{\partial t} + \rho_{\mathrm{b}} \frac{\partial \bar{q}(t,Z)}{\partial t} = 0 \qquad \text{Eq. (20)}$$

The third term of Eq. (20), representing the mass transfer rate from the fluid phase to the solid phase, can be replaced by the mass transfer equation in the film (Eq. (22)) [130]. Therefore, Eq. (21) can be rewritten as shown in Eq. (22) [130],

$$\frac{\partial \bar{q}(t,Z)}{\partial t} = \frac{K_{\mathrm{F}} a_{\mathrm{VR}}}{\rho_{\mathrm{b}}} (c - c_{\mathrm{s}}) \qquad \text{Eq. (21)}$$

$$v_{\mathrm{F}} \frac{\partial c_i(t,Z)}{\partial Z} + \varepsilon_{\mathrm{b}} \frac{\partial c_i(t,Z)}{\partial t} + \frac{K_{\mathrm{F}} a_{VR}}{\rho_{\mathrm{b}}} (c - c_{\mathrm{s}}) = 0 \qquad \text{Eq. (22)}$$

where K_{F} is the mass transfer coefficient in the film surrounding the adsorbent particles (cm min^{-1}), c and c_{s} are the adsorbate concentrations in bulk phase and external particle surface (mg L^{-1} or mmol L^{-1}), a_{VR} is the external surface area (cm^2) related to reactor

volume [130]. For spherical particles with radius r_p (cm), a_VR can be written as shown in Eq. (23) [130]. Substituting Eq. (23) into the Eq. (22) [130], yields Eq. (24).

$$a_\mathrm{VR} = \frac{3}{r_\mathrm{p}}(1 - \varepsilon_\mathrm{b}) \qquad \text{Eq. (23)}$$

$$v_\mathrm{F}\frac{\partial c_i(t,Z)}{\partial Z} + \varepsilon_\mathrm{b}\frac{\partial c_i(t,Z)}{\partial t} + \frac{3K_\mathrm{F}(1-\varepsilon_\mathrm{b})}{r_\mathrm{p}}(c - c_\mathrm{s}) = 0 \qquad \text{Eq. (24)}$$

On the other hand, the surface diffusion equation can be written according to Eq. (25) considering the constant diffusion coefficient [130],

$$\frac{\partial q}{dt} = D_\mathrm{s}\left(\frac{\partial^2 q}{\partial r^2} + \frac{2\,\partial q}{r\partial r}\right) \qquad \text{Eq. (25)}$$

where r is the radial coordinate (cm), D_s is the surface diffusion coefficient (cm^2 min^{-1}) and q is the mean adsorbent loading capacity (mg g^{-1} or mmol g^{-1}). The model also needs to consider the equilibrium condition (isotherm) that relates q to c_s on the external surface of the adsorbent particle (Eq. (26)) [130].

$$q(t, Z, r = r_\mathrm{p}) = f(c_\mathrm{s}(t, Z)) \qquad \text{Eq. (26)}$$

From the equation system formed by Eqs. (24), (25), and (26), it is possible to determine the dependent variables q, c_s, and c according to the initial and boundary conditions.

The initial and boundary conditions:

For Eq. (25) [130]:

$$q(t = 0, Z, r) = 0 \qquad \text{Eq. (27)}$$

$$\left[\frac{\partial q(t, Z, r)}{\partial r}\right]_{r=0} = 0 \qquad \text{Eq. (28)}$$

$$\left[\frac{\partial q(t, Z, r)}{\partial r}\right]_{r=r_\mathrm{p}} = \frac{K_\mathrm{F}}{\rho_\mathrm{p}\, D_\mathrm{s}}[c(t, Z) - c_\mathrm{s}(t, Z)] \qquad \text{Eq. (29)}$$

For Eq. (26) [130]:

$$c(t, Z = 0) = c_0 \qquad \text{Eq. (30)}$$

To facilitate its mathematical application, the terms of the equations are converted to their dimensionless forms. Table S1 presents the dimensionless parameters defined for applying the HSDM model [130].

Table S1. Dimensionless parameters used in the HSDM

Dimensionless parameter	Symbol	Definition
Dimensionless concentration	X	$X = \frac{C}{C_0}$
Dimensionless adsorbent loading capacity	Y	$Y = \frac{q}{q_0}$
Dimensionless radial coordinate (within the particle)	R	$R = \frac{r}{r_\mathrm{p}}$
Dimensionless axial coordinate (distance from adsorbent inlet)	S	$S = \frac{Z}{h}$
Solute distribution parameter	D_g	$D_\mathrm{g} = \frac{q_0\, m_\mathrm{A}}{C_0\, \varepsilon_\mathrm{b}\, V_\mathrm{R}} = \frac{q_0\, \rho_\mathrm{b}}{C_0\, \varepsilon_\mathrm{b}}$
Dimensionless time (throughput ratio)	T	$T = \frac{t}{t_\mathrm{b}^{id}} = \frac{t}{t_\mathrm{r}(D_\mathrm{g}+1)}$
Stanton number (transport rate ratio: film transfer/advection)	St^*	$St^* = \frac{K_\mathrm{F}\, t_\mathrm{r}\,(1-\varepsilon_\mathrm{b})}{\varepsilon_\mathrm{b} r_\mathrm{p}}$
Diffusion modulus (transport rate ratio: surface diffusion/advection)	E_d	$E_\mathrm{d} = \frac{D_\mathrm{s}\, D_\mathrm{g}\, t_\mathrm{r}}{r_\mathrm{p}^2}$
Biot number (transport rate ratio: film diffusion/surface diffusion)	Bi	$Bi = \frac{St^*}{E_\mathrm{d}} = \frac{K_\mathrm{F}\, r_\mathrm{p} C_0\,(1-\varepsilon_\mathrm{b})}{q_0\, \rho_\mathrm{b} D_\mathrm{s}}$

By using the dimensionless parameters, the HSDM equations can be written as follows [130]:

$$\frac{\partial X}{\partial S} + \frac{1}{(D_\mathrm{g}+1)}\frac{\partial X}{\partial T} + 3St^*(X - X_\mathrm{s}) = 0 \qquad \text{Eq. (31)}$$

$$\frac{\partial Y}{\partial T} = E_\mathrm{d}\frac{D_g+1}{D_\mathrm{g}} + \left(\frac{\partial^2 Y}{\partial R^2} + \frac{2}{R}\frac{\partial Y}{\partial R}\right) \qquad \text{Eq. (32)}$$

$$Y(T,S,R=1) = f(X_\mathrm{s}(T,S)) \qquad \text{Eq. (33)}$$

The initial and boundary conditions are [130]:

For Equation Eq. (30):

$$Y(T = 0, S, R) = 0 \quad \text{Eq. (34)}$$

$$\left[\frac{\partial Y(T, S, R)}{\partial R}\right]_{R=0} = 0 \quad \text{Eq. (35)}$$

$$\left[\frac{\partial Y(T, S, R)}{\partial R}\right]_{R=1} = Bi[X(T, S) - X_s(T, S)] \quad \text{Eq. (36)}$$

For Eq. (31):

$$X(T, S = 0) = 1 \quad \text{Eq. (37)}$$

Eq. (33) is the dimensionless form for any isotherm model, therefore, to solve the system of equations using the initial boundary conditions, numerical solution methods are used.

Linear driving force model

The Linear Driving Force (LDF) model is applied to describe in a more simplified way the mass transfer between phases through simpler mathematical applications [130]. The driving force is the change in adsorbate concentration on the surface of a particle and in the surrounding fluid. The LDF model is a further simplification of the HSDM [130]. The feature of the LDF model is a simplified description of the intraparticle diffusion phenomena. Instead of Fick's law, the mass transfer equation with driving force is used, facilitating the solution, which is an advantage in the case of complex multicomponent systems [130]. Considering the mass transfer by negligible axial dispersion and applying the general isotherm equation, the equations used in the LDF model are shown in Eqs. (38)-(43):

$$v_{\mathrm{F}} \frac{\partial c_i(t,Z)}{\partial Z} + \varepsilon_{\mathrm{b}} \frac{\partial c_i(t,Z)}{\partial t} + \rho_{\mathrm{b}} \frac{\partial \bar{q}(t,Z)}{\partial t} = 0 \qquad \text{Eq. (38)}$$

$$c(t = 0, Z) = c_0, \bar{q}(t = 0, Z) = 0 \qquad \text{Eq. (39)}$$

$$c(t, Z = 0) = c_0 \qquad \text{Eq. (40)}$$

$$\frac{\partial \bar{q}(t,Z)}{\partial t} = \frac{K_{\mathrm{F}} a_{\mathrm{VR}}}{\rho_{\mathrm{b}}} (c - c_{\mathrm{s}}) \qquad \text{Eq. (41)}$$

$$\frac{\partial \bar{q}(t,Z)}{\partial t} = K_{\mathrm{s}}^{*}(q_{\mathrm{s}} - \bar{q}) \qquad \text{Eq. (42)}$$

$$q_{\mathrm{s}} = f\ (c_{\mathrm{s}}, T) \qquad \text{Eq. (43)}$$

where K_{F} is the film mass transfer coefficient, a_{VR} is the area available for mass transfer related to the reactor volume, K_{s}^{*} is the intraparticle mass transfer coefficient, c_{s} and q_{s} are the adsorbate concentration in solution and adsorbent loading capacity at the external particle surface. The equilibrium is assumed to be described by the general isotherm [130]. According to Glueckauf's approach, the intraparticle mass transfer coefficient (K_{s}^{*}) can be defined as shown in Eq. (44) [130]:

$$K_{\mathrm{s}}^{*} = \frac{15\ D_{\mathrm{s}}}{r_{\mathrm{p}}^{2}} \qquad \text{Eq. (44)}$$

where r_{p} is the particle radius and D_{s} is the surface diffusion coefficient. When pore diffusion contributes significantly to the system, the modified mass transfer coefficient is rewritten as shown in Eq. (45):

$$K_s^* = \frac{15\,D_{s,eff}}{r_p^2} = \frac{15\,D_s}{r_p^2} + \frac{15\,D_p}{r_p^2} + \frac{c_0}{\rho_p q_0} \quad \text{Eq. (45)}$$

where $D_{s,eff}$ is the effective surface diffusion coefficient, D_p is the pore diffusion coefficient, and ρ_p is the particle density. Like the HSDM, to facilitate its mathematical application, the terms of the LDF model are transformed into dimensionless forms which are presented in Table S2 [130].

To numerically solve a set of partial and algebraic differential equations it is necessary to group them and transform them into ordinary differential equations through discretization methods [130]. The discretized equations are easily solved with computational tools. Among the methods used are the line method to discretize the partial differential equations in space, apply the finite difference method or the orthogonal placement method to discretize the equation that describes the fluid phase, and obtain the discretization of the adsorbent particle model [130]. By using the dimensionless parameters, the equations of the LDF model can be written as shown in Eqs. (46)-(51) [130]:

Table S2. Dimensionless parameters used in the LDF model

Dimensionless parameter	Symbol	Definition
Dimensionless concentration	X	$X = \frac{C}{C_0}$
Dimensionless adsorbent loading	Y	$Y = \frac{q}{q_0}$
Dimensionless distance	S	$S = \frac{Z}{h}$
Dimensionless time (throughput ratio)	T	$T = \frac{t}{t_b^{id}} \approx \frac{t}{t_{st}}$

Dimensionless mass transfer coefficient (film diffusion)	N_F	$N_\mathrm{F} = \dfrac{K_\mathrm{F}\, a_\mathrm{VR}\, c_0\, t_\mathrm{st}}{\rho_\mathrm{b} q_0}$
Dimensionless mass transfer coefficient (intraparticle diffusion)	N_s	$N_\mathrm{s} = K_\mathrm{s}^{*}\, t_\mathrm{st}$

$$\frac{\partial X}{\partial S} + \frac{\partial \bar{Y}}{\partial T} = 0 \qquad \text{Eq. (46)}$$

$$X(T = 0, S) = 0, \bar{Y}(T = 0, S) = 0 \qquad \text{Eq. (47)}$$

$$X(T, S = 0) = 1 \qquad \text{Eq. (48)}$$

$$\frac{\partial \bar{Y}}{\partial T} = N_\mathrm{F}(X - X_\mathrm{s}) \qquad \text{Eq. (49)}$$

$$\frac{\partial \bar{Y}}{\partial T} = N_\mathrm{s}(Y - Y_\mathrm{s}) \qquad \text{Eq. (50)}$$

$$Y_\mathrm{s} = F(X_\mathrm{S}) \qquad \text{Eq. (51)}$$

Multicomponent adsorption isotherm models

The adsorption isotherm models allow correlating the amount of solute retained by adsorption on a solid phase, or adsorbent, with the solute concentration in the liquid or gaseous phase. Therefore, isotherms provide insights into how adsorption occurs as the solute concentration varies. The parameters of adsorption isotherms are assessed in batch systems and incorporated into mass balance models in adsorption columns. These parameters are essential for adjusting and validating column modeling, enabling predictability of results. Table S3 presents nonlinear equations of multicomponent adsorption isotherm models used in the literature, together with a description of their parameters.

Table S3. Multicomponent adsorption isotherm models with nonlinear equations.

Isotherm model	Model equation	Parameter	Reference
Non-modified Langmuir	$q_{e,i} = \frac{q_{m,i} K_{L,i} c_{e,i}}{1 + \sum_{j=1}^{N} K_{L,j} c_{e,j}}$	$q_{e,i}$: Equilibrium adsorption capacity of each species (mg g^{-1})<br>$c_{e,i}$: Equilibrium concentration of each species in multi-element system (mg L^{-1})<br>$q_{m,i}$: Maximum adsorption capacity of each species (mg g^{-1})<br>$K_{L,i}$: Langmuir isotherm constant of each species (L mg^{-1})	[160]
Extended Langmuir	$q_{e,i} = \frac{q_{max} K_{EL,i} c_{e,i}}{1 + \sum_{j=1}^{N} K_{EL,j} c_{e,j}}$	q_{max}: Maximum adsorption capacity of each species (mg g^{-1})<br>$K_{EL,j}$: Langmuir isotherm constant of each species (L mg^{-1});	[190]
Modified Langmuir	$q_{e,i} = \frac{q_{m,i} K_{L,i} \left(\frac{c_{e,i}}{\eta_{L,i}}\right)}{1 + \sum_{j=1}^{N} K_{L,j} \left(\frac{c_{e,j}}{\eta_{L,i}}\right)}$	$q_{m,i}$: Maximum adsorption capacity of each species (mg g^{-1})<br>$K_{L,i}$: Langmuir isotherm constant of each species (L mg^{-1})<br>$\eta_{L,i}$: Modified Langmuir isotherm constant of each species (dimensionless)	[164]
Extended Freundlich	$q_{e,i} = \frac{K_{F,i} c_{e,i}^{\left(\frac{1}{n_i}\right)+x_i}}{C_{e,i}^{x_i} + y_i C_{e,i}^{z_i}}$<br>$q_{e,j} = \frac{K_{F,j} c_{e,j}^{\left(\frac{1}{n_j}\right)+x_j}}{C_{e,j}^{x_j} + y_j C_{e,j}^{z_j}}$	$K_{F,i}$: Freundlich isotherm constant of each species (mg g^{-1}) (L mg^{-1})$^{1/n}$<br>n_i: Adsorption intensity parameter of each species (dimensionless)<br>z_i, y_i, x_i: Extended Freundlich isotherm constants of each species (dimensionless)	[166]
Extended Langmuir-Freundlich or Extended Sips	$q_{e,i} = q_{S,i} \frac{K_{S,i} c_{e,i}^{\left(\frac{1}{n_{S,i}}\right)}}{1 + \sum_{j=1}^{N} K_{S,j} c_{e,j}^{\left(\frac{1}{n_{S,j}}\right)}}$	$K_{S,i}$: Sips isotherm constant of each species (L mg^{-1})<br>$q_{S,i}$: Maximum adsorption capacity of each species (mg g^{-1})<br>$n_{S,i}$: Heterogeneity factor of the Sips isotherm for each species	[191]

Isotherm model	Model equation	Parameter	Reference
Sheindorf–Rebuhn–Sheintuch	$q_{e,i} = K_{F,i} c_{e,i} \left(\sum_{j=1}^{N} a_{ij} c_{e,j}\right)^{\frac{1}{n_i}-1}$, where $a_{ii} = a_{jj} = 1$<br>$N_i(Q) = \alpha_i\, exp\left(\frac{Q\beta_i}{RT}\right)$<br>$\theta_i(Q) = \frac{K_{L,i} c_{e,i}}{1 + \sum_{j=1}^{N} K_{L,j} c_{e,j}}$, where $K_{L,j} = K_{0j} exp\left(\frac{Q}{RT}\right)$<br>$a_{ji} = {K_{0j}}/{K_{0i}}$, $a_{ji} = {1}/{a_{ij}}$	$K_{L,i}$: Langmuir isotherm constant of each species (L mg^{-1})<br>$K_{F,i}$: Freundlich isotherm constant of each species (mg g^{-1}) (L mg^{-1})$^{1/n}$<br>n_i: Heterogeneity factor of the Freundlich isotherm for each species (dimensionless)<br>a_{ij}: Competition coefficient of each species<br>$N_i(Q)$: Number of sites with energy Q (dimensionless)<br>α_i, β_i: Sheindorf-Rebuhn-Sheintuch isotherm constant of each species<br>θ_i: Coverage of each species at energy level Q<br>R: Ideal gas constant (8.3144 J K^{-1} mol^{-1})<br>T: Temperature (K)	[167]
Modified competitive Redlich-Peterson	$q_{e,i} = \frac{K_{RP,i}\left(\frac{c_{e,i}}{\eta_{RP,i}}\right)}{1 + \sum_{j=1}^{N}\left[\alpha_{RP,i}\left(\frac{c_{e,j}}{\eta_{RP,j}}\right)\right]^{\beta,j}}$	$K_{RP,i}$: Redlich-Peterson isotherm constant of each species (L mg^{-1})<br>$\alpha_{RP,i}$: Redlich-Peterson isotherm constant of each species (mg L^{-1})$^{\beta,i}$<br>β, j: Redlich-Peterson isotherm constant of each species (L mg^{-1})	[192]
Ideal adsorption solution theory (IAST)	$C_e = C_e^0(T, P, \Psi) x_i$<br>$q_{e,i} = q_T x_i$<br>$\sum_{i=1}^{n} x_i d\, ln\, \gamma_i = \left(\frac{1}{q_T} - \sum_{i=1}^{n} \frac{x_i}{q_{e,i}^0}\right) d(\Psi)$<br>$\Psi_i = \frac{\Pi_i^0 A}{RT} = \int_0^{C_{e,i}^0} \frac{q_{e,i}^0(C_{e,i}^0)}{C_{e,i}^0} dC_{e,i}^0$<br>$x_T = \sum_{i=1}^{N} x_i$	C_e: Equilibrium liquid concentration of each adsorbate (mmol L^{-1})<br>$C_{e,i}^0$: Liquid phase concentration in equilibrium with resulting monocomponent adsorption capacity (mmol L^{-1})<br>$q^0_{e,i}$: Adsorption capacity (mmol g^{-1})<br>Ψ_i: Reduced spreading pressure (dimensionless)<br>γ_i: Activity coefficient of the adsorbed component (dimensionless)<br>x_i: Mole fraction of each component adsorbed on the adsorbent (dimensionless)<br>q_T: Total adsorbate concentration on the solid phase (mmol g^{-1})	[23]

Isotherm model	Model equation	Parameter	Reference
	$C_{0,i} = C_{e,i} + \frac{w}{V} q_{e,i}$<br>$q_{e,i} = f(Q_{max,i}, b_i, n_i, C^0_{e,i})$	x_T: Total mole fraction adsorbed on the solid phase in a multicomponent system (dimensionless)<br>A: Specific surface area per unit weight of the adsorbent ($m^2\ g^{-1}$)<br>R: Universal gas constant ($J\ mol^{-1}\ K^{-1}$)<br>T: Absolute temperature (K)<br>w: Weight of adsorbent (g)<br>V: Liquid-phase volume (L)<br>$Q_{max,i}$: Maximum adsorption capacity obtained from the isotherm model ($mmol\ g^{-1}$)<br>b_i: Binding constant related to the affinity of the adsorption sites for the component i obtained from the isotherm model ($L\ mmol^{-1}$)<br>n_i: Parameter indicative of surface heterogeneity (dimensionless)	
Real adsorption solution theory (RAST)	$C_e = C^0_e(T, P, \Psi) x_i \gamma_i$<br>$\gamma_i = \gamma^0_i(T, P, \Psi)$<br>$ln\gamma_i = [1 - e^{c\Psi}] \left[1 - ln(x_i + x_j \Lambda_{ij}) - \left(\frac{x_i}{x_i + x_j \Lambda_{ij}} + \frac{x_j \Lambda_{ij}}{x_j + x_i \Lambda_{ji}}\right)\right]$	Ψ: Reduced spreading pressure (dimensionless)<br>γ_i: Activity coefficient of each species (dimensionless)<br>x_i, x_j : Mole fraction of each species (dimensionless)<br>c: Adjustable model parameter (dimensionless)<br>Λ_{ji}: Interaction parameter of each species (dimensionless)	[170]
P-Factor model	$q_{e,i} = \frac{1}{P_i} \frac{k_{L,i} c_{e,i}}{1 + a_{L,i} c_{e,i}}$<br>$P_i = \frac{\left(\frac{k_{L,i}}{a_{L,i}}\right)_{mono}}{\left(\frac{k_{L,i}}{a_{L,i}}\right)_{multi}} = \frac{q_{m,mono}}{q_{m,multi}}$	$K_{L,i}$: Langmuir isotherm constant of each species ($L\ mg^{-1}$) (obtained by fitting Langmuir model to mono- and multicomponent equilibrium data)<br>$a_{L,i}$: Langmuir isotherm constant of each species ($L\ mg^{-1}$)<br>P_i: P_i factor (obtained by fitting Langmuir model to mono- and multicomponent equilibrium data)	[168]

Isotherm model	Model equation	Parameter	Reference
		$q_{\mathrm{m,mono}}$: Langmuir monocomponent maximum adsorption capacity (mg g^{-1}) (obtained by fitting Langmuir model to mono-component equilibrium data)<br>$q_{\mathrm{m,multi}}$: Langmuir multicomponent maximum adsorption capacity (mg g^{-1}) (obtained by fitting Langmuir model to multi-component equilibrium data)	

Monocomponent column adsorption models

Column adsorption systems containing only one adsorbate species can be described using simpler analytical equations with more direct mathematical solutions. Table S4 presents the main column adsorption models in their linear form for single-component systems.

Table S4. Monocomponent column adsorption models in their linear forms.

Isotherm model	Model equation	Parameter	Reference
Bohart-Adams	$ln\left(\frac{C_0}{C}-1\right)=\frac{k_{BA}N_0L}{u}-k_{BA}C_0t$	k_{BA}: Bohart-Adams (BA) rate constant (L mg^{-1} min^{-1})<br>N_0: Adsorption capacity of the adsorbent per unit volume of the bed (mg L^{-1})<br>u: Superficial velocity (cm min^{-1})<br>L: Bed depth (cm)<br>C: output solute concentration (mg L^{-1})<br>C_0: input solute concentration (mg L^{-1})	[11]
Bed Depth Service Time (BDST)	$t=\frac{N_0}{c_0u}L-\frac{1}{k_{BDST}C_0}ln\left(\frac{C_0}{C}-1\right)$	k_{BDST}: Bed depth service time rate constant (L mg^{-1} min^{-1})<br>N_0: Adsorption capacity of the adsorbent per unit volume of the bed (mg L^{-1})<br>u: Superficial velocity of feed to bed (cm min^{-1})<br>C: output solute concentration (mg L^{-1})<br>C_0: input solute concentration (mg L^{-1})	[193]
Thomas	$ln\left(\frac{C_0}{C}-1\right)=\frac{k_{Th}q_0M}{Q}-k_{Th}C_0t$	k_{Th}: Thomas (Th) rate constant (L mg^{-1} min^{-1})<br>q_0: Solid loading per unit weight of adsorbent (mg g^{-1})<br>M: Weight of adsorbent (mg)<br>Q: Volumetric flow rate (L min^{-1})<br>C: output solute concentration (mg L^{-1})<br>C_0: input solute concentration (mg L^{-1})	[12]
Yoon-Nelson	$ln\left(\frac{C_0}{C}-1\right)=k_{YN}\tau-k_{YN}t$	k_{YN}: Yoon-Nelson rate constant (min^{-1})<br>τ: Time required to reach 50% breakthrough time (min)<br>C: output solute concentration (mg L^{-1})<br>C_0: input solute concentration (mg L^{-1})	[13]
Wolborska	$ln\left(\frac{C_0}{C}\right)=\frac{\varepsilon\beta L}{u}-\frac{\varepsilon\beta C_0t}{N_0}$	β: External mass transfer coefficient (min^{-1})<br>L: Bed depth (cm)<br>ε: Bed porosity<br>u: Superficial velocity of feed to bed (cm min^{-1})<br>N_0: Adsorption capacity of the adsorbent per unit volume of the bed (mg L^{-1})<br>C: output solute concentration (mg L^{-1})<br>C_0: input solute concentration (mg L^{-1})	[14]

Table S5. Properties of divalent cations used to predict the affinity order for the surface active sites of bioadsorbents.

Element	*X*	r [a] / Å	r_H [a] / Å	pK_{sp} (PO_4^{3-}) [b]	E^o / V [b]	β [b]	Z/r [c]
Fe(II)	1.830	0.800	4.28	-	−1.170	4.340	0.467
Mn(II)	1.550	0.800	4.28	-	−1.180	2.730	0.467
Zn(II)	1.650	0.740	4.30	32.04	−0.763	5.770	0.465
Cd(II)	1.690	0.970	4.26	32.60	−0.403	5.470	0.469
Pb(II)	1.870	1.320	4.01	42.10	−0.125	0.000	0.499
Co(II)	1.880	0.720	4.23	34.69	−0.277	5.910	0.473
Ni(II)	1.910	0.700	4.04	31.32	−0.257	7.520	0.495
Cu(II)	2.000	0.720	4.19	36.85	−0.340	10.67	0.477
Hg(II)	2.000	1.020	4.22	-	0.796	14.30	0.474

χ = electronegativity (Pauling), r =ionic radius, r_H = hydrated ionic radius, $pK_{sp} = -\log K_{sp}$ (solubility product constant of phosphate salts), E^o = standard reduction potential, β = cumulative complex formation constants with ethylenediamine, Z/r = ionic potential.

[a] [152, 153], [b] [154], [c] [153].

References

[11] Bohart, G.S.; Adams, E.Q. Some aspects of the behavior of charcoal with respect to chlorine.1. *J. Am. Chem. Soc.* **1918**, *42*, 523–544. https://doi.org/10.1021/ja01448a018.

[12] Thomas, H.C. Heterogeneous ion exchange in a flowing system. *J. Am. Chem. Soc.* **1944**, *66*, 1664–1666. https://doi.org/10.1021/ja01238a017.

[13] Yoon, Y.H.; Nelson, J.H. Application of gas adsorption kinetics. I. A theoretical model for respirator cartridge service life. *Am. In. Hyg. Assoc. J.* **1984**, *45*, 509–516. https://doi.org/10.1080/15298668491400197.

[14] Wolborska, A. Adsorption on activated carbon of *p*-nitrophenol from aqueous solution. *Water Res.* **1989**, *23*, 85–91. https://doi.org/10.1016/0043-1354(89)90066-3.

[23] Myers, A.L.; Prausnitz, J.M. Thermodynamics of mixed-gas adsorption. *AIChE J.* **1965**, *11*, 121–127. https://doi.org/10.1002/aic.690110125.

[61] de Almeida, F.T.R.; Elias, M.M.C.; Xavier, A.L.P.; Ferreira, G.M.D.; Silva, I.A.; Filgueiras, J.G.; de Azevedo, E.R.; da Silva, L.H.M.; Gil, L.F.; Gurgel, L.V.A. Synthesis and application of sugarcane bagasse cellulose mixed esters. Part II: Removal of Co^{2+} and Ni^{2+} from single spiked aqueous solutions in batch and continuous mode. *J. Colloid Interface Sci.* **2019**, *552*, 337–350. https://doi.org/10.1016/j.jcis.2019.05.046.

[105] Xavier, A.L.P.; Adarme, O.F.H.; Furtado, L.M.; Ferreira, G.M.D.; da Silva, L.H.M.; Gil, L.F.; Gurgel, L.V.A. Modeling adsorption of copper(II), cobalt(II) and nickel(II) metal ions from aqueous solution onto a new carboxylated sugarcane bagasse. Part II: Optimization of monocomponent fixed-bed column adsorption. *J. Colloid Interface Sci.* **2018**, *516*, 431–445. https://doi.org/10.1016/j.jcis.2018.01.068.

[130] Worch, E. *Adsorption Technology in Water Treatment: Fundamentals, Processes, and Modeling*, 1st ed.; De Gruyter: Berlin, Germany, 2012.

[138] McCuen, R.H.; Surbeck, C.Q. An alternative to specious linearization of environmental models. *Water Res.* **2008**, *42*, 4033–4040. https://doi.org/10.1016/j.watres.2008.05.030.

[152] Goel, J.; Kadirvelu, K.; Rajagopal, C. Competitive sorption of Cu(II), Pb(II) and Hg(II) ions from aqueous solution using coconut shell-based activated carbon. *Adsorp. Sci. Technol.* **2016**, *22*, 257–273. https://doi.org/10.1260/0263617041503453.

[153] Nightingale, E.R., Jr. Phenomenological theory of ion solvation. Effective radii of hydrated ions. *J. Phys. Chem.* **2002**, *63*, 1381–1387. https://doi.org/10.1021/j150579a011.

[154] Speight, J.G. *Lange's Handbook of Chemistry*, 16th ed.; McGraw-Hill Professional: New York, NY, USA, 2005.

[160] Butler, J.A.V.; Ockrent, C. Studies in electrocapillarity. III. *J. Phys. Chem.* **1930**, *34*, 2841–2859. https://doi.org/10.1021/j150318a015.

[164] Schay, G.J.; Fejes, F.P.; Szethmary, J. Adsorption of gases and Gas Mixtures. *Acta Chim. Acad. Sci. Hung.* **1957**, *12*, 299–308.

[166] Fritz, W.; Schluender, E.U. Simultaneous adsorption equilibria of organic solutes in dilute aqueous solutions on activated carbon. *Chem. Eng. Sci.* **1974**, *29*, 1279–1282. https://doi.org/10.1016/0009-2509(74)80128-4.

[167] Sheindorf, C.; Rebhun, M.; Sheintuch, M. A Freundlich-type multicomponent isotherm. *J. Colloid Interface Sci.* **1981**, *79*, 136–142. https://doi.org/10.1016/0021-9797(81)90056-4.

[168] McKay, G.; Al Duri, B. Simplified model for the equilibrium adsorption of dyes from mixtures using activated carbon. *Chem. Eng. Process. Process Intensif.* **1987**, *22*, 145–156. https://doi.org/10.1016/0255-2701(87)80041-7.

[170] Costa, E.; Sotelo, J.L.; Calleja, G.; Marrón, C. Adsorption of binary and ternary hydrocarbon gas mixtures on activated carbon: Experimental determination and theoretical prediction of the ternary equilibrium data. *AIChE J.* **1981**, *27*, 5–12. https://doi.org/10.1002/aic.690270103.

[189] Myers, R.H.; Montgomery, D.C.; Anderson-Cook, C.M. *Response surface methodology process and product optimization using designed experiments*, 4th ed.; John Wiley & Sons, Inc., Hoboken, USA, 2016; pp. 1-12.

[190] Yang, R.T. *Gas Separation by Adsorption Processes*, Butterworth-Heinemann, Stoneham, USA, 1987; pp. 49-100. https://doi.org/10.1016/c2013-0-04269-7.

[191] Sips, R. On the Structure of a Catalyst Surface, *J. Chem. Phys.* **1948**, *16*(5), 490-495. https://doi.org/10.1063/1.1746922.

[192] Redlich, O.; Peterson D. L. A Useful Adsorption Isotherm, *J. Phys. Chem.* **1959**, *63*, 6, 1024. https://doi.org/10.1021/j150576a611.

[193] Hutchins, R.A. New simplified design of activated. carbon systems. *Chem. Eng* **1973**, *80*(9), 133-138.
